# A comprehensive method to elucidate pyoverdines produced by fluorescent *Pseudomonas* spp. by UHPLC-HR-MS/MS

**DOI:** 10.1007/s00216-022-03907-w

**Published:** 2022-01-27

**Authors:** Karoline Rehm, Vera Vollenweider, Rolf Kümmerli, Laurent Bigler

**Affiliations:** 1 Department of Chemistry, University of Zurich, Winterthurerstr. 190, 8057 Zurich, Switzerland; 2 Department of Quantitative Biomedicine, University of Zurich, Winterthurerstr. 190, 8057 Zurich, Switzerland

**Keywords:** Siderophore, Pyoverdine, Ferribactin, Isopyoverdine, *Pseudomonas*, UHPLC-MS, High-throughput structure elucidation pipeline

## Abstract

Microbial secondary metabolites represent a rich source for drug discovery, plant protective agents, and biotechnologically relevant compounds. Among them are siderophores, iron-chelating molecules, that show a great influence on bacterial community assembly and the potential to control pathogen invasions. One of such a siderophore is pyoverdine that is produced by fluorescent *Pseudomonas* members and consists of different peptide chains specific to each bacterial species. The identification and structural elucidation of such suites of siderophores remain widely underexplored as general high-throughput analytical protocols are missing. Therefore, a dedicated method was established allowing a rapid localization and structural elucidation of pyoverdines. Liquid bacterial culture samples were purified by an easy small-scale solid-phase extraction (SPE). Ultra-high-performance liquid chromatography high-resolution tandem mass spectrometry (UHPLC-HR-MS/MS) separated highly polar pyoverdines and their derivatives. All ion fragmentation (AIF) generated mass spectra containing the characteristic fragments of the biological precursor of pyoverdine, ferribactin. This led to the revelation of the mass of secreted pyoverdines. Targeted MS/MS experiments at multiple collision energies accomplished the full structure elucidation of the pyoverdine peptide chain. A mass calculator and a fragmentation predictor facilitated greatly the interpretation of MS/MS spectra by providing accurate masses for a straightforward comparison of measured and theoretical values. The method was successfully validated using four well-known pyoverdines with various peptide chains. Finally, the applicability was proven by the analysis of 13 unknown pyoverdines secreted by sampled bacterial cultures. Among these, 4 novel pyoverdine peptide chains were discovered and are herein reported for the first time.

**Graphical abstract:**

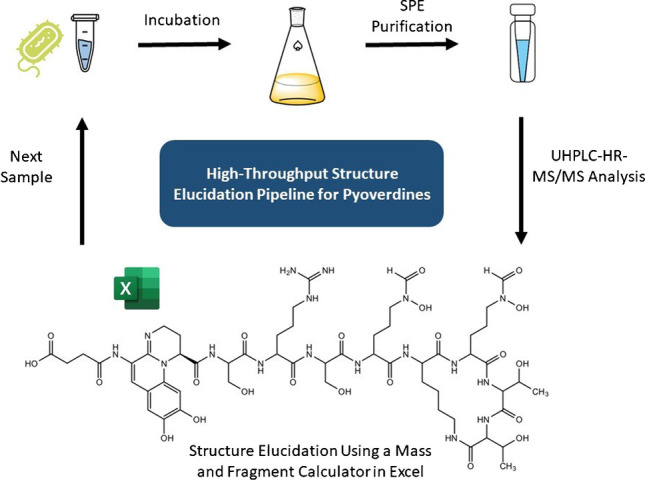

**Supplementary Information:**

The online version contains supplementary material available at 10.1007/s00216-022-03907-w.

## Introduction

Microorganisms produce a vast number of secondary metabolites that play key roles in microbial physiological processes, and for growth and survival. Secondary metabolites are low-molecular-weight compounds with various bioactive properties specific to the species that exudes them. Among them are toxins to control competing organisms, compounds for metal transport, quorum-sensing molecules for chemical communication, biosurfactants to modulate group motility, and many more [1–5].

Over the last years, the interest for microbial secondary metabolites increased immensely. They serve as a primary source for drug discovery resulting in novel antibiotics, antitumor drugs, or immunosuppressants [6, 7]. In the field of biotechnical engineering, biosurfactants gained popularity as a sustainable and environmentally friendly alternative to synthetic surfactants [8]. Last but not least, iron-chelating metabolites are utilized for medicinal and agricultural applications [9]. These iron carriers are called siderophores, possess a high iron-binding affinity (> 10^30^ M^−1^), and are with over 500 structures chemically diverse [10]. Recent studies revealed the great potential of siderophores as probiotics to protect plants from pathogen infections [11] and to protect bacterial communities from pathogen invasion [12]. Despite the clear efficiency of certain siderophores to inhibit pathogen growth, the chemical structure of these siderophores remains mostly unknown.

To facilitate research on bacterial secondary metabolites, we present a novel method based on UHPLC-MS/MS for the high-throughput characterization of pyoverdines from crude bacterial culture extracts. Pyoverdines are iron-scavenging chromopeptides and are a siderophore solely produced by fluorescent *Pseudomonas* species [13]. Each fluorescent *Pseudomonas* species produces its own type of pyoverdine varying in length and amino acid sequence. This is why over 60 pyoverdines were discovered until today and many unknown pyoverdine types are yet to be characterized [14]. They are a major force that can promote or impede microbial invasions in bacterial communities. However, the chemical structures of promoting or inhibiting pyoverdines were not elucidated in the respective studies [11, 12] as their characterization is demanding and time consuming.

Nuclear magnetic resonance (NMR) spectroscopy and tandem mass spectrometry (MS/MS) deliver the highest level of confidence for a full characterization of a secreted pyoverdine. However, bacterial liquid cultures are complex mixtures requiring a tedious purification of their components before analysis. Furthermore, a bacterium is not only producing a single pyoverdine structure but also multiple derivatives and related compounds complicating the matter.

**Fig. 1:**
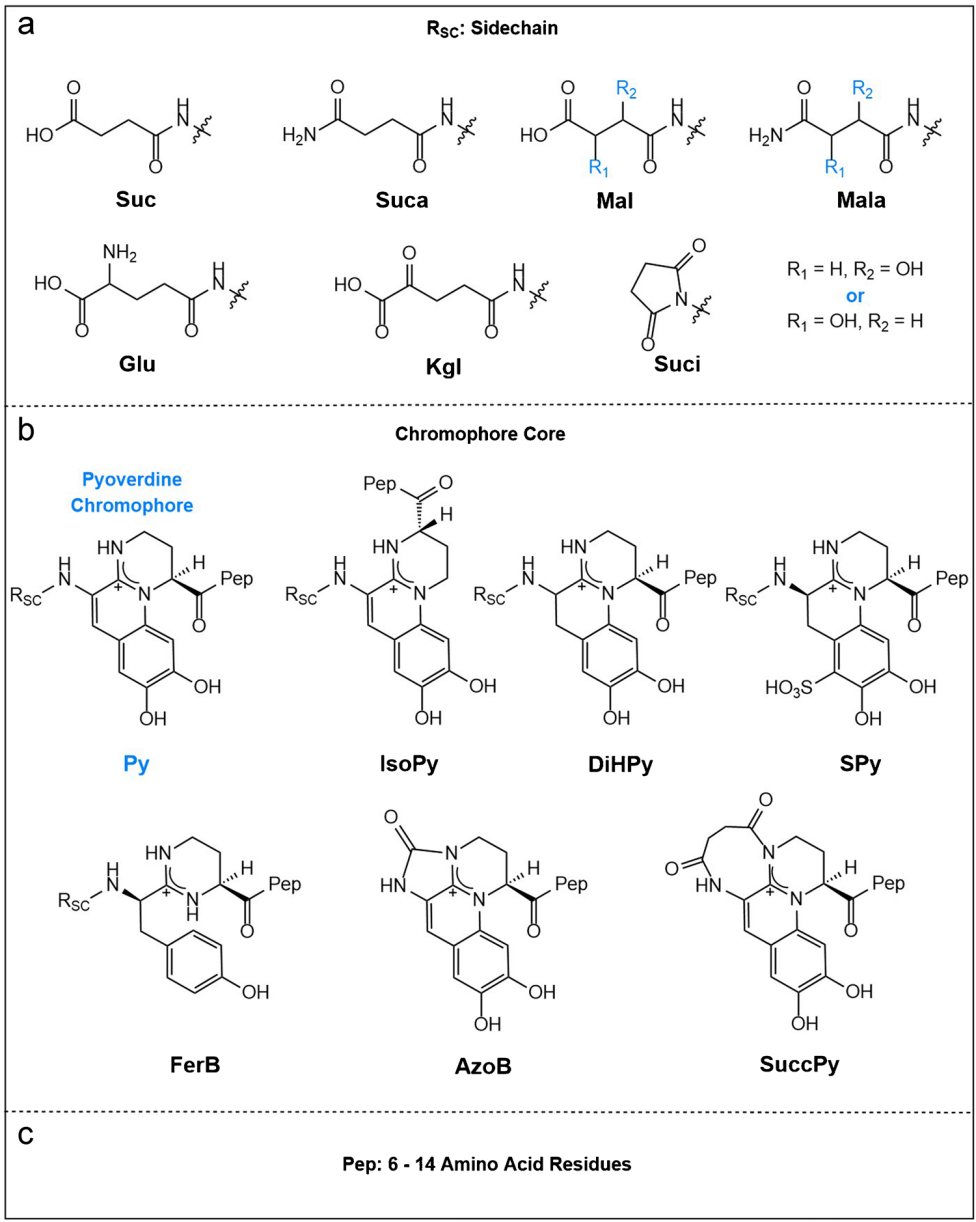
Building blocks of pyoverdines and their derivatives. **a** Varieties of acyl side chains found in bacterial extracts. Note: Position of the hydroxy group of 3/4 (highlighted in blue) is not resolved and could be either one of the two positions [17]. **b** Varieties of chromophores encountered in natural occurring pyoverdine derivatives [16]. **c** Structurally versatile peptide chain varied by bacterial strain

Taking a closer look at the chemical structure of these compounds (Fig. 1), pyoverdines and their derivatives can be defined as chromopeptides that can be separated into three parts: a sidechain R_SC_ (Fig. 1a), a chromophore core (Fig. 1b), and a peptide part Pep (Fig. 1c) [15]. In bacterial extracts, variations of the structure in all three parts have been found. For the side chain, not only succinic acid (Suc) was identified but also succinic amide (Suca), malic acid/amide (Mal, Mala), α-ketoglutaric acid (Kgl), glutamic acid (Glu), and a succinic imide derivative (Suci). Overall, succinic acid and succinic amide are the most frequently encountered side chains [15]. Pyoverdine itself is only characterized by a 2,3-diamino-6,7-dihydroxyquinoline unit (Py) but certain biological precursors and other modifications are also commonly detected. Among them are isopyoverdine (IsoPy), dihydro-pyoverdine (DiHPy), dihyodro-pyoverdine-7-sulfonic acid (SPy), ferribactin (FerB), azotobactin (AzoB), and succinopyoverdine (SuccPy) [16]. Finally, a linear or cyclic peptide chain consisting of 6–14 amino acid residues is linked to the core. While the sidechain and the chromophore units are mostly conserved, the sequence of the peptide chain varies greatly depending on the bacterial species.

The most popular and established approach to determine the structure of pyoverdines is based on electrospray ionization (ESI) in combination with collision-induced dissociation of selected ions [15, 18, 19]. For the chromophore core, characteristic fragments are used for its identification. Apart from that, mostly B and Y″ fragment ions are detected that allow the elucidation of the peptide chain [15].

This structure elucidation is a complex task as pyoverdine and its derivatives are often detected as a mixture containing various side chains, chromophores and in some cases both linear and cyclized entities. Purification of pyoverdines has been mostly conducted by large-scale solid-phase purification on a XAD-4 resin [20] or by semi-preparative HPLC [21] consuming time and resources. The structure of the compounds of interest was then investigated with MS/MS by continuous flow injection without prior LC separation [22–24]. In the LC–MS/MS studies to date, crude extracts were analyzed, whereby only a few specific pyoverdines have been investigated [18, 19]. Overall, the need of further method optimization and an easy protocol for fragmentation interpretation is required to ensure the accessibility of high-throughput structure elucidation.

In this work, a generalized high-throughput UHPLC-MS/MS pipeline was developed to characterize pyoverdines and their derivatives in bacterial liquid cultures. Four well-known pyoverdines differing in their peptide chain and C-terminal end were used to optimize purification, LC, and MS conditions and to validate the procedure. Minimal sample amounts of bacterial cultures (500 μL) were extracted by solid-phase extraction (SPE) and were directly submitted to liquid chromatography. The polar components of the bacterial extracts were separated with a high peak capacity and analyzed with MS/MS in only 15 min per sample. The high mass accuracy of a Q Exactive MS instrument, pyoverdine fragmentation at multiple collision energies, and a fully exploited duty cycle ensured a high sensitivity and simplified annotation of B and Y″ ions. For a rapid MS/MS interpretation, a new pyoverdine fragmentation predictor programmed in Excel was created allowing a quick comparison between measured and theoretical masses. Applicability of the method was proven by successfully analyzing the unknown pyoverdines found in the bacterial extract of 13 fluorescent *Pseudomonas* species.

## Materials and methods

### Chemicals and reagents

Commercial pyoverdines from *P. fluorescens* with reported structures (Py SA, CAS RN 8062–00-8, > 90%) and 2,2′-bipyridine (≥ 99%) were obtained from Sigma Aldrich (Buchs, SG, Switzerland). Acetonitrile (CH_3_CN) and methanol (MeOH) were obtained from Biosolve (ULC grade, Valkenswaard, Netherlands) and formic acid from Fluka (LC–MS grade, Buchs, SG, Switzerland). Ultrapure water (< 2 ppb TOC) was produced using a Milli-Q^®^ Advantage A10 water purification system (Merck, Bedford, MA, USA). Pierce LTQ Velos ESI Positive Ion Calibration Solution was purchased from Thermo Fisher (Waltham, MA, USA). For the growth of bacteria, a casamino acid solution (CAA) was used as a nutrient medium. This aqueous solution (1 L) was made from vitamin-free casein acid hydrolysate (10.0 g), K_2_HPO_4_ · 3H_2_O (1.18 g), and MgSO_4_ · 7H_2_O (0.25 g). The listed ingredients were bought from Sigma Aldrich (Buchs, SG, Switzerland).

### Bacterial cultures

All cultures originated from the Rolf Kümmerli strain collection (University of Zurich) and were stored as glycerol stocks at − 80°. Among them were the *P. aeruginosa* strains PAO1, 1–60, and 206–12 producing pyoverdine types 1, 2, and 3, respectively [25, 26]. As the chemical structures of all three types have been characterized, the pyoverdines of these strains were used together with the commercially available pyoverdine (Py SA) to establish and verify our structure elucidation pipeline. Furthermore, 13 *Pseudomonas* environmental isolates sampled from soil and pond habitats on the campus of the University of Zurich Irchel (47.40° N, 8.54° E; Switzerland) were investigated. Their sampling procedure and isolation can be viewed in [27]. These environmental isolates are known to produce pyoverdine, but it is unknown which type they make.

### Growth of liquid cultures

Bacteria were transferred from the glycerol stock solution into a Falcon tube containing lysogeny broth (8 mL) using a sterile inoculation loop and were incubated overnight at 28 °C under vigorous shaking (170 rpm). Next, the cultures were centrifuged (7500 rcf, 3 min) and the supernatant was discarded. The remaining pellet was washed twice with 0.8% NaCl (8 mL) by vortexing, subsequent centrifugation (7500 rcf, 3 min), and removal of the wash solution. An aqueous NaCl solution (0.8%) was added to the washed pellet until an optical density at 600 nm (OD600) of 1 absorbance unit (AU) was reached. 2 mL of the resuspended culture were added to 5% CAA (500 mL) supplemented with 250 μM 2,2′-bipyridine (1.25 mL), an iron chelator that induces pyoverdine production in all *Pseudomonas* isolates, and grown at 28 °C under vigorous shaking (170 rpm). After 72–120 h, the liquid culture was centrifuged (7500 rcf, 3 min) and the pyoverdine containing supernatant was sterile-filtered through a 0.22 μm filter before storage at − 20 °C.

### Sample preparation

All biological samples were extracted by SPE using Strata-X (1 mL, 30 mg) polymeric reversed-phase cartridges (Phenomenex, Torrance, CA, USA). The cartridges were conditioned with 1 mL MeOH followed by equilibration with 1 mL H_2_O. A solution of 5 µL concentrated formic acid was added to the thawed supernatant samples (500 µL) inside an Eppendorf vial and vortexed for 5 s. The samples were then loaded onto the sorbent and subsequently washed with H_2_O (2 × 0.3 mL). The pyoverdine fractions were finally eluted with H_2_O/MeOH 7:3 + 0.1% HCOOH (2 × 0.3 mL). No further reconstitution was performed and the elute was either directly injected for measurement or stored at − 20 °C until use.

The pyoverdine reference material purchased from Sigma Aldrich (Py SA) was dissolved in H_2_O/MeOH 1:1 at 50 μg/mL concentration.

Between 5 and 20 µL of the sample was injected for analysis.

### Chromatography

Liquid-chromatography was performed on an UltiMate 3000 UHPLC (Thermo Fisher, Waltham, MA, USA) build from a binary RS pump, an XRS open autosampler, and a temperature-controllable RS column oven. Chromatographic separation was achieved at 25 °C on an ACQUITY UPLC HSS C18 Column (100 Å, 1.8 µm, 2.1 × 100 mm, Waters, Milford, USA). Eluent A consisted of H_2_O + 0.1% HCOOH and B of CH_3_CN + 0.1% HCOOH. The following gradient was applied at a constant flowrate of 0.4 mL: (i) 4% B isocratic from 0.0–0.5 min; (ii) linear increase to 11% B until 5.25 min; (iii) linear increase to 95% until 7.0 min; (iv) holding 95% B until 10.0 min; (vi) back to the starting conditions of 4% B until 10.5 min; and (vii) equilibration for 4.5 min until the next run.

### Mass spectrometry

The UHPLC was coupled to a Q Exactive hybrid quadrupole-Orbitrap mass spectrometer (Thermo Fisher Scientific, Waltham, MA, USA) equipped with a heated ESI source (position B). The (+)-ESI parameters were as followed: spray voltage 3.5 kV, sheath gas 50 L/min, auxiliary gas 13 L/min, sweep gas 3 L/min (N_2_), capillary temperature 260 °C, auxiliary gas temperature 450 °C, S-lens RF level 55.0. Samples were measured under two different conditions: (1) Alternative full scan (FS)/all ion fragmentation (AIF, higher-energy collisional dissociation of all ionized molecules without prior mass filtering), 140,000 full width half maximum resolution (FWHM at *m/z* 200), IT_max_ 500 ms, AGC target 3e6 for FS as well as AIF, mass ranges from *m/z* 140–2000 (FS) and *m/z* 133.3–1900 (AIF), and normalized collision energy (NCE) of 50. (2) Alternative FS/parallel reaction monitoring (PRM), resolution of 70,000 FWHM with IT_max_ 200 ms (FS) and 35,000 FWHM with IT_max_ 110 ms PRM (PRM), AGC target of 3e6 (FS) and 5e5 (PRM), non-stepped NCE at 20, 25, 30, 35, 50, and 100, loop count 12. The targeted ions that were used for fragmentation and elucidation of the pyoverdine peptide chains of the bacterial extracts analyzed in this work can be found in the supplementary Table S1.

Using a Pierce LTQ ESI positive ion calibration solution (Thermo Fisher Scientific, Waltham, MA, USA) for external calibration, a mass deviation below 5 ppm was tolerated. Xcalibur 4.1 was used for data acquisition and interpretation. An Excel calculator was used to predict the *m/z* values of masses and fragments of pyoverdines and related compounds (available in the supplementary).

## Results and discussion

### Pyoverdine purification by SPE

To avoid clogging injectors and column frits through proteins and to minimize matrix effects [28], a sample preparation protocol based on SPE was developed. Different SPE sorbents were evaluated: the Strata-X, Strata-X-C, and Strata-X-CW. Thereby, the Strata-X delivered the highest analyte response. Afterwards, the solvent composition for the washing and elution step was adjusted testing various percentages of MeOH in water. No analyte was lost during the washing step with pure H_2_O. Using 30% MeOH in 0.1% formic acid acidified H_2_O for the pyoverdine elution, a recovery of approximately 80% was obtained. It was refrained to further increase the elution strength as interferents from the liquid culture medium were observed that would impact the high robustness of the method.

Using the optimized method, pyoverdines of all bacterial supernatants were purified successfully. Compared to the traditional Amberlite XAD-4 resin purification [15], lower volumes of supernatant (0.5 mL vs. 500 mL), total solvent (3.2 mL vs. > 200 mL), and a shorter time (15 min vs. > 1 h) are necessary per sample. Even more time can be saved by extracting up to 12 supernatants in parallel with one SPE manifold making small-scale SPE purification suitable for high-throughput analysis. Compared to the single available SPE method for pyoverdines [18], only a single washing step and no reconstitution is required.

### Chromatographic separation of pyoverdine extracts

**Fig. 2:**
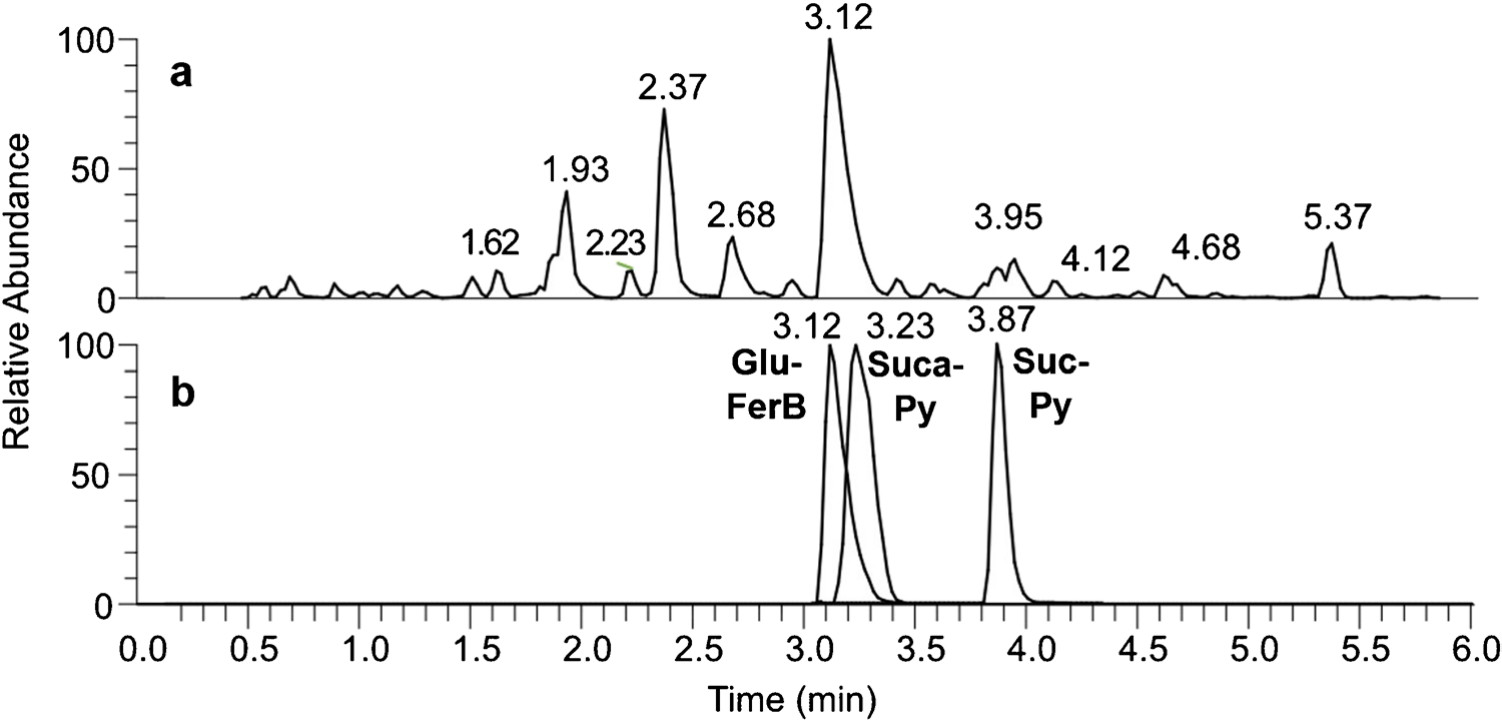
**a** Base peak chromatogram of a SPE purified bacterial extract labeled with the ID S3e20. **b** Normalized extracted ion chromatograms of Glu-FerB (*m/z* 656.31295), Suca-Py (*m/z* 647.28948), and Suc-Py (*m/z* 647.78149) found in the sample S3e20

After testing various column materials as well as eluent additives, the HSS C18 column using 0.1% formic acid as a modifier and CH_3_CN as the organic phase gave the best separation for all extracted samples. All pyoverdines and their derivatives elute in less than 6 min. An exemplary chromatogram of the sample S3e20 is illustrated in Fig. 2a showing the complexity of a crude bacterial extract. Peak shapes are highly symmetrical and sharp as seen in the extracted ion chromatograms of the Glu-FerB, Suc-Py, and Suca-Py compounds found in sample S3e20 (Fig. 2b). In general, peak width ranged from 0.1 to 0.2 min depending on the analyte. Slight peak tailing was observed in few cases like for Glu-FerB in Fig. 2b.

A full list of retention times of all detected Glu-FerB, Suc-Py, and Suca-Py compounds in each bacterial sample is given in the electronic supplementary (Table S2). The identification of the relevant peaks is explained in the next sections. Each compound has a very specific retention time covering a broad range from 1.33 to 5.78 min. All peptides were sufficiently retained on the column material despite their high polarity. A full baseline separation of Suc-Py and its corresponding Suca-Py (with an identical peptide chain) was achieved in all cases (where both analytes were present). Comparing the retention times of all Suca-Py compounds to their succinic acid side chain derivatives, the Suca-Py compounds elute in average around 0.67 ± 0.06 min earlier. Therefore, the retention time of one out of two compounds can be easily estimated from the retention time of the other. In each extract, Glu-FerB were fully or partially separated from their corresponding Suca-Py. Furthermore, Glu-IsoPy and Glu-FerB with the same side chain were also separated in the single bacterial strain (3C16) that produced both compounds. Therefore, a good selectivity of the column material based on the chromophore core is given, too.

Compared to already published methods, we present not only a chromatographic method with a shorter run time but also demonstrate a higher separation power. For example, Wei and Aristilde [19] use a 30 min gradient with a shorter 50 mm Hypersil GOLD column. Baune et al. [18]*,* on the other hand, employ a 150 mm long iHILIC Fusion column taking 40 min for a single run. In both studies, only few selected pyoverdines have been analyzed. Here, we have demonstrated that our separation method is suitable for a general application by analyzing a large number of pyoverdines with various structures and polarities.

### Identifying pyoverdines and their derivatives in complex samples

**Fig. 3:**
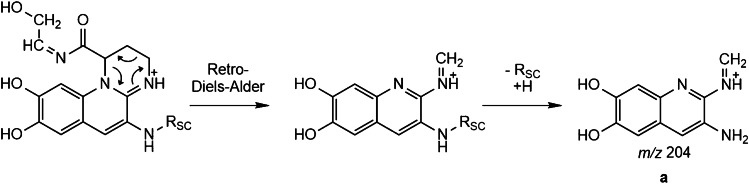
Fragmentation reaction of the pyoverdine chromophore resulting in the characteristic fragment *m/z* 204 (*R*_SC_ = side chain)

Bacterial supernatants are a complex mixture even after a SPE purification (see Fig. 2). A common approach to localize pyoverdines in a HPLC chromatogram is by application of all ion fragmentation (AIF) to generate pyoverdine characteristic fragments and compare their retention time to possible precursor masses such as in the publication of Wei and Aristilde [19] or Budzikiewicz et al. [15]. This fragment is found at *m/z* 204.0768 (C_10_H_10_O_2_N_3_) (**a**). It has its origin in a retro-Diels–Alder reaction of the pyoverdine chromophore, thus making it highly specific (Fig. 3) [16].

**Fig. 4:**
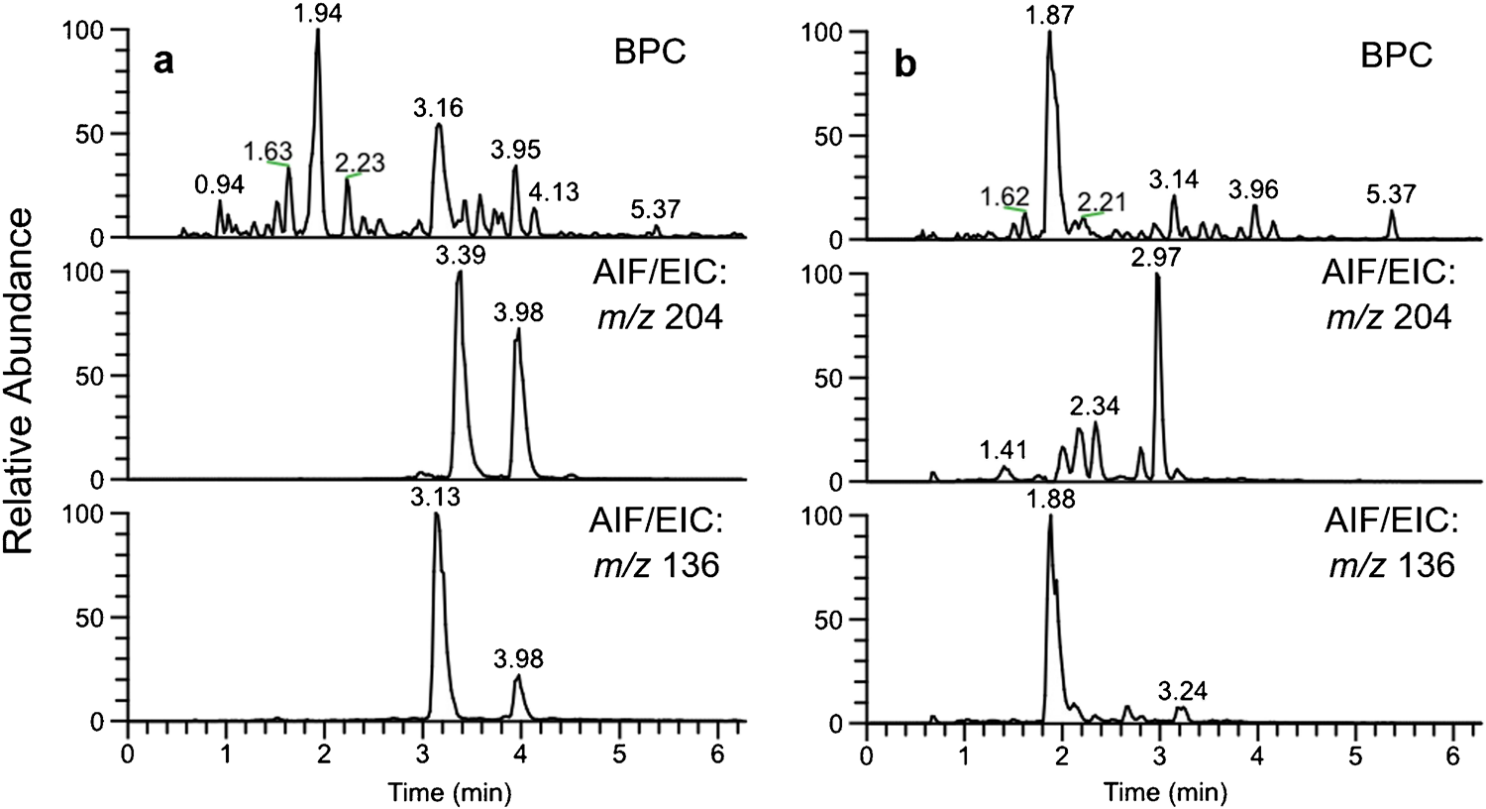
Visual comparison of the base peak chromatograms of the UHPLC-MS analyzed bacterial SPE extracts of PAO1 (**a**) and 3A06 (**b**) together with the extracted ion chromatograms of the fragments characteristic for pyoverdine (*m/z* 204) and ferribactin (*m/z* 136) measured in the AIF experiment

This approach is not straightforward for complex samples containing several types and/or a low quantity of pyoverdines. The problem is exemplified in Fig. 4: While two clear pyoverdine fragment signals are found in the extract of PAO1, up to nine peaks are detected for the extract of 3A06, which are all potential pyoverdine candidates. The fact that pyoverdines might exist with various side chains complicates the finding of a good starting point. Related derivatives such as azotobactin and succinopyoverdin produce also *m/z* 204 fragment ions even if in lower abundance [16]. Fragmenting each potential precursor mass and interpreting their MS/MS spectra would take several measurements and a lot of time.

**Fig. 5:**
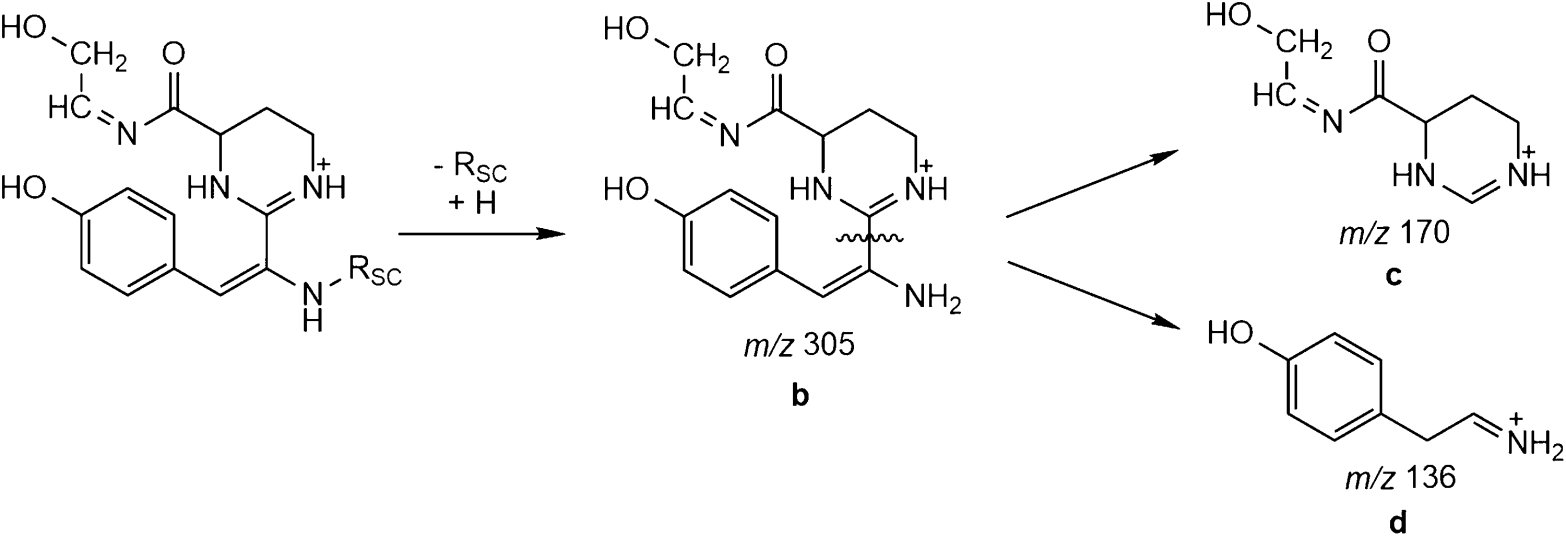
Fragmentation reaction of the ferribactin chromophore resulting in the characteristic fragment tirade *m/z* 305, 170, and 136 (*R*_SC_ = side chain)

Hence, a new procedure was implemented. Referring anew to the work of Budzikiewicz et al. [16], ferribactin, the biological precursor of pyoverdine, exists in almost all natural cases with a glutamic acid side chain (Glu-FerB). Ferribactins give rise to a tirade of characteristic fragments (*m/z* 305.1452 (**b**), 170.0924 (**c**), and 136.0757 (**d**)) as shown in Fig. 5 (adapted from [16]).

In our study, the *m/z* 136 ions were the most abundant in all cases. Extracting this *m/z* value, one dominant peak is visible as in the chromatogram of the PAO1 and the 3A06 extract (see Fig. 4). Knowing the ferribactin precursor mass, the mass for all hypothetical pyoverdines with various side chains can be derived easily. The theoretical mass difference of Glu-FerB to its corresponding Suc-Py and Suca-Py amounts − 17.0629 and − 18.0470 Da, respectively. These pyoverdine masses can be extracted from the TIC. Afterwards, their identity can be confirmed by the pyoverdine characteristic fragment of the 2,3-diamino-6,7-dihydroxyquinoline chromophore at *m/z* 204.0768 (C_10_H_10_O_2_N_3_) in the AIF experiment. This pyoverdine identification approach worked for all samples and simplified the process of picking useful precursor masses for further fragmentation.

**Fig. 6:**
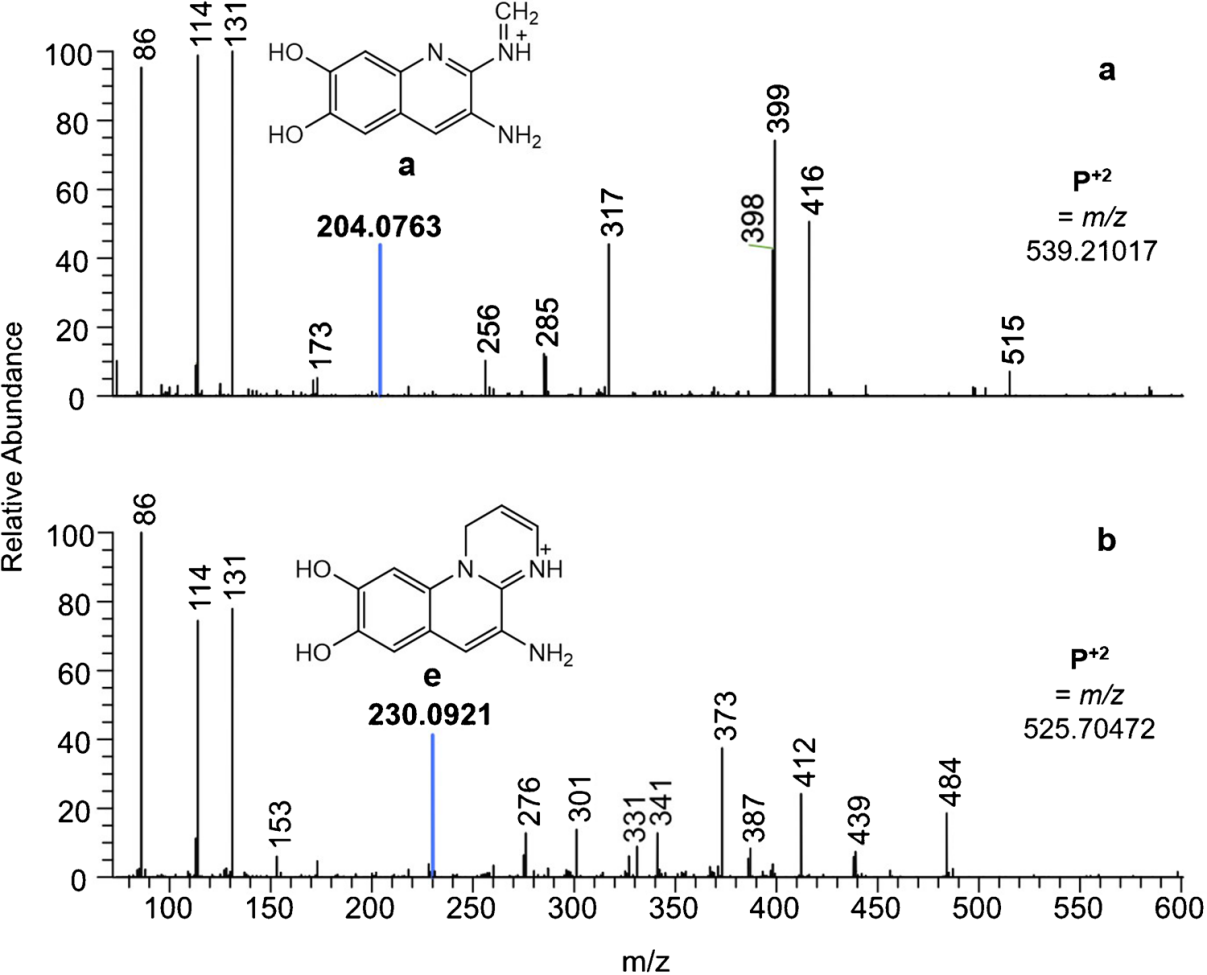
Fragmentation patterns in the lower regions of Suc-Py of S3c13 ([*M* + 2H]^2+^, 539.21017) (**a**) and Glu-IsoPy of 3C16 ([*M* + 2H]^2+^, 525.70472) (**b**) at an NCE of 50 highlighting the characteristic fragment of the chromophore unit

In two cases, an additional advantage of this procedure was found: For the supernatant of 3C16, clear fluorescence under UV light was visible but only a weak *m/z* 204 fragment was detected. The bacterial strain was not discarded as a false assignment to the *Pseudomonas* species as ferribactin was detected. Instead, the possibility of the production of another pyoverdine derivative was considered and indeed, isopyoverdine was found. While only differing in the position of the peptide chain at the chromophore (Fig. 1, IsoPy), isopyoverdine decomposes to the fragment **e** with *m/z* 230.0924 (C_12_H_12_N_3_O_2_^+^) (Fig. 6) [16].

In Fig. 6, the fragmentation pattern in the lower mass range of the isopyoverdine with a glutamic acid side chain of sample 3C16 is compared to the fragmentation pattern of the succinic acid pyoverdine of S3c13. While the overall fragmentation behavior at 50 NCE is similar below *m/z* 250, the striking difference of an intense *m/z* 230 vs *m/z* 204 signal is observable. Hence, the search for ferribactin fragment prevents the overlook of potential pyoverdine derivatives not owning highly abundant *m/z* 204 fragments such as isopyoverdines or pyoverdines with an alpha-ketoglutaric acid side chain [16].

**Fig. 7:**
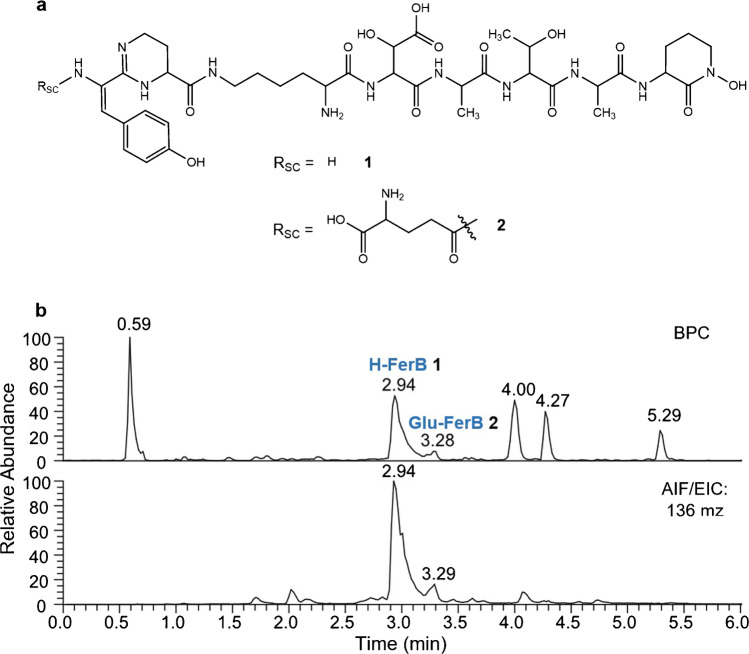
**a** Chemical structure of Glu-FerB (**1**) and H-FerB (**2**) found in the sample S3a20. **b** Comparison of the base peak chromatogram of the full scan and the extracted ion chromatogram of the *m/z* 136 fragment ions for sample S3a20

In a second case, another unexpected finding was encountered. While it is known that in seldom cases ferribactin might exist with a succinic amide side chain [16], a novel ferribactin without any sidechain was detected in sample S3a20 (H-FerB, see Fig. 7a). There, the most intense signal in the AIF belonged to ferribactin with no side chain (**1**) while the signal for the ferribactin with a glutamic acid side chain (**2**) was considerably lower in intensity (Fig. 7b). The identity of the side-chainless ferribactin was later proven by isolated MS/MS fragmentation (see supplementary Table S14) making it the first officially reported side-chainless ferribactin.

### Fragmentation behavior and choice of precursor ions

For the MS/MS fragmentation of pyoverdines and its derivatives, parallel reaction monitoring was employed. Targeting only specific masses, the parent ion can be fragmented into several characteristic product ions inside the HCD cell. Thereby, a monoisotopic isolation is possible with an isolation window up to *m/z* 0.4.

The pyoverdines from Py SA as well as from the SPE purified supernatants of PAO1, 1–60, and 206–12 cultures were used as reference and control compounds as their chemical structures were known in advance (see Table 1). Both cyclic and linear pyoverdines with different peptide chains were hence available to optimize a broad band of collision energies that generates interpretable fragment spectra for all pyoverdine types. The [*M* + 2H]^2+^ ions of Suc-Py from Py SA, PAO1, 1–60, and 206–12 were fragmented stepwise from 20–100 NCE to determine the optimal normalized collision energy (NCE). The best collision energies turned out to be 20, 25, 30, and 35 (a.u.). Additionally, fragmentation spectra were recorded at an NCE of 50 to compare to the AIF scans and at an optional NCE of 100.

**Table 1:** Pyoverdines defined by their peptide chain analyzed in this study. Sum formulas and masses are based on the Suc-Py derivatives according to the recommendation of Meyer et al. [15] (Unusual amino acids: OHAsp, threo-β-hydroxy-Asp; OHOrn, N^4^-hydroxy-Orn Ac(Fo)OHOrn, N^4^-acetyl-(formyl) OHOrn; cOHOrn, cyclo-OHOrn (3-amino-1-hydroxy-piperidone-2); aThr, allo-Thr) (* presence of a Thr instead of Thr reported in literature [14])

Sample ID	Peptide chain	Sum formula Suc-Py	Nom. mass Suc-Py (Da)	[*M* + H]^+^ Suc-Py (*m/z*)	[*M* + 2H]^2+^ Suc-Py (*m/z*)
Py SA	Ser-Lys-Gly-FoOHOrn-(Lys-FoOHOrn-Ser)	C_49_H_72_N_14_O_19_	1160	1161.51764	581.26273
PAO1	Ser-Arg-Ser-FoOHOrn-(Lys-FoOHOrn-Thr-Thr)	C_55_H_83_N_17_O_22_	1333	1334.59768	667.80275
1–60	Ser-FoOHOrn-Orn-Gly-aThr*-Ser-cOHOrn	C_45_H_65_N_13_O_19_	1091	1092.45979	546.73381
206–12	(Ser-Dab)-FoOHOrn-Gln-Gln-FoOHOrn-Gly	C_48_H_67_N_15_O_20_	1173	1174.47650	587.74216
3A06	Asp-ε-Lys-OHAsp-Ser-Thr-Ser-Lys-cOHOrn	C_52_H_76_N_14_O_23_	1264	1265.52860	633.26821
3B19	Asp-FoOHOrn-Lys-(Thr-Ala-Ala-Lys-FoOHOrn-Lys)	C_61_H_93_N_17_O_22_	1415	1416.67593	708.84188
3C16	Asp-Ala-Asp-AcOHOrn-Ser-cOHOrn	C_44_H_60_N_11_O_20_	1047	1048.38596	524.69689
3D19	Ala-AcOHOrn-Gly-Gly-Ser-Ala-OHAsp-Thr	C_43_H_57_N_12_O_22_	1122	1123.41799	562.21291
3F12	Lys-AcOHOrn-Gly-Thr-Thr-Gln-Gly-Ser-cOHOrn	C_55_H_82_N_16_O_22_	1318	1319.58678	660.29730
3G07	Asp-FoOHOrn-Lys-(Thr-Ala-Ala-FoOHOrn-Lys)	C_55_H_81_N_15_O_21_	1287	1288.58097	644.79440
S3a05	Lys-AcOHOrn-Ala-Gly-aThr*-Ser-cOHOrn	C_47_H_69_N_13_O_18_	1103	1104.49618	552.75200
S3a20	ɛ-Lys-OHAsp-Ala-aThr*-Ala-cOHOrn	C_42_H_59_N_11_O_17_	989	990.41686	495.71234
S3b09	Asp-ε-Lys-OHAsp-Ser-aThr*-Ala-Thr-Lys-cOHOrn	C_56_H_83_N_15_O_24_	1349	1350.58136	675.79459
S3b16	Ala-Lys-Thr-Ser-AcOHOrn-cOHOrn	C_45_H_66_N_12_O_17_	1046	1047.47471	524.24127
S3c13	Ser-Val-OHAsp-Gly-Thr-Ser-cOHOrn	C_43_H_59_N_11_O_20_	1049	1050.40161	525.70472
S3e20	Ser–Lys–Ala–Ser–Ser–AcOHOrn–Ser–Ser–cOHOrn	C_53_H_79_N_15_O_23_	1293	1294.55515	647.78149
S3g01	Ala-AcOHOrn-Ala-Gly-Ser-Ala-OHAsp-Thr	C_46_H_64_N_12_O_22_	1136	1137.43364	569.22073

An overview of the fragmentation behavior of pyoverdine PAO1, 206–12, 1–60, and 3G07 can be found in the electronic supplementary (Fig. S1–S2). For PAO1, a good fragmentation was achieved at an NCE of 35. Its Suc-Py consists of 8 amino acids of which the last four cyclize, forming a new amide bond. This seems to lead to a stabilizing effect. A similar observation was made for the Suc-Py of Py SA, also belonging to the class of cyclopeptides. Pyoverdines containing a C-terminal cyclo-hydroxyornithine such as Suc-Py from sample 1–60 started to fragment already at an NCE of 25. Even though the precursor was mostly intact, the highest diversity of fragments was present at this point. Using a higher collision energy produced solely more low *m/z* fragments containing little to no structural information. Comparing the Suc-Py of 3G07 and 206–12, the difference in fragmentation of a cyclo-depsipeptide (presence of an ester bond) and a peptide ending with a free COOH group was observable. While the cyclo-depsipeptide was stable until 30 NCE, the uncyclized 206–12 pyoverdine fragmented at 25 NCE. This suggests again an increase of stability by cyclization.

Hence, multiple collision energies are necessary for a general applicable method. As cyclized pyoverdines tend to be more stable, the first structural information can be gained simply by determining the optimal collision energy. Recording additional MS/MS spectra at 50 and 100 NCE allows to measure the pyoverdine characteristic fragment at *m/z* 204 in large abundance.

As literature reports several cases of re-arrangement reactions during the fragmentation of multiply charged species [22], the [*M* + H]^+^ ions were also chosen as a precursor, if present. In our case, all MS/MS spectra of the [*M* + 2H]^2+^ ions could be easily evaluated. Nevertheless, MS/MS parameters were set in such a way that our method can also cover the fragmentation of the [*M* + H]^+^ ion during the same measurement run. Using a loop count of 12, two coeluting precursor ions can be picked and fragmented at six different isolated NCEs (20, 25, 30, 35, 50, and 100) delivering at least 8 scans over the peak per collision energy. The resolution of 35,000, the long injection time, and AGC target improve the sensitivity compared to lower resolution settings. In this way, high-quality MS/MS spectra are obtained in a single run. 

**Fig. 8:**
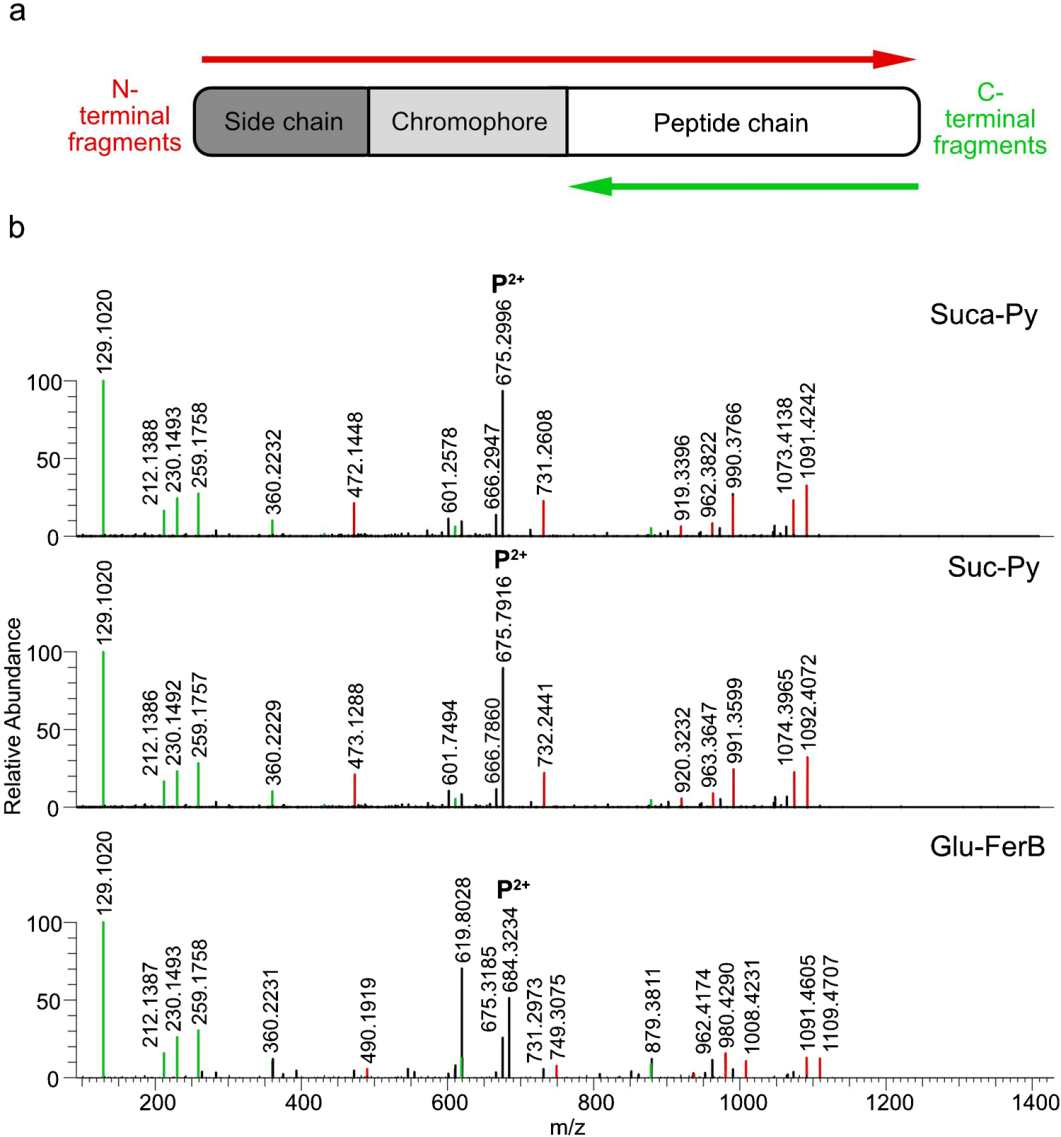
**a** Visualization of N- and C-terminal fragments for pyoverdine derivatives (variations of the side chain and/or chromophore). **b** Exemplary MS/MS spectra of Suca-Py, Suc-Py, and Glu-FerB at NCE 25 found in the bacterial extract of S3b09 (green = C-terminal fragments with identical m/z value, red = N-terminal fragments with a mass shift due to the side chain and/or chromophore variation)

An elegant way to overcome various problems during the annotation of fragments is to record MS/MS spectra of at least two pyoverdine derivatives produced by a single bacterial strain instead of choosing only one precursor ion. The advantage of this procedure is illustrated in Fig. 8 where the fragmentation patterns of Glu-FerB, Suca-Py and Suc-Py of S3b09 are compared: As the peptide chain is identical for all pyoverdine derivatives, fragments that were broken off from the C-terminus and that do not contain the chromophore together with its side chain (X, Y, and Z fragments) own the same *m/z* signals in all fragmentation spectra (highlighted in green). In contrast to this, fragments including the chromophore/side chain unit (A, B, and C fragments) will result in different masses. This mass difference accounts *m/z* − 0.9840 and *m/z* + 17.0629 when comparing Suc-Py to Suca-Py and Glu-FerB, respectively (highlighted in red). As the side chain has little influence on the fragmentation behavior, similar fragment ion intensities are observed for all three compounds at the same collision energy. Thus, a comparison is easily possible.

### Assembling block by block: structural reconstruction

**Fig. 9:**
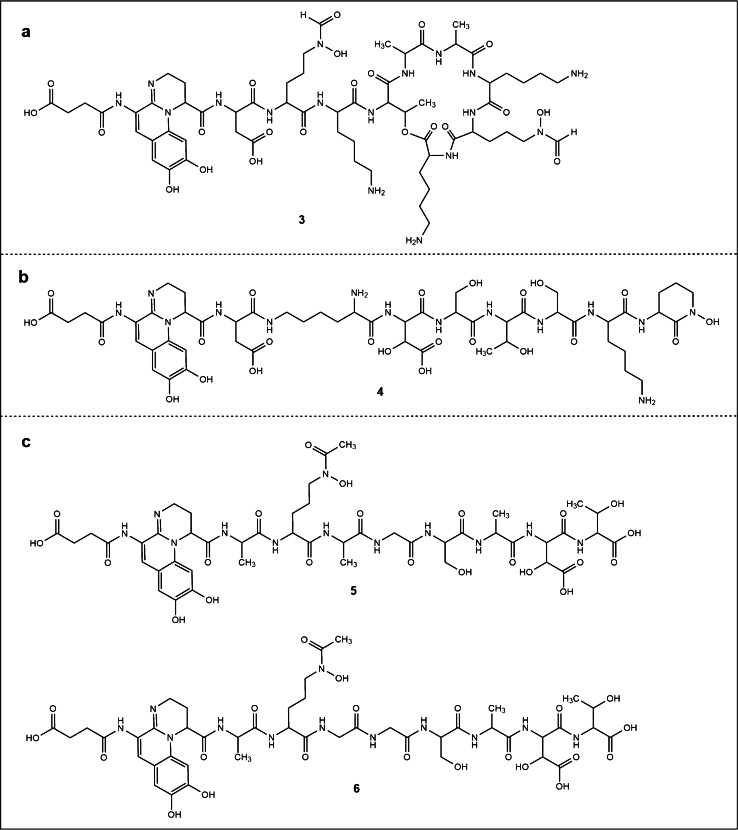
**a** Novel pyoverdine with a cyclo-depsipeptide chain found in sample 3B19 (**3**). **b** Novel pyoverdine ending with a cyclo-hydroxyornithine found in sample 3A06 (**4**). **c** Novel pyoverdines with a free ending C-terminal peptide chain found in sample S3g01 (**5**) and 3D19 (**6**)

The optimized fragmentation method was applied to all bacterial samples and the fragmentation interpretation pipeline was validated using the reference compounds of PAO1, 206–12, 1–60, and Py SA. Final elucidated peptide sequences are listed in Table 1. An existing reference [14] was found for all but four peptide chains. Thus, 3A06, 3D19, S3g01, and 3B19 contain newly discovered pyoverdines with various C-terminal endings according to the best of our knowledge. Their Suc-Py structures are shown in Fig. 9 (**3**–**6**). For each sample, a fragment list containing ppm deviations and a visualized chemical structure as well as the mainly used MS/MS spectra at a specific NCE of the corresponding Suc-Py are attached in the supplementary materials (Tables S3–S19).

In all cases, the Glu-FerB precursor was successfully identified and led to the determination of the Suc-Py and Suca-Py masses. For each sample, an ideal NCE value was found where sufficient fragments were present proving that the stepped collision values from 20 to 35 (a.u.) cover an ideal range. Thereby, linear pyoverdines tended to fragment at 20 and 25 NCE while cyclized structures required a higher NCE of 30 or 35.

Adapting the nomenclature for peptide fragmentations, B and Y″ ions are reported to be the most abundant ions in pyoverdine MS/MS fragmentation. As the A_1_ or B_1_ fragment are the easiest distinguishable signals, the reconstruction of the complete p eptide sequence is possible [15]. A short overview over the most expected A_1_ and B_1_ fragments based on the first amino acid in the peptide sequence is given in Table 2.

**Table 2:** Expected MS/MS fragments depending on the side chain and the first amino acid residue as reported by Budzikiewicz et al. [15]

1^st^ amino acid of the pyoverdine peptide sequence	Succinic amide	Succinic acid
Ser	416.1565 (A_1_)	417.1405 (A_1_)
Ala	400.1615 (A_1_)	401.1456 (A_1_)
ε-Lys	457.2194 (A_1_)	458.2034 (A_1_)
Lys	485.2143 (B_1_)	486.1983 (B_1_)
Asp	472.1463 (B_1_)	473.1303 (B_1_)

After the identification of the first A or B fragments, the pyoverdine peptide chain is subsequently elucidated by screening for the next fragment ions. Referring to the publication of Ye et al. [21], certain probabilities exist on the order of amino acids in a sequence. By using this information, the most likely occurring amino acids can be checked first. For lysine, occasionally a linkage of the ε- instead of the α-amino group to its preceding carboxyl group is observed. If an absence or very low intensity of the corresponding B fragment is noted, a valuable indication for an ε-linkage is given [29]. As all measurements were performed on a high-resolution mass spectrometer with a high mass precision, all measured masses including fragments should be compared to a calculated theoretical value as an additional support. As pyoverdines are natural products, only a limited set of amino acids are available to explain certain mass differences in the fragmentation spectra. Critical cases are Asn/Orn and Gln/Lys that possess the same nominal mass. However, their monoisotopic masses differ over 20 ppm. Hence, a tolerated error window of 5 ppm was chosen that is small enough to differentiate closely related amino acids but is also large enough to account for small intensity signals or potential matrix effects. Last but not least, the interpretation did not rely on the presence of characteristic fragments of certain amino acids, as these are reported to be sometimes missing [15].

The structure elucidation method was firstly applied to four pyoverdine references with already established structures. PAO1 and the Sigma Aldrich standard (Py SA) were chosen as both are cyclopeptides that are especially challenging to elucidate [30]. Additionally, pyoverdine 1–60 and 206–12 were employed ending in a C-terminal cyclo-hydroxyornithine and a free COOH group, respectively. Hence, structural varieties were covered that might show different fragmentation behavior. The [*M* + 2H]^2+^ signal of the Suc-Py was used as the main source for structure elucidation as it was present in all samples.

An intense fragment at *m/z* 417.1405 (A_1_) served as a starting point as serine is the first building block in all reference compounds followed by the continuous assembly of further A and B ions. Fragmentation of Suc-Py of PAO1 and Py SA resulted in more complex MS/MS spectra. Their peptide ring is opened during the fragmentation process in the HCD cell. Thereby, not only the ε-amide bond of the incorporated Lys group can be broken (that contributed to the ring closure) but the α-amide bond, too. This results in B fragments that do not follow the usual order of the peptide sequence (fragment E, see Table S3–S4). For 206–12, initially, no B_6_ fragment was found leaving the two last amino acids unclear. However, a [B_6_ + H]^2+^ was detected recognizable by the differing decimal places of the *m/z* signal completing the structure. As Suc-Py 206–12 owns a Ser followed by a Dab group, a [*M* + 2H]^2+^ signal was recorded for a Dab condensed and a Dab uncondensed form. Both ions were separately chosen as a precursor for fragmentation and compared. A cleaner MS/MS spectrum was obtained for the uncondensed species making it the better choice for structure elucidation. MS/MS of Suc-Py from 1–60 gave a high-intensity signal at *m/z* 131.0814. This fragment belongs to a characteristic Y″_1_ ion that consists of terminal cyclo-hydroxyornithines. Taking this fragment into account, the effort to characterize an unfamiliar peptide can be reduced. In the end, all reference structures were correctly elucidated ab inito, proving that the optimized fragmentation and interpretation procedure works/is valid.

All unknown samples could be confidently analyzed. The starting point of the fragment interpretation (A_1_ or B_1_) was effortlessly detected in all cases. Similar to the reference compounds, Suc-Py from S3c13 and S3e20 started with a serine group and gave an intense signal at *m/z* 417.1405 (A_1_). On the other hand, the Suc-Py produced by 3A06, 3G07, 3B19, and S3b09 began with an aspartic acid (B_1_: *m/z* 473.1303). Suc-Py starting with an alanine from sample 3D19, S3b16, and S3g01 resulted in a clear fragment at *m/z* 401.1456 (A_1_). For the characterization of ε-Lys vs. Lys components, the lack of the corresponding B frag ment was the deciding factor for an ε-linkage as observed for 3A06, S3b09, and S3a20. In these cases, only a rather small signal for the A fragment was detected. For S3a05, 3B19, and al l remaining compounds, a clear B fragment incorporating a Lys was found proving the usual amino acid connectivity.

The pyoverdine of 3G07 and 3B19 turned out to be cyclo-depsipeptides owning a ring system caused by a condensation of a threonine with the terminal lysine. Despite their cyclic structure, all A and B fragments necessary for their elucidation were easily located. Since the ester bond is readily hydrolyzed and broken, less fragments with an intact ring are observed. In contrast, amide bonds are more stable due to their double bond character that requires higher collision energies for their breakage. Thus, more complicated spectra are obtained when fragmenting cyclo-peptides.

**Fig. 10:**
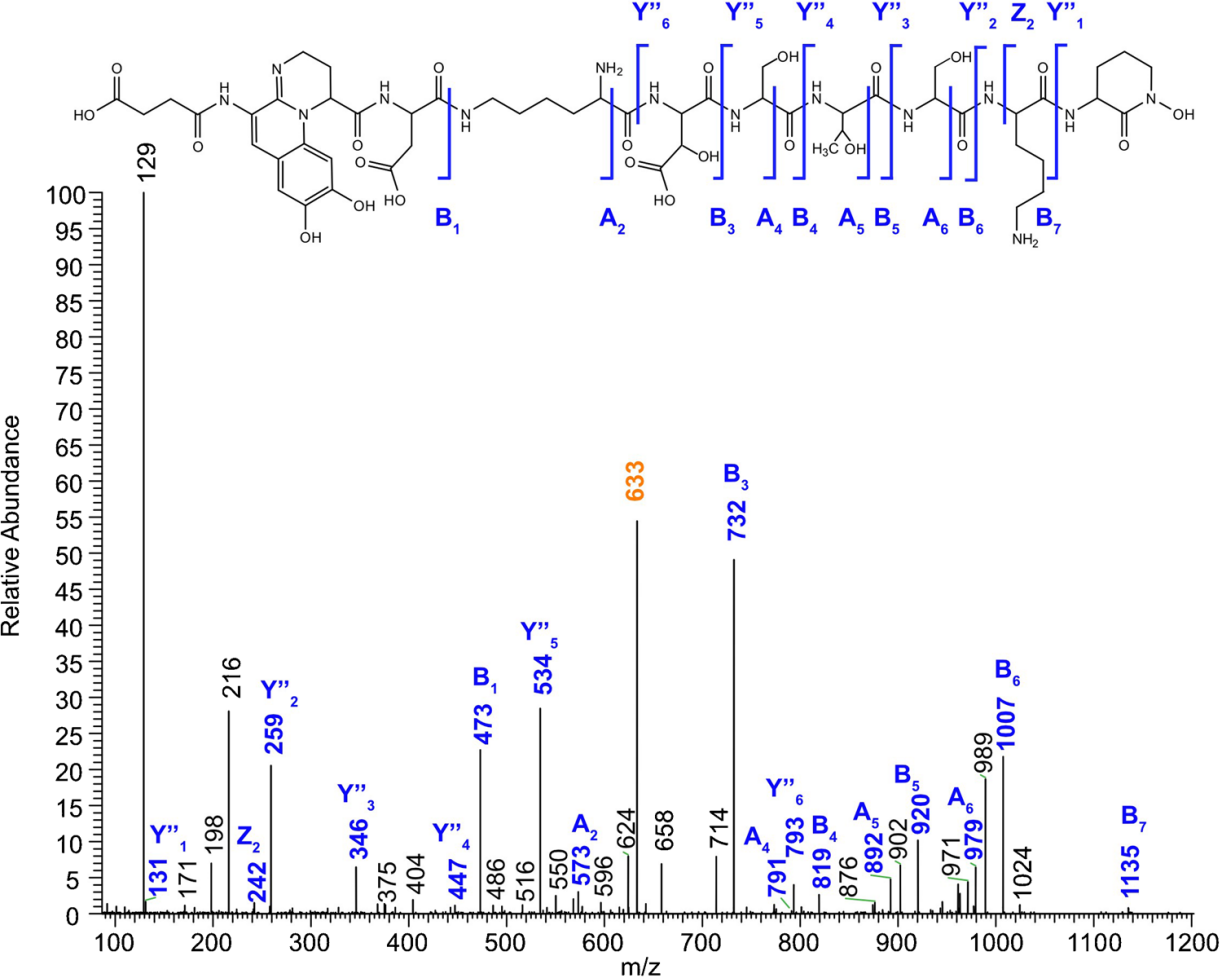
MS/MS spectrum of the Suc-Py [*M* + 2H]^2+^ ions (*m/z* 633.26821) of 3A06 (NCE = 25)

Afterwards, the following A and/or B fragments could be determined. By comparison of at least two MS/MS spectra of different pyoverdine derivatives, the nature of fragment ions, C- or N-terminal, was readily pinpointed. One example of a completely annotate MS/MS spectrum is presented in Fig. 10 and Table 3 for the new compound **4** found in 3A06.

**Table 3:** Identified fragments in the MS/MS spectrum of the Suc-Py [*M* + 2H]^2+^ ions (*m/z* 633.26821) found in extract 3A06 at NCE 25. Ppm deviations are calculated based on the difference between theoretical and measured masses. *Theoretical masses were calculated using the mass and fragment predictor for pyoverdines and its derivatives

	Formula	Theoretical mass* (*m/z*)	Measured mass (*m/z*)	Deviation (ppm)
[*M* + 2H]^2+^	C_52_H_78_N_14_O_23_	633.26821	633.26699	− 1.93
A_2_	C_26_H_33_N_6_O_9_	573.23035	573.23188	2.66
A_4_	C_33_H_43_N_8_O_15_	791.28424	791.28388	− 0.45
A_5_	C_37_H_50_N_9_O_17_	892.33192	892.33267	0.84
A_6_	C_40_H_55_N_10_O_19_	979.36395	979.36458	0.65
B_1_	C_21_H_21_N_4_O_9_	473.13030	473.13006	− 0.52
B_3_	C_31_H_38_N_7_O_14_	732.24713	732.24671	− 0.57
B_4_	C_34_H_43_N_8_O_16_	819.27915	819.27946	0.37
B_5_	C_38_H_50_N_9_O_18_	920.32683	920.32572	− 1.21
B_6_	C_41_H_55_N_10_O_20_	1007.35886	1007.35886	− 0.00
B_7_	C_47_H_67_N_12_O_21_	1135.45382	1135.45068	− 2.77
Y’’_1_	C_5_H_11_N_2_O_2_	131.08150	131.08171	1.57
Y’’_2_	C_11_H_23_N_4_O_3_	259.17647	259.17652	0.20
Y’’_3_	C_14_H_28_N_5_O_5_	346.20850	346.20850	0.00
Y’’_4_	C_18_H_35_N_6_O_7_	447.25617	447.25742	2.79
Y’’_5_	C_21_H_40_N_7_O_9_	534.28820	534.28826	0.11
Y’’_7_	C_31_H_57_N_10_O_14_	793.40502	793.40428	− 0.94
Z_2_	C_11_H_20_N_3_O_3_	242.14992	242.14994	0.09

By using accurate high-resolution MS, the ppm error to theoretical values was finally calculated. In all our cases, this error was below 5 ppm.

To sum up, the structure of all pyoverdine samples was successfully elucidated, thanks to the well-specified methodology used in the measurement and interpretation of the MS/MS spectra. 3A06, 3D19, S3g01, and 3B19 owned new peptide sequences not reported previously resulting in a considerable extension of the currently known pyoverdine structure list [14].

### Mass calculator and fragment predictor for pyoverdines and derivatives

While rapid MS-based protein sequencing is made possible by automated computational algorithms, pyoverdine structure elucidations were conducted mainly by hand until today. Whereas this might be feasible for few samples, a manual characterization is tedious and takes many hours for multiple compounds. Furthermore, pyoverdine masses are typically reported from the corresponding Suc-Py molecule without decimal numbers, which is insufficient for high-resolution mass spectrometry. Also, no sum formulas are usually indicated and only the peptide chain sequence is stated [14]. Hence, a pyoverdine mass calc ulator using easily editable drop-down menus was developed in Excel (the spreadsheet is available in the supplementary materials). Exact masses of atoms were taken from IUPAC [31]. Based on the publications of Budzikiewicz et al. [15], the following aspects were taken into account: the variability of the side chain, the chromophore, the peptide sequence, and the peptide end. All to date published partial structures were considered and made available for selection. Thus, the monoisotopic mass of all pyoverdine derivates can be predicted together with their [*M* + H]^+^ and [*M* + 2H]^2+^ *m/z* values.

Furthermore, a mass difference table was created to faster scrutinize the full scan for possible pyoverdines and other derivatives. If one has the accurate mass for the ferribactin precursor, the Excel file can predict quickly the [*M* + 2H]^2+^ *m/z* values of all other theoretically possible combinations of side chain and chromophore. Thus, additional time can be saved when looking for the masses of Suc-Py or Suca-Py.

Last but not least, a fragmentation predictor for A, B, C, X, Y, Y″, and Z ions was created. This is of massive advantage when interpreting fragmentation spectra as decimal numbers can be immediately compared from predicted to measured values such as presented in Table 3. Even though it is designed for linear peptides, it partially works for cyclized compounds, too, especially when taking a water loss from the cyclization reaction into account. Electrons were minded for accurate masses.

All in all, the pyoverdine calculator produces not only accurate monoisotopic masses but also supports fragmentation interpretation. The calculator and fragmentation predictor were tested and verified with the known pyoverdine structures and proved to be working smoothly.

## Conclusion

Siderophores gained a large interest in research due to their pathogen control properties making systematic structure elucidation methods at high-throughput desirable. With this study, a generalized workflow was established using pyoverdine of fluorescent *Pseudomonas* spp. as a model siderophore. This workflow was validated using 17 different pyoverdine extracts. Sample preparation takes place by a small-scale SPE saving time and resources to obtain pure solutions for analysis. The herein reported UHPLC conditions deliver an excellent separation in only 15 min using reserved phase column chemistry. The process to identify pyoverdines was simplified by the search for the ferribactin fragment in an AIF scan and is of great advantage when analyzing unusual *Pseudomonas* species that produce atypical pyoverdine derivatives such as isopyoverdine. Our targeted MS/MS experiments using multiple collision energies can be applied to a broad range of pyoverdines showing various fragmentation behavior resulting in high-quality MS/MS spectra. Finally, the lack of a mass calculator and fragmentation predictor for pyoverdine and its derivatives was addressed. Here, we make a simple but efficient Excel tool available that speeds up the work-intensive comparison of measured and theoretical masses that is necessary when working with high-resolution mass spectrometry. This analytical approach can serve as a basis for the development of high-throughput methods to characterize other siderophore classes and secondary metabolites from small sample volumes, thereby accelerating research progress.

## Supplementary Information

Below is the link to the electronic supplementary material.

Supplementary file1 (XLSX 70 KB)

Supplementary file2 (PDF 2708 KB)

## Data Availability

Fully described methods are provided using commercially available materials. Origin of bacterial cultures is fully disclosed. For the MS/MS fragmentation, exact measured masses as well as the raw MS/MS spectra are given.
