# Supplementary material for: A comprehensive method to elucidate pyoverdines produced by fluorescent Pseudomonas spp. by UHPLC-HR-MS/MS
Source: Anal Bioanal Chem. 2022 Jan 27;414(8):2671–85. doi: 10.1007/s00216-022-03907-w (PMC8888394; doi:10.1007/s00216-022-03907-w)

Digital Supplementary

Sample ID	Sum Formula	Targeted Ion $[M+2H]^{2+}$ (m/z)	Charge	Optimal NCE
Py SA	$C_{49}H_{72}N_{14}O_{19}$	581.26273	2	30
PAO1	$C_{55}H_{83}N_{17}O_{22}$	667.80275	2	35
1-60	$C_{45}H_{65}N_{13}O_{19}$	546.73381	2	25
206-12	$C_{48}H_{67}N_{15}O_{20}$	587.74216	2	25
3A06	$C_{52}H_{76}N_{14}O_{23}$	633.26821	2	25
3B19	$C_{61}H_{93}N_{17}O_{22}$	708.84188	2	30
3C16	$C_{44}H_{60}N_{12}O_{20}$	539.21017	2	25
3D19	$C_{43}H_{57}N_{12}O_{22}$	562.21291	2	25
3F12	$C_{55}H_{82}N_{16}O_{22}$	660.29730	2	30
3G07	$C_{55}H_{81}N_{15}O_{21}$	644.79440	2	30
S3a05	$C_{47}H_{69}N_{13}O_{18}$	552.75200	2	25
S3a20	$C_{42}H_{59}N_{11}O_{17}$	495.71234	2	20
S3b09	$C_{56}H_{83}N_{15}O_{24}$	675.79459	2	25
S3b16	$C_{45}H_{66}N_{12}O_{17}$	524.24127	2	30
S3c13	$C_{43}H_{59}N_{11}O_{20}$	525.70472	2	25
S3e20	$C_{53}H_{79}N_{15}O_{23}$	647.78149	2	25
S3g01	$C_{46}H_{64}N_{12}O_{22}$	569.22073	2	25

Table S1. Targeted ions and NCE values for MS/MS analysis used for pyoverdine structure elucidation.

Sample ID	RT (min) of			
	Glu-FerB	Suc-Py	Suca-Py	Glu-IsoPy
Py SA	-	3.28	2.63	-
PAO1	3.16	3.97	3.36	-
1-60	1.73	2.58	1.86	-
206-12	1.45	2.42	-	-
3A06	1.87	2.98	2.33	-
3B19	1.37	2.09	1.41	-
3C16	4.63	-	-	3.98
3D19	3.85	4.41	3.69	-
3F12	3.39	4.10	3.37	-
3G07	2.29	3.04	2.44	-
S3a05	3.58	4.18	3.48	-
S3a20	3.30<br>(H-FerB: 2.99)	4.27	3.55	-
S3b09	2.41	3.39	2.80	-
S3b16	3.39	3.91	3.33	-
S3c13	4.85	5.78	5.01	-
S3e20	3.08	3.84	3.21	-
S3g01	4.89	5.18	4.50	-

Table S2. Average retention times of identified pyoverdines and ferribactins found in the extracts of the investigated bacterial strains.

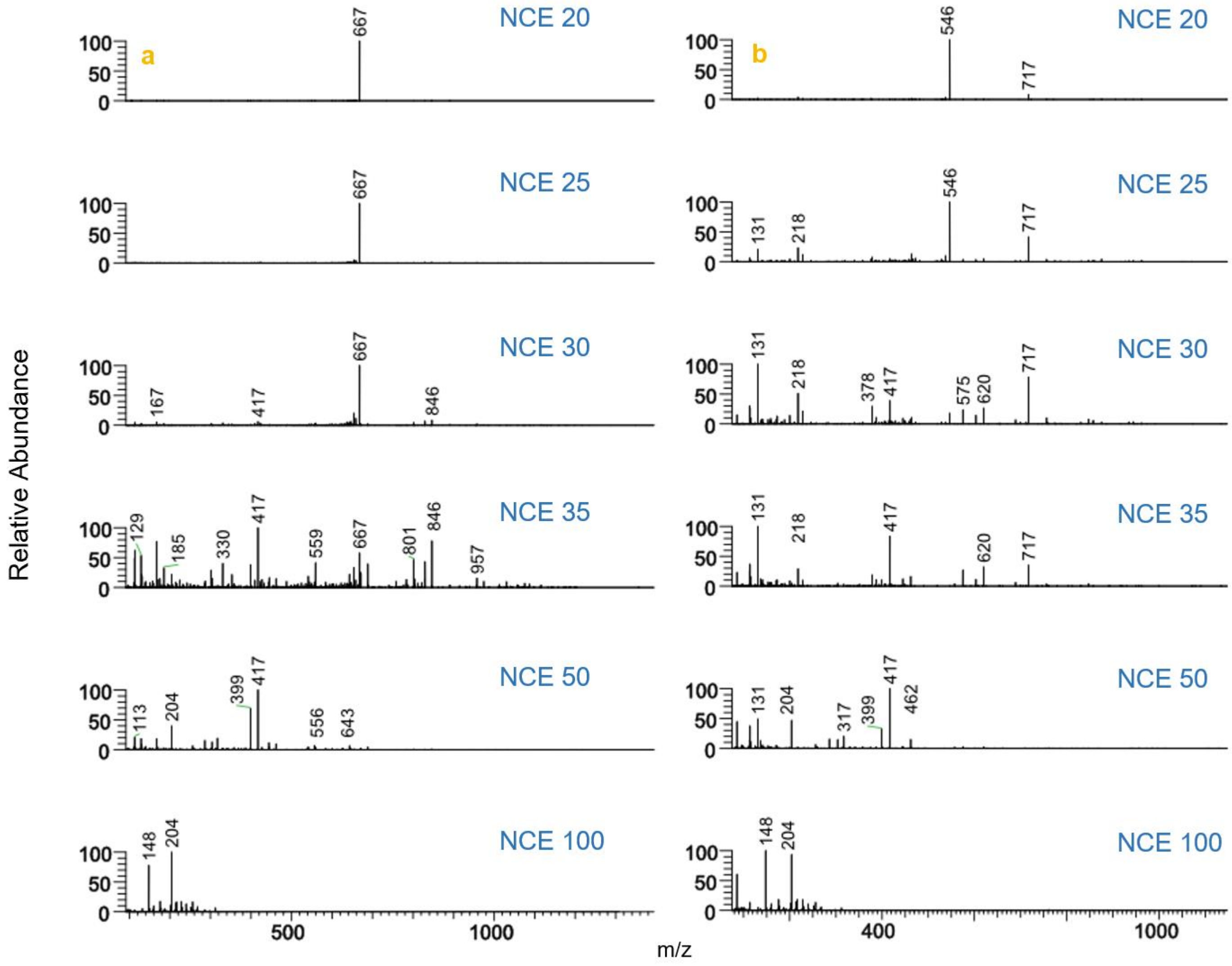

Fig. S1. Stepped MS/MS acquired of the doubly charged Suc-Py $[M+2H]^{2+}$ ions of the bacteria PAO1 (*m/z* 667.80275) (a) and 1-60 (*m/z* 546.73381) (b).

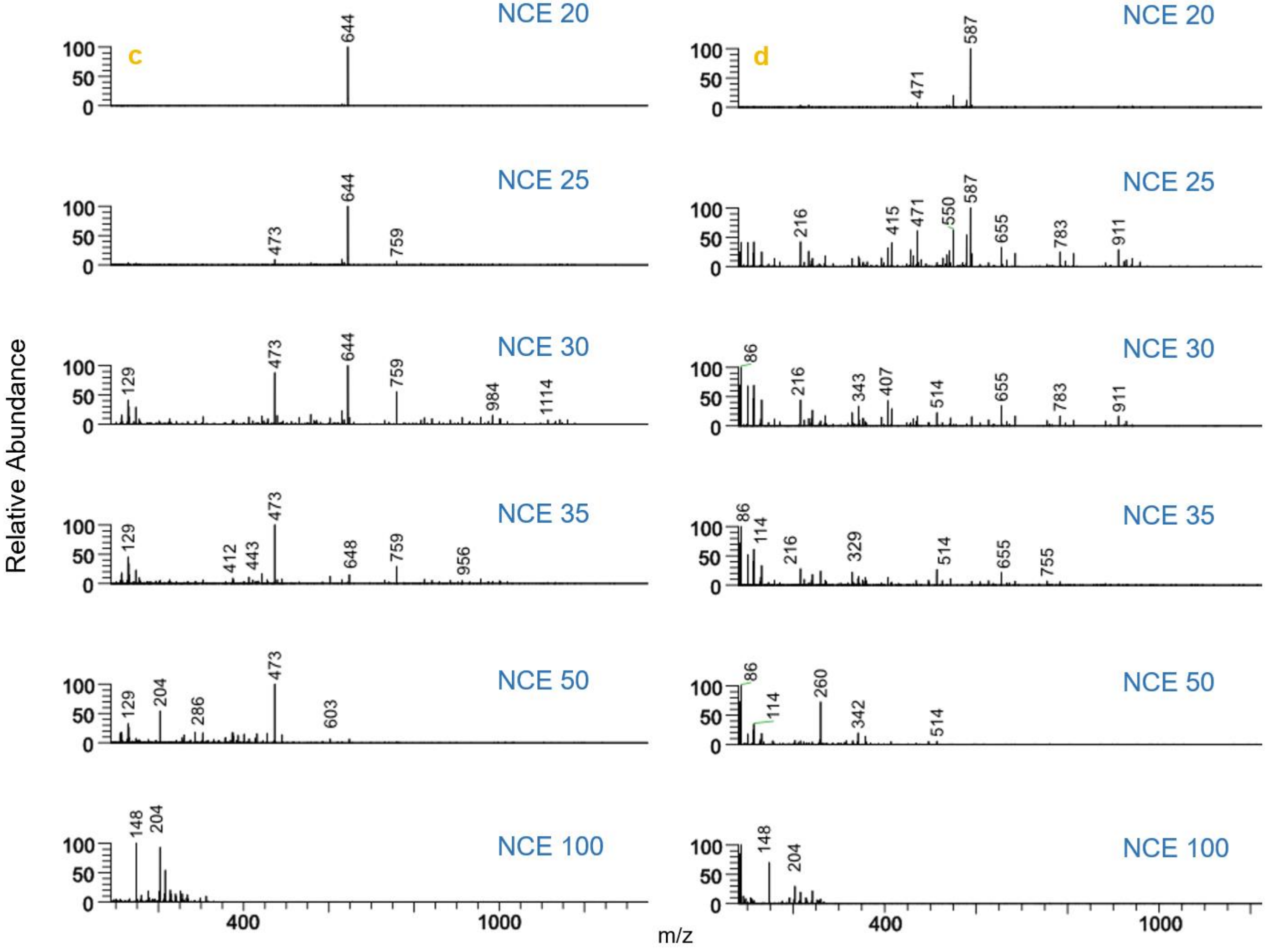

Fig. S2. Stepped MS/MS acquired of the doubly charged Suc-Py [*M*+2H]$^{2+}$ ions of the bacteria 3G07 (*m/z* 644.79440) (c) and 206-12 (*m/z* 587.74216) (d)

Table S3. Detected fragments in the MS/MS fragmentation of Suc-Py of Py SA compared to their theoretical value *m/z* value and their ppm deviation.

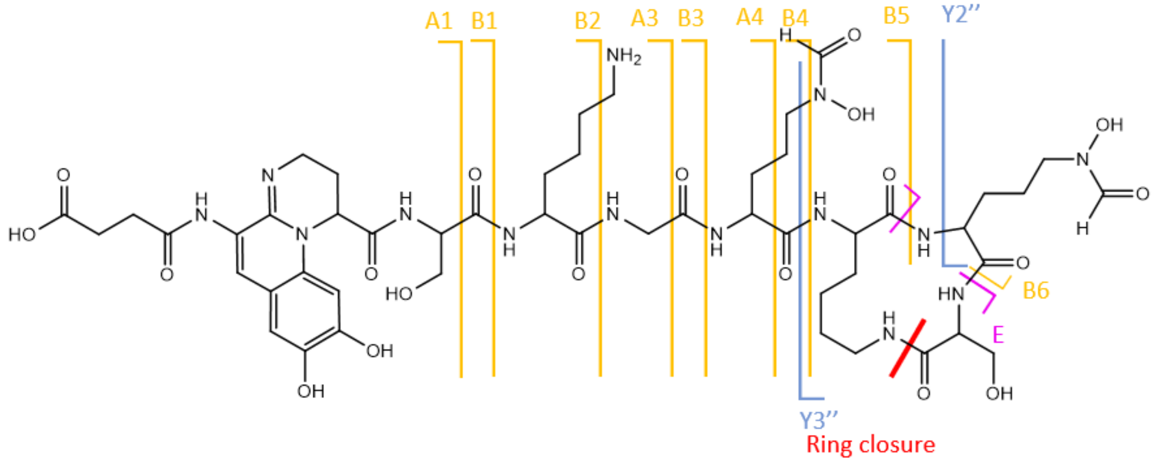

Name:	Py SA			
**Sequence:**	Ser-Lys-Gly-FoOHOrn-(Lys-FoOHOrn-Ser)			
	**Formula**	**Mass (*m/z*)**	**Found Mass (*m/z*)**	**ppm Deviation**
**$[M+H]^+$**	$C_{49}H_{73}N_{14}O_{19}$	1161.51764	1161.51704	-0.52
**$[M+2H]^{2+}$**	$C_{49}H_{74}N_{14}O_{19}$	581.26273	581.26216	-0.99
**Fragment Type (NCE = 30)**	**Formula**	**Mass (*m/z*)**	**Found Mass (*m/z*)**	**ppm Deviation**
**A_{01}**	$C_{19}H_{21}N_4O_7$	417.14048	417.14066	0.44
**A_{01} - H_2O**	$C_{19}H_{19}N_4O_6$	399.12991	399.13060	1.73
**A_{03}**	$C_{27}H_{36}N_7O_9$	602.25690	602.25637	-0.88
**A_{04}**	$C_{33}H_{46}N_9O_{12}$	760.32604	760.32613	0.11
**A_{04} - H_2O**	$C_{33}H_{44}N_9O_{11}$	742.31548	742.31585	0.50
**B_{01}**	$C_{20}H_{21}N_4O_8$	445.13539	445.13561	0.49
**B_{02}**	$C_{26}H_{33}N_6O_9$	573.23035	573.23036	0.01
**B_{03}**	$C_{28}H_{36}N_7O_{10}$	630.25182	630.25187	0.08
**B_{03} - H_2O**	$C_{28}H_{34}N_7O_9$	612.24125	612.24126	0.01
**B_{04}**	$C_{34}H_{46}N_9O_{13}$	788.32096	788.32068	-0.35
**B_{04} - H_2O**	$C_{34}H_{44}N_9O_{12}$	770.31039	770.30967	-0.94
**B_{05} + H - H_2O**	$C_{40}H_{56}N_{11}O_{13}$	898.40536	898.40605	0.77
**B_{05} + H**	$C_{40}H_{58}N_{11}O_{14}$	916.41592	916.41545	-0.52
**B_{06} + H (NCE 25)**	$C_{46}H_{68}N_{13}O_{17}$	1074.4856	1074.4856	0.01
**Y_{02} + H**	$C_9H_{16}N_3O_5$	246.10845	246.10898	2.17
**Y''_{03}**	$C_{15}H_{28}N_5O_6$	374.20341	374.20392	1.36
**E + H**	$C_{43}H_{63}N_{12}O_{16}$	1003.44795	1003.44947	1.51

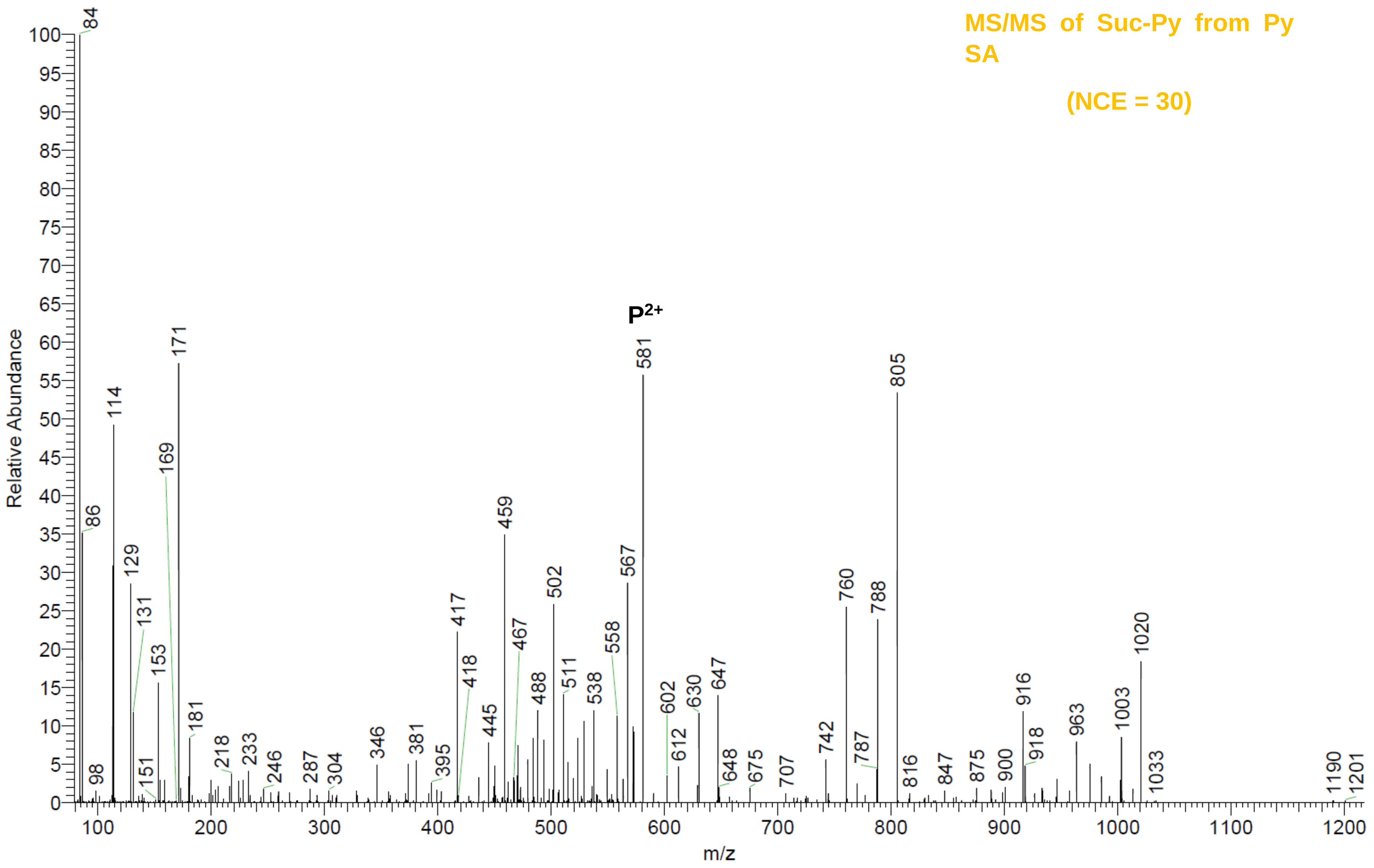

MS/MS of Suc-Py from Py SA
(NCE = 30)
P2+
Relative Abundance
m/z

Table S4. Detected fragments in the MS/MS fragmentation of Suc-Py of PAO1 compared to their theoretical value *m/z* value and their ppm deviation.

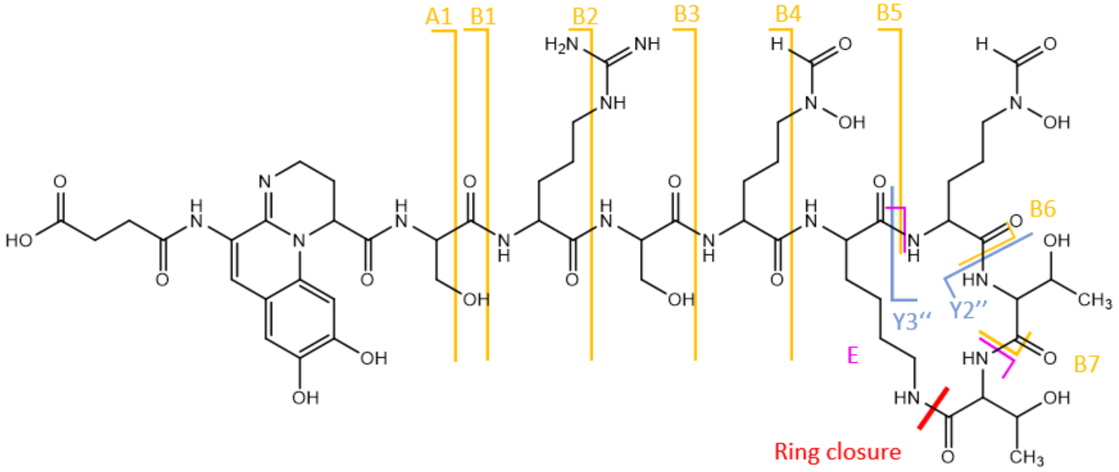

Name:	PAO1			
**Sequence:**	Ser-Arg-Ser-FoOHOrn-(Lys-FoOHOrn-Thr-Thr)			
	**Formula**	**Mass (*m/z*)**	**Found Mass (*m/z*)**	**ppm Deviation**
**[M+H]$^{+}$**	$C_{55}H_{84}N_{17}O_{22}$	1334.59768	1334.59766	-0.02
**[M+2H]$^{2+}$**	$C_{55}H_{85}N_{17}O_{22}$	667.80275	667.80200	-1.13
**Fragment Type (NCE = 35)**	**Formula**	**Mass (*m/z*)**	**Found Mass (*m/z*)**	**ppm Deviation**
**A_{01}**	$C_{19}H_{21}N_{4}O_{7}$	417.14048	417.13959	-2.12
**A_{01} - H_2O**	$C_{19}H_{19}N_{4}O_{6}$	399.12991	399.12898	-2.33
**B_{01} - H_2O**	$C_{20}H_{19}N_{4}O_{7}$	427.12483	427.12388	-2.22
**B_{02}**	$C_{26}H_{33}N_{8}O_{9}$	601.23650	601.23458	-3.20
**B_{03}**	$C_{29}H_{38}N_{9}O_{11}$	688.26853	688.26721	-1.92
**B_{03} - H_2O**	$C_{29}H_{36}N_{9}O_{10}$	670.25796	670.25632	-2.45
**B_{04}**	$C_{35}H_{48}N_{11}O_{14}$	846.33767	846.33546	-2.61
**B_{04} - H_2O**	$C_{35}H_{46}N_{11}O_{13}$	828.32711	828.32543	-2.02
**[B_{05} + 2H]$^{2+}$**	$C_{41}H_{61}N_{13}O_{15}$	487.7202	487.7186	-3.30
**B_{05} - H_2O**	$C_{41}H_{58}N_{13}O_{14}$	956.42207	956.41735	-4.94
**[B_{06} + 2H]$^{2+}$ (NCE = 30)**	$C_{47}H_{71}N_{15}O_{18}$	566.7548	566.7535	-2.25
**[B_{07} + 2H]$^{2+}$ (NCE = 30)**	$C_{51}H_{78}N_{16}O_{20}$	617.2786	617.2776	-1.70
**Y''$_{02}$**	$C_{8}H_{15}N_{2}O_{4}$	203.10263	203.10256	-0.36
**Y''$_{03}$**	$C_{14}H_{25}N_{4}O_{7}$	361.17178	361.17134	-1.21
**E + H**	$C_{45}H_{67}N_{14}O_{17}$	1075.48031	1075.48038	0.06

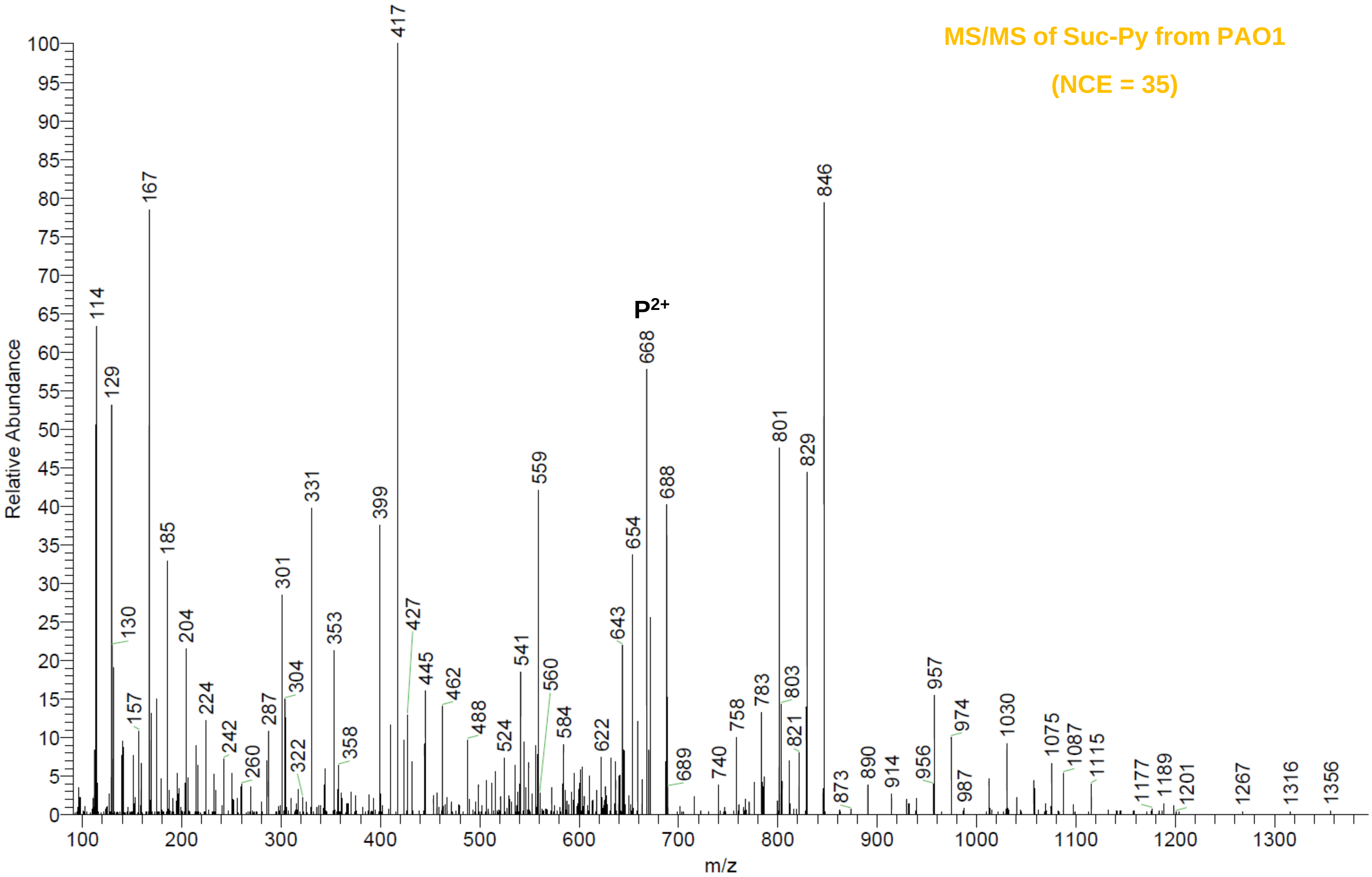
MS/MS of Suc-Py from PAO1
(NCE = 35)
Relative Abundance
m/z
P2+
114
129
130
157
167
185
204
224
242
260
287
301
304
322
331
353
358
399
417
427
445
462
488
524
541
559
560
584
622
643
654
668
688
689
740
758
783
801
803
821
829
846
873
890
914
956
957
974
987
1030
1075
1087
1115
1177
1189
1201
1267
1316
1356

Table S5. Detected fragments in the MS/MS fragmentation of Suc-Py of 206-12 compared to their theoretical value *m/z* value and their ppm deviation.

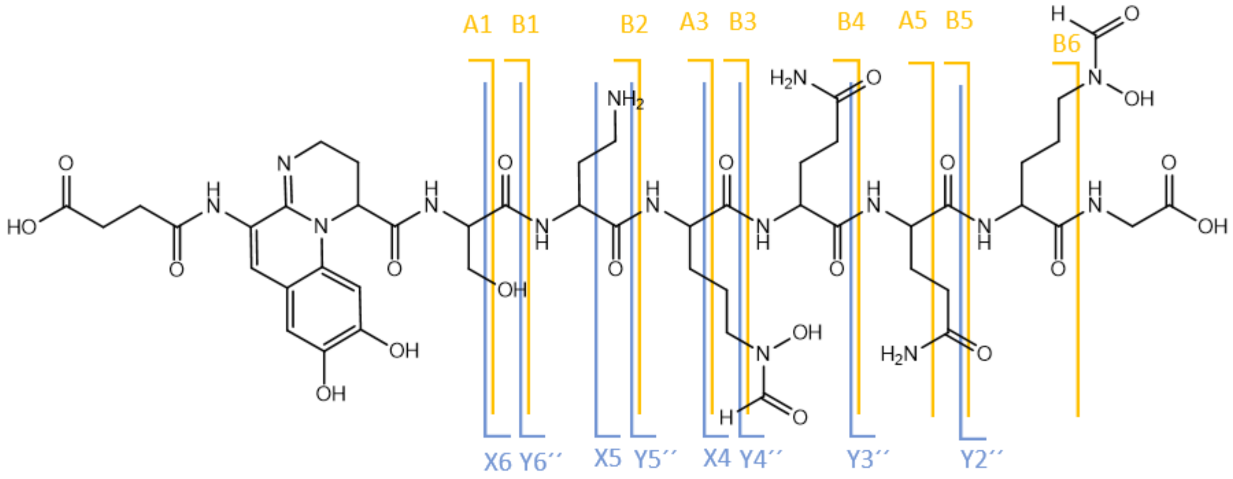

**Name:**	**206-12**			
**Sequence:**	Ser-Dab-FoOHOrn-Gln-Gln-FoOHOrn-Gly (open)			
	**Formula**	**Mass (*m/z*)**	**Found Mass (*m/z*)**	**ppm Deviation**
**[M+H]⁺**	$C_{48}H_{70}N_{15}O_{21}$	1192.48707	1192.48609	-0.82
**[M+2H]²⁺**	$C_{48}H_{71}N_{15}O_{21}$	596.74745	596.74659	-1.44
**Fragment Type (NCE = 25)**	**Formula**	**Mass (*m/z*)**	**Found Mass (*m/z*)**	**ppm Deviation**
**A_{01}**	$C_{19}H_{21}N_4O_7$	417.14048	417.13961	-2.07
**A_{03}**	$C_{29}H_{39}N_8O_{11}$	675.27328	675.27226	-1.51
**A_{05}**	$C_{39}H_{55}N_{12}O_{15}$	931.39044	931.38649	-4.23
**B_{01}**	$C_{20}H_{21}N_4O_8$	445.13539	445.13507	-0.72
**B_{02}**	$C_{24}H_{29}N_6O_9$	545.19905	545.19879	-0.48
**B_{03}**	$C_{30}H_{39}N_8O_{12}$	703.26820	703.26854	0.49
**B_{04}**	$C_{35}H_{47}N_{10}O_{14}$	831.32677	831.32495	-2.19
**B_{05}**	$C_{40}H_{55}N_{12}O_{16}$	959.38535	959.38145	-4.07
**$[B_{06} + H]^{2+}$**	$C_{46}H_{66}N_{14}O_{19}$	559.2312	559.2298	-2.50
**Y''_{03}**	$C_8H_{16}N_3O_5$	234.10845	234.10844	-0.03
**Y''_{04}**	$C_{13}H_{24}N_5O_7$	362.16702	362.16641	-1.70
**Y''_{05}**	$C_{18}H_{32}N_7O_9$	490.22560	490.22611	1.04
**Y''_{06}**	$C_{24}H_{42}N_9O_{12}$	648.29474	648.29477	0.04
**Z_{04}**	$C_{13}H_{21}N_4O_7$	345.14048	345.14057	0.27
**Z_{05}**	$C_{18}H_{29}N_6O_9$	473.19905	473.19892	-0.28
**Z_{06}**	$C_{24}H_{39}N_8O_{12}$	631.26820	631.26702	-1.86

**Additional Data:**				
**Sequence:**	(Ser-Dab)-FoOHOrn-Gln-Gln-FoOHOrn-Gly (condensed)			
	**Formula**	**Mass (*m/z*)**	**Found Mass (*m/z*)**	**ppm Deviation**
**[M+H]$^+$**	$C_{48}H_{68}N_{15}O_{20}$	1174.47650	1174.47598	-0.45
**[M+2H]$^{2+}$**	$C_{48}H_{69}N_{15}O_{20}$	587.74216	587.74134	-1.40
**Fragment Type (NCE = 25)**	**Formula**	**Mass (*m/z*)**	**Found Mass (*m/z*)**	**ppm Deviation**
**B_{03}**	$C_{30}H_{37}N_8O_{11}$	685.25763	685.25654	-1.59
**B_{04}**	$C_{35}H_{45}N_{10}O_{13}$	813.31621	813.31532	-1.09
**B_{05}**	$C_{40}H_{53}N_{12}O_{15}$	941.37479	941.37316	-1.73
**[B_{06} + H]$^{2+}$**	$C_{46}H_{64}N_{14}O_{18}$	550.2259	550.2247	-2.13
**Y''$_{03}$**	$C_8H_{14}N_3O_4$	216.09788	216.09797	0.41
**Y''$_{04}$**	$C_{13}H_{22}N_5O_6$	344.15646	344.15670	0.70
**Y''$_{05}$**	$C_{18}H_{30}N_7O_8$	472.21504	472.21503	-0.02

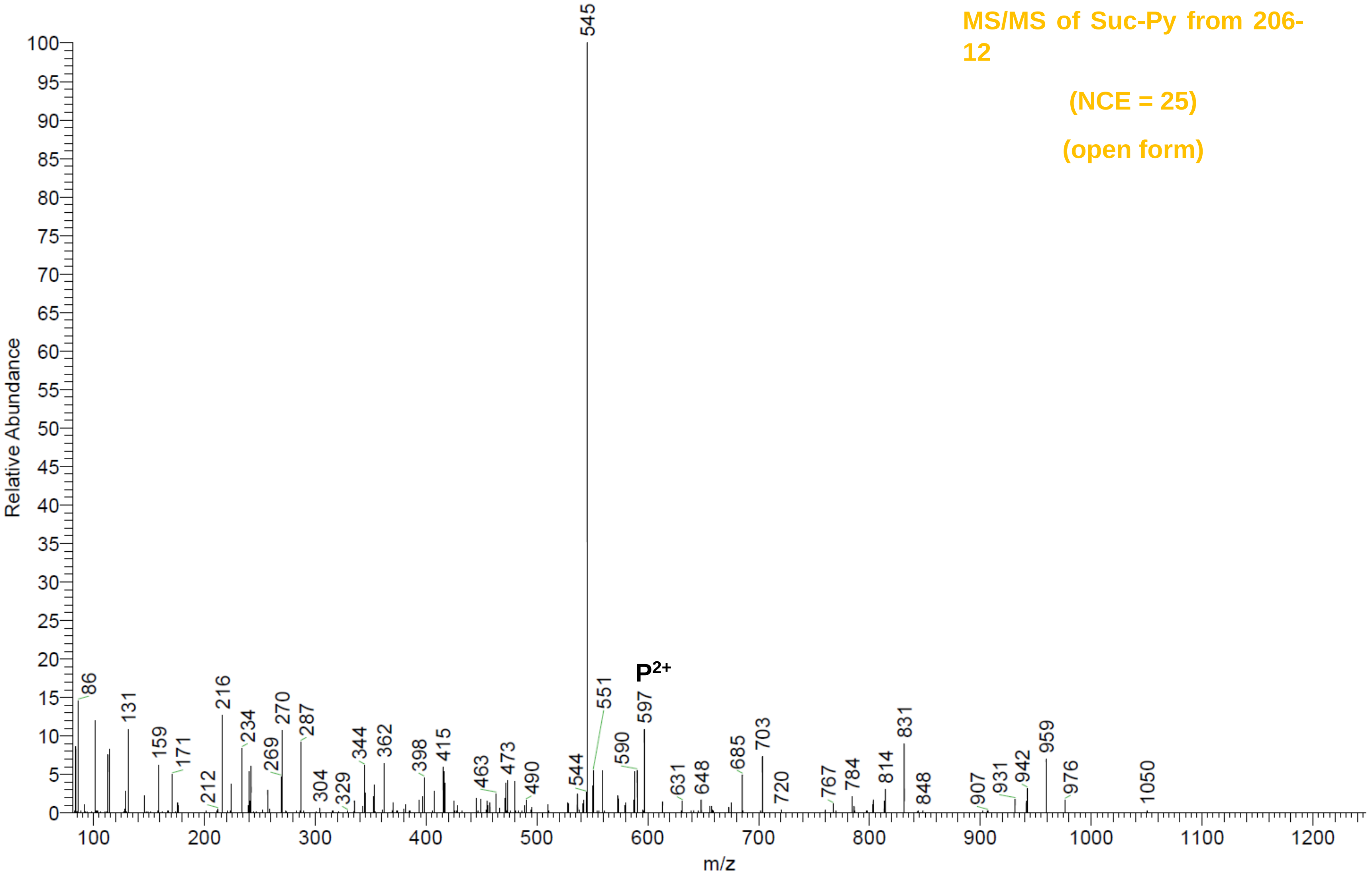
MS/MS of Suc-Py from 206-12
(NCE = 25)
(open form)
Relative Abundance
m/z
P2+
545
551
597
86
131
216
270
287
234
159
171
212
269
304
329
344
362
398
415
463
473
490
544
590
631
648
685
703
720
767
784
814
831
848
907
931
942
959
976
1050

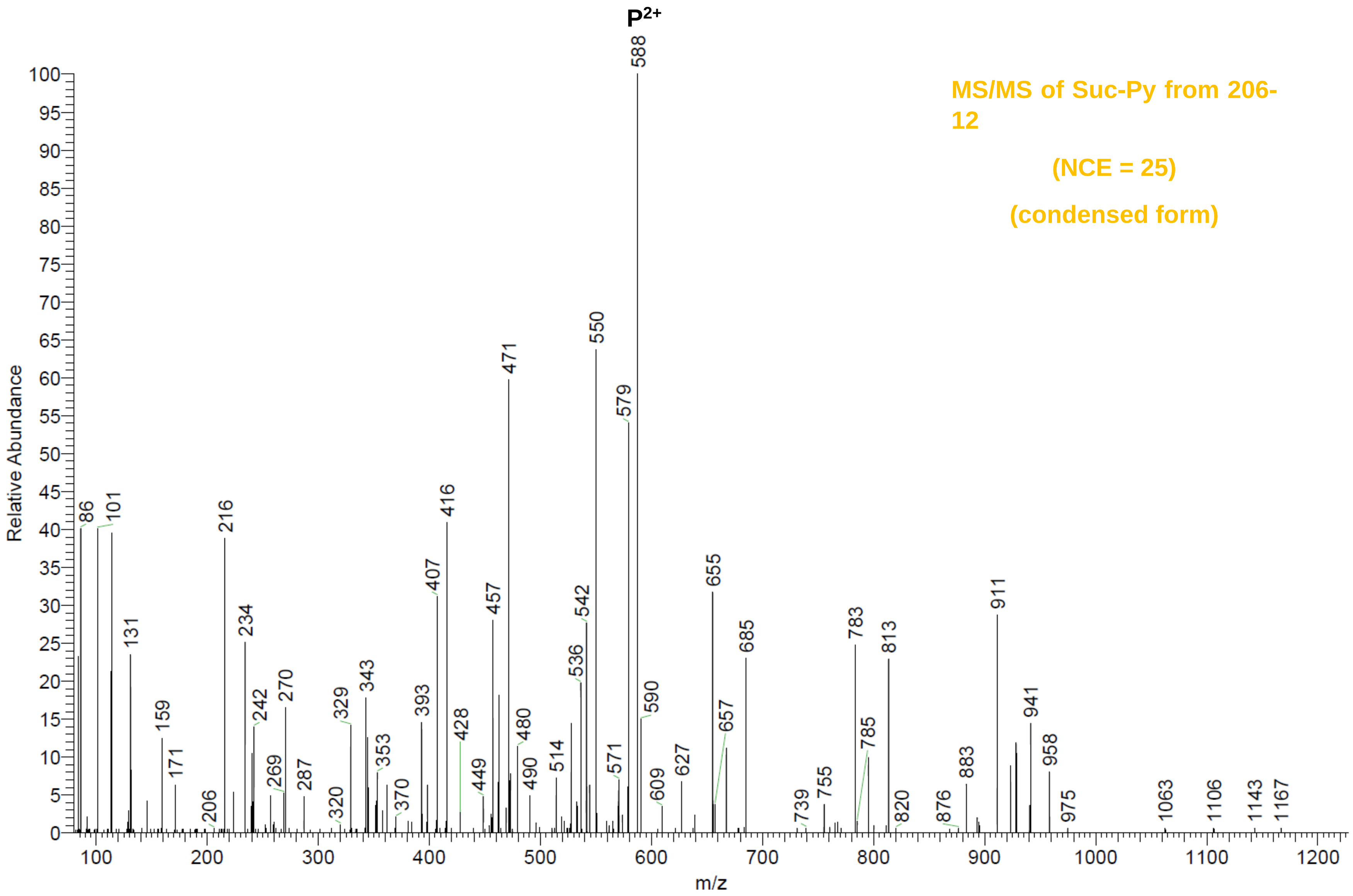

MS/MS of Suc-Py from 206-12

(NCE = 25)

(condensed form)

Table S6. Detected fragments in the MS/MS fragmentation of Suc-Py of 1-60 compared to their theoretical value *m/z* value and their ppm deviation.

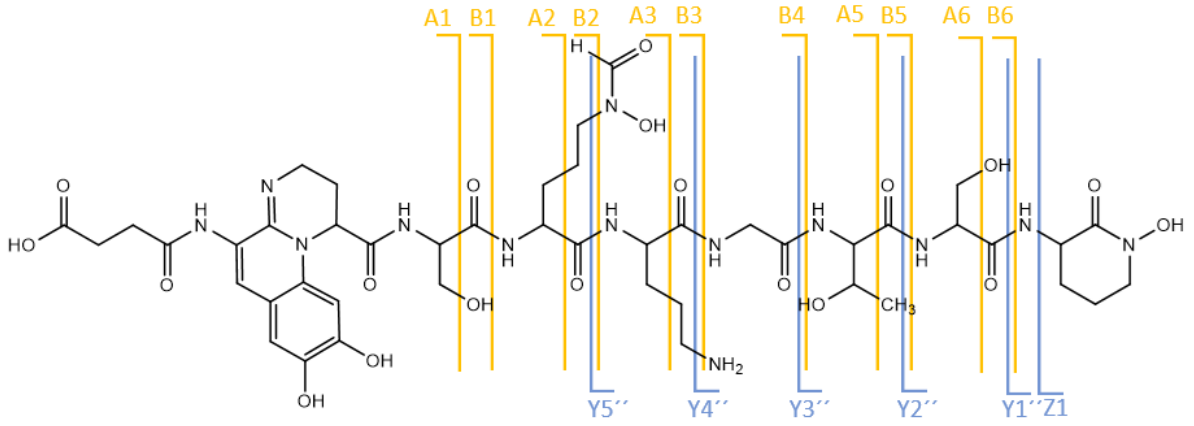

**Name:**	**1-60**			
**Sequence:**	Ser-FoOHOrn-Orn-Gly-aThr-Ser-cOHOrn			
	**Formula**	**Mass (*m/z*)**	**Found Mass (*m/z*)**	**ppm Deviation**
**[M+H]⁺**	$C_{45}H_{66}N_{13}O_{19}$	1092.45979	1092.45855	-1.14
**$[M+2H]^{2+}$**	$C_{45}H_{67}N_{13}O_{19}$	546.73381	546.73270	-2.03
**Fragment Type (NCE = 25)**	**Formula**	**Mass (*m/z*)**	**Found Mass (*m/z*)**	**ppm Deviation**
**A_{01}**	$C_{19}H_{21}N_{4}O_{7}$	417.14048	417.13998	-1.19
**A_{02}**	$C_{25}H_{31}N_{6}O_{10}$	575.20962	575.20906	-0.97
**A_{03}**	$C_{30}H_{41}N_{8}O_{11}$	689.28893	689.28904	0.16
**A_{05}**	$C_{36}H_{51}N_{10}O_{14}$	847.35807	847.35746	-0.72
**A_{06}**	$C_{39}H_{56}N_{11}O_{16}$	934.39010	934.38828	-1.95
**B_{01}**	$C_{20}H_{21}N_{4}O_{8}$	445.13539	445.13465	-1.66
**B_{02}**	$C_{26}H_{31}N_{6}O_{11}$	603.20453	603.20340	-1.88
**B_{03}**	$C_{31}H_{41}N_{8}O_{12}$	717.28385	717.28310	-1.04
**B_{04}**	$C_{33}H_{44}N_{9}O_{13}$	774.30531	774.30476	-0.71
**B_{05}**	$C_{37}H_{51}N_{10}O_{15}$	875.35299	875.35105	-2.21
**B_{06}**	$C_{40}H_{56}N_{11}O_{17}$	962.38502	962.38269	-2.42
**Y''_{01}**	$C_{5}H_{11}N_{2}O_{2}$	131.08150	131.08141	-0.72
**Y''_{02}**	$C_{8}H_{16}N_{3}O_{4}$	218.11353	218.11334	-0.88
**Y''_{03}**	$C_{12}H_{23}N_{4}O_{6}$	319.16121	319.16072	-1.54
**Y''_{04}**	$C_{14}H_{26}N_{5}O_{7}$	376.18267	376.18205	-1.66
**Y''_{05}**	$C_{25}H_{46}N_{9}O_{11}$	648.33113	648.33030	-1.28
**Z_{01}**	$C_{5}H_{8}N_{1}O_{2}$	114.05495	114.05513	1.53

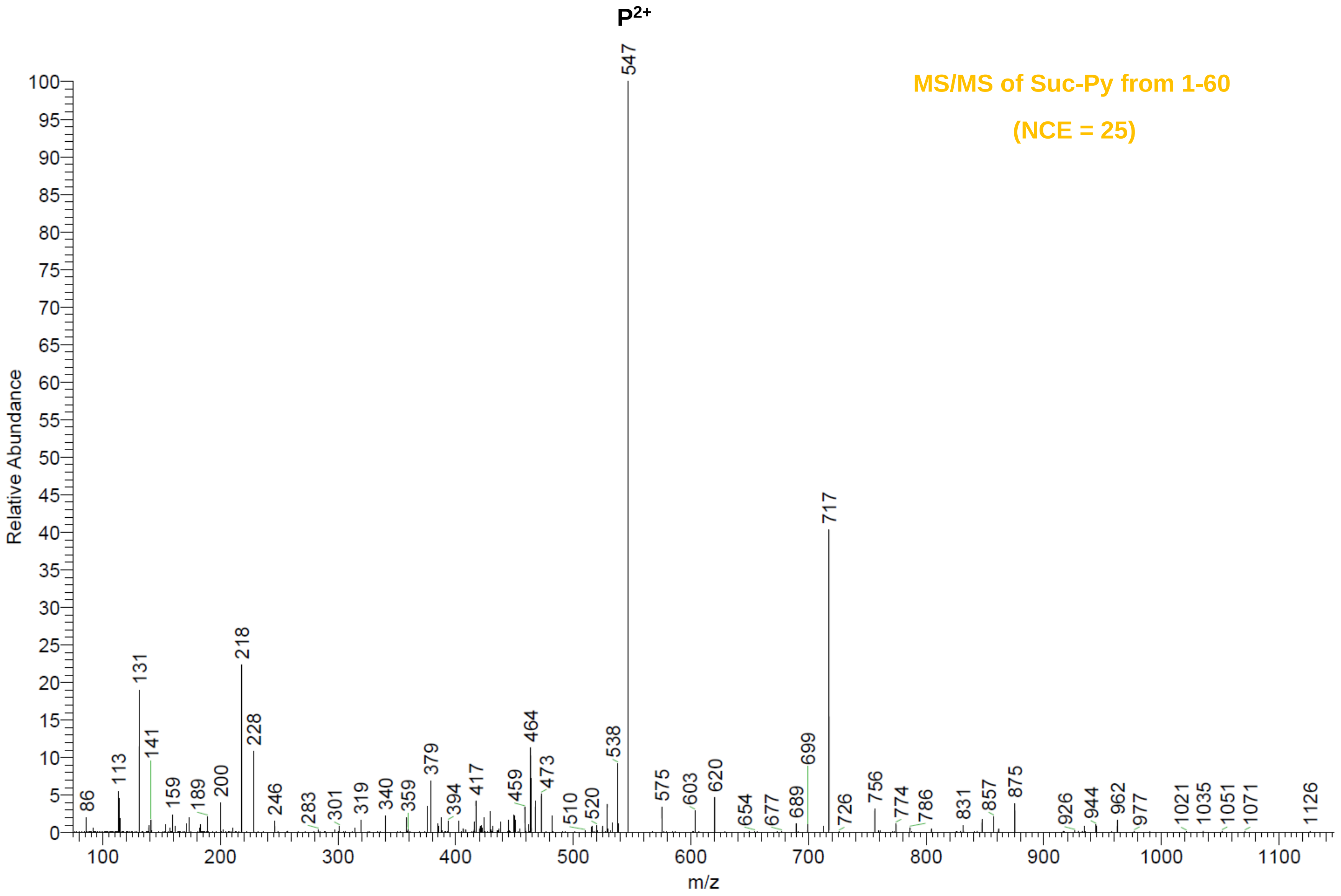
P2+
MS/MS of Suc-Py from 1-60
(NCE = 25)
Relative Abundance
m/z
547
717
218
131
228
141
113
464
538
699
379
620
473
417
459
575
603
756
875
86
159
189
200
246
283
301
319
340
359
394
510
520
654
677
689
726
774
786
831
857
926
944
962
977
1021
1035
1051
1071
1126

Table S7. Detected fragments in the MS/MS fragmentation of Suc-Py of 3A06 compared to their theoretical value *m/z* value and their ppm deviation.

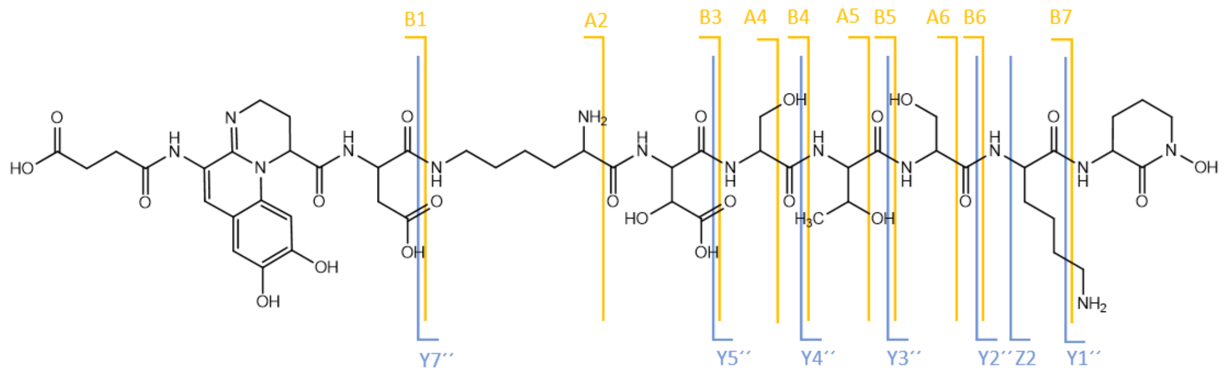

**Name:**	**3A06**			
**Sequence:**	Asp-ε-Lys-OHAsp-Ser-Thr-Ser-Lys-cOHOrn			
	**Formula**	**Mass (*m/z*)**	**Found Mass (*m/z*)**	**ppm Deviation**
**[M+H]$^+$**	$C_{52}H_{77}N_{14}O_{23}$	1265.52860	1265.52727	-1.05
**[M+2H]$^{2+}$**	$C_{52}H_{78}N_{14}O_{23}$	633.26821	633.26699	-1.93
**Fragment Type (NCE = 25)**	**Formula**	**Mass (*m/z*)**	**Found Mass (*m/z*)**	**ppm Deviation**
**A_{02}**	$C_{26}H_{33}N_6O_9$	573.23035	573.23188	2.66
**A_{04}**	$C_{33}H_{43}N_8O_{15}$	791.28424	791.28388	-0.45
**A_{05}**	$C_{37}H_{50}N_9O_{17}$	892.33192	892.33267	0.84
**A_{06}**	$C_{40}H_{55}N_{10}O_{19}$	979.36395	979.36458	0.65
**B_{01}**	$C_{21}H_{21}N_4O_9$	473.13030	473.13006	-0.52
**B_{03}**	$C_{31}H_{38}N_7O_{14}$	732.24713	732.24671	-0.57
**B_{04}**	$C_{34}H_{43}N_8O_{16}$	819.27915	819.27946	0.37
**B_{05}**	$C_{38}H_{50}N_9O_{18}$	920.32683	920.32572	-1.21
**B_{06}**	$C_{41}H_{55}N_{10}O_{20}$	1007.35886	1007.35886	-0.00
**B_{07}**	$C_{47}H_{67}N_{12}O_{21}$	1135.45382	1135.45068	-2.77
**Y’’$_{01}$**	$C_5H_{11}N_2O_2$	131.08150	131.08171	1.57
**Y’’$_{02}$**	$C_{11}H_{23}N_4O_3$	259.17647	259.17652	0.20
**Y’’$_{03}$**	$C_{14}H_{28}N_5O_5$	346.20850	346.20850	0.00
**Y’’$_{04}$**	$C_{18}H_{35}N_6O_7$	447.25617	447.25742	2.79
**Y’’$_{05}$**	$C_{21}H_{40}N_7O_9$	534.28820	534.28826	0.11
**Y’’$_{07}$**	$C_{31}H_{57}N_{10}O_{14}$	793.40502	793.40428	-0.94
**Z_{02}**	$C_{11}H_{20}N_3O_3$	242.14992	242.14994	0.09

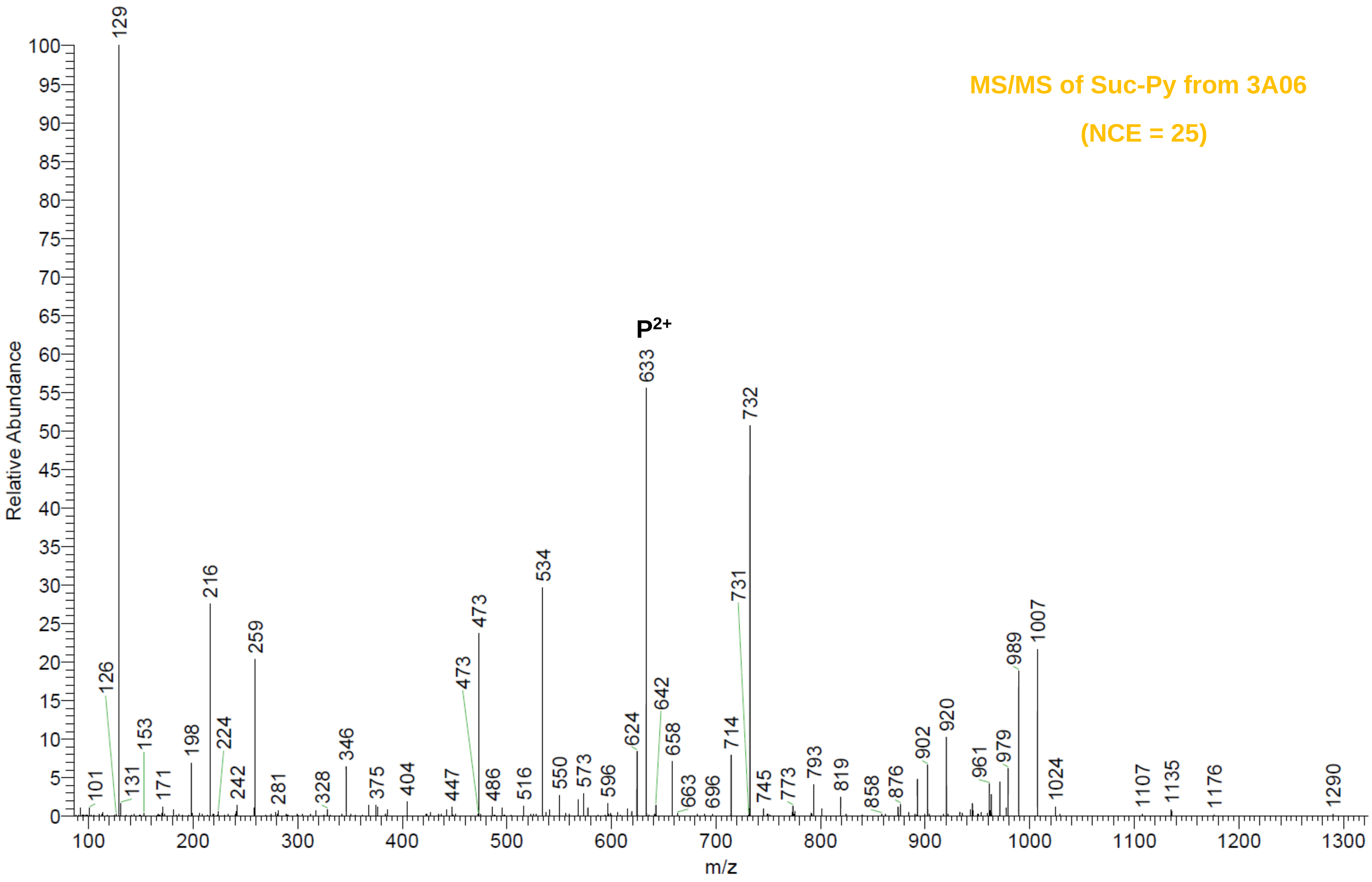
MS/MS of Suc-Py from 3A06
(NCE = 25)
P2+
Relative Abundance
m/z
129
633
732
534
731
216
473
259
1007
989
473
126
642
920
714
153
198
224
658
346
902
793
979
961
624
242
101
131
171
281
328
375
404
447
486
516
550
573
596
663
696
745
773
819
858
876
1024
1107
1135
1176
1290

Table S8. Detected fragments in the MS/MS fragmentation of Suc-Py of 3B19 compared to their theoretical value *m/z* value and their ppm deviation.

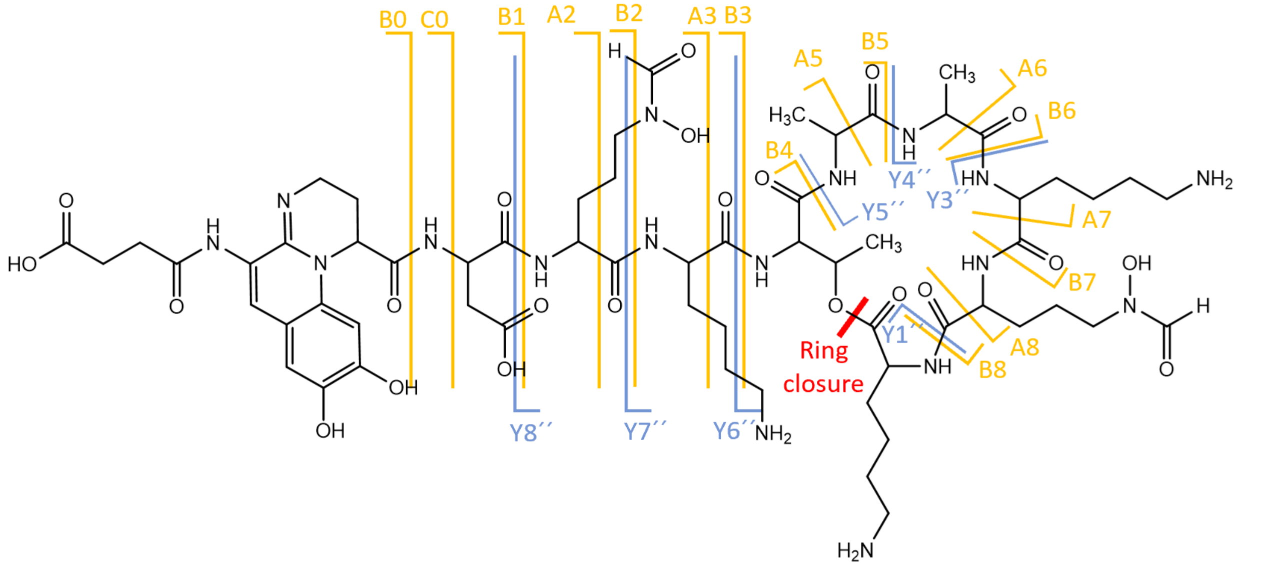

**Name:**	**3B19**			
**Sequence:**	Asp-FoOHOrn-Lys-(Thr-Ala-Ala-Lys-FoOHOrn-Lys)			
	**Formula**	**Mass (*m/z*)**	**Found Mass (*m/z*)**	**ppm Deviation**
**[M+H]$^+$**	$C_{61}H_{94}N_{17}O_{22}$	1416.67593	1416.67291	-2.13
**[M+2H]$^{2+}$**	$C_{61}H_{95}N_{17}O_{22}$	708.84188	708.83988	-2.82
**Fragment Type (NCE = 30)**	**Formula**	**Mass (*m/z*)**	**Found Mass (*m/z*)**	**ppm Deviation**
**A_{02}**	$C_{26}H_{31}N_6O_{11}$	603.20453	603.20435	-0.30
**A_{03}**	$C_{32}H_{43}N_8O_{12}$	731.29950	731.29854	-1.31
**A_{03} - H_2O**	$C_{32}H_{41}N_8O_{11}$	713.28893	713.28774	-1.67
**A_{05}**	$C_{39}H_{55}N_{10}O_{15}$	903.38429	903.38208	-2.44
**A_{05} - H_2O**	$C_{39}H_{53}N_{10}O_{14}$	885.37372	885.37277	-1.08
**A_{06}**	$C_{42}H_{60}N_{11}O_{16}$	974.42140	974.42055	-0.87
**A_{06} - H_2O**	$C_{42}H_{58}N_{11}O_{15}$	956.41084	956.40948	-1.42
**A_{07} - H_2O**	$C_{48}H_{70}N_{13}O_{16}$	1084.50580	1084.50235	-3.18
**A_{08}**	$C_{54}H_{82}N_{15}O_{20}$	1260.58551	1260.58280	-2.15
**B_{00}**	$C_{17}H_{16}N_3O_6$	358.10336	358.10336	0.00
**B_{01}**	$C_{21}H_{21}N_4O_9$	473.13030	473.13030	-0.01
**B_{01} - H_2O**	$C_{21}H_{19}N_4O_8$	455.11974	455.11985	0.24
**B_{02}**	$C_{27}H_{31}N_6O_{12}$	631.19945	631.19952	0.12
**B_{03}**	$C_{33}H_{43}N_8O_{13}$	759.29441	759.29377	-0.84
**B_{03} - H_2O**	$C_{33}H_{41}N_8O_{12}$	741.28385	741.28109	-3.72
**B_{04} - H_2O**	$C_{37}H_{48}N_9O_{14}$	842.33152	842.33132	-0.24
**B_{05}**	$C_{40}H_{55}N_{10}O_{16}$	931.37920	931.37483	-4.69
**B_{05} - H_2O**	$C_{40}H_{53}N_{10}O_{15}$	913.36864	913.36814	-0.54
**B_{06}**	$C_{43}H_{60}N11O_{17}$	1002.41632	1002.41426	-2.05
**B_{06} - H_2O**	$C_{43}H_{58}N_{11}O_{16}$	984.40575	984.40375	-2.03

**B_{07}**	$C_{49}H_{72}N_{13}O_{18}$	1130.51128	1130.51290	1.43
**B_{07} - H_2O**	$C_{49}H_{70}N_{13}O_{17}$	1112.50071	1112.50077	0.05
**B_{08}**	$C_{55}H_{82}N_{15}O_{21}$	1288.58042	1288.57827	-1.67
**C_{00}**	$C_{17}H_{17}N_4O_6$	373.11426	373.11430	0.11
**X_{05}**	$C_{22}H_{38}N_7O_7$	512.28272	512.28188	-1.65
**Y''_{01}**	$C_{40}H_{74}N_{13}O_{13}$	944.55236	944.55145	-0.96
**Y''_{03}**	$C_{28}H_{52}N_9O_9$	658.38825	658.38827	0.03
**Y''_{04} + H_2O**	$C_{24}H_{47}N_8O_8$	575.35114	575.35194	1.40
**Y''_{05} + H_2O**	$C_{21}H_{42}N_7O_7$	504.31402	504.31550	2.93
**Y''_{06} + H_2O**	$C_{18}H_{37}N_6O_6$	433.27691	433.27648	-0.99
**Y''_{06}**	$C_{18}H_{35}N_6O_5$	415.26634	415.26634	-0.01
**Y''_{07} + H_2O**	$C_{12}H_{25}N_4O_5$	305.18195	305.18187	-0.25
**Y''_{07}**	$C_{12}H_{23}N_4O_4$	287.17138	287.17150	0.41
**Y''_{08} + H_2O**	$C_6H_{15}N_2O_2$	147.11280	147.11278	-0.16
**Y''_{08}**	$C_6H_{13}N_2O_1$	129.10224	129.10241	1.32

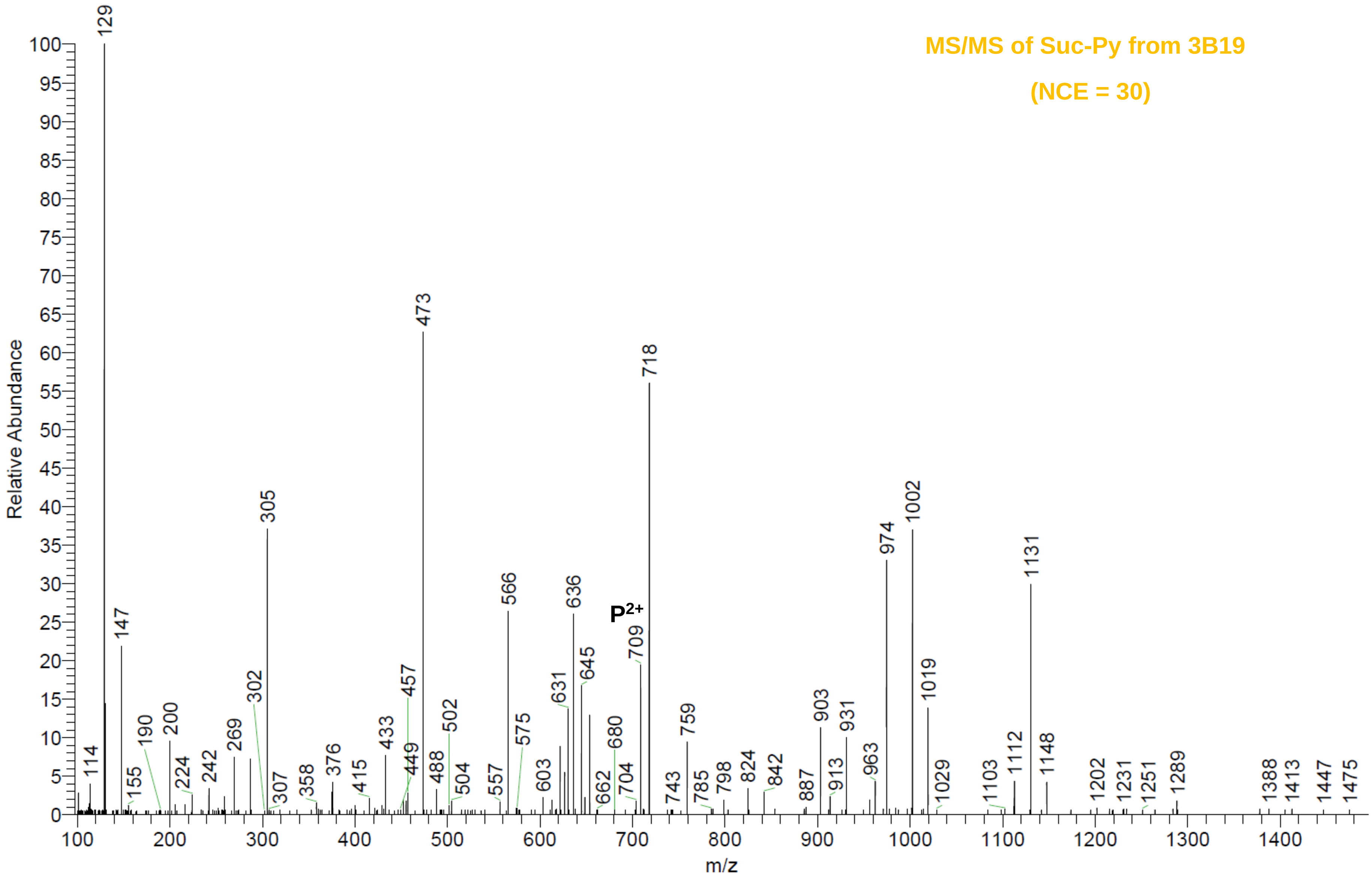
MS/MS of Suc-Py from 3B19
(NCE = 30)
Relative Abundance
m/z
P2+
129
147
473
718
305
566
636
1002
974
1131

Table S9. Detected fragments in the MS/MS fragmentation of Glu-IsoPy of 3C16 compared to their theoretical value *m/z* value and their ppm deviation.

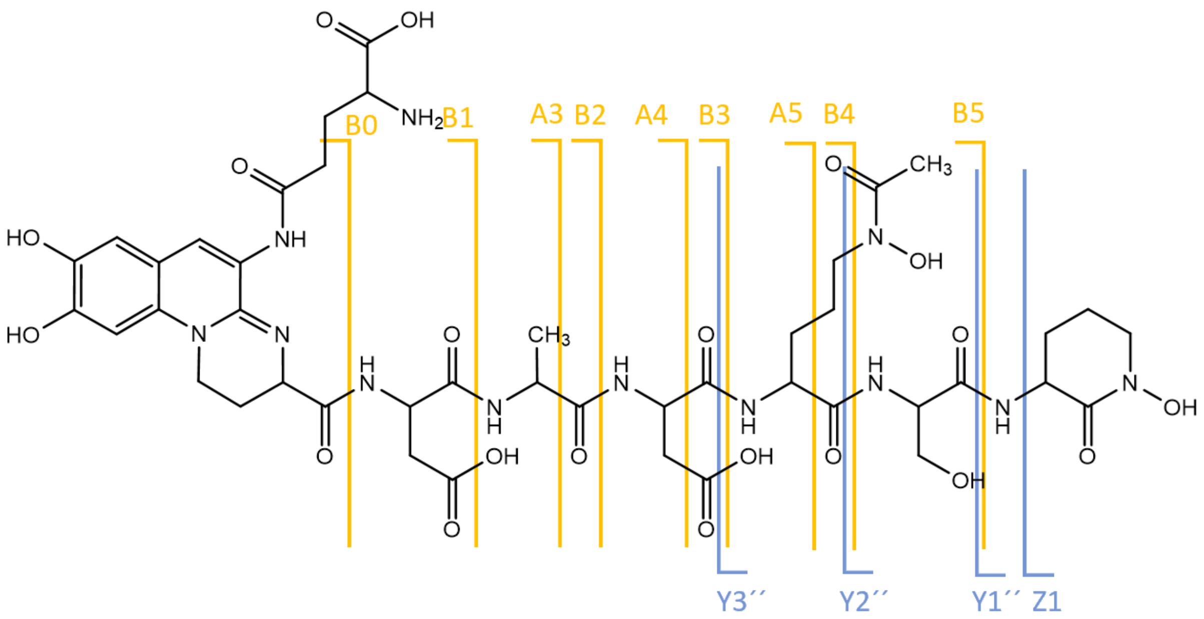

**Name:**	**3C16**			
**Sequence:**	Asp-Ala-Asp-AcOHOrn-Ser-cOHOrn			
	**Formula**	**Mass (*m/z*)**	**Found Mass (*m/z*)**	**ppm Deviation**
**[M+H]+**	$C_{44}H_{61}N_{12}O_{20}$	1077.412508	1077.40998	-2.35
**[M+2H]2+**	$C_{44}H_{62}N_{12}O_{20}$	539.2101663	539.20861	-2.89
**Fragment Type (NCE = 25)**	**Formula**	**Mass (*m/z*)**	**Found Mass (*m/z*)**	**ppm Deviation**
**B_{00}**	$C_{18}H_{19}N_4O_6$	387.12991	387.12869	-3.15
**B_{01}**	$C_{22}H_{24}N_5O_9$	502.15685	502.15600	-1.70
**B_{02}**	$C_{25}H_{29}N_6O_{10}$	573.19397	573.19337	-1.04
**B_{03}**	$C_{29}H_{34}N_7O_{13}$	688.22091	688.22025	-0.96
**B_{04}**	$C_{36}H_{46}N_9O_{16}$	860.30570	860.30416	-1.79
**B_{05}**	$C_{39}H_{51}N_{10}O_{18}$	947.33773	947.33626	-1.55
**Y''_{03}**	$C_{15}H_{28}N_5O_7$	390.19832	390.19763	-1.78
**Y''_{02}**	$C_8H_{16}N_3O_4$	218.11353	218.11319	-1.57
**Y''_{01}**	$C_5H_{11}N_2O_2$	131.08150	131.08142	-0.64
**Z_{01}**	$C_5H_8N_1O_2$	114.05495	114.05508	1.10

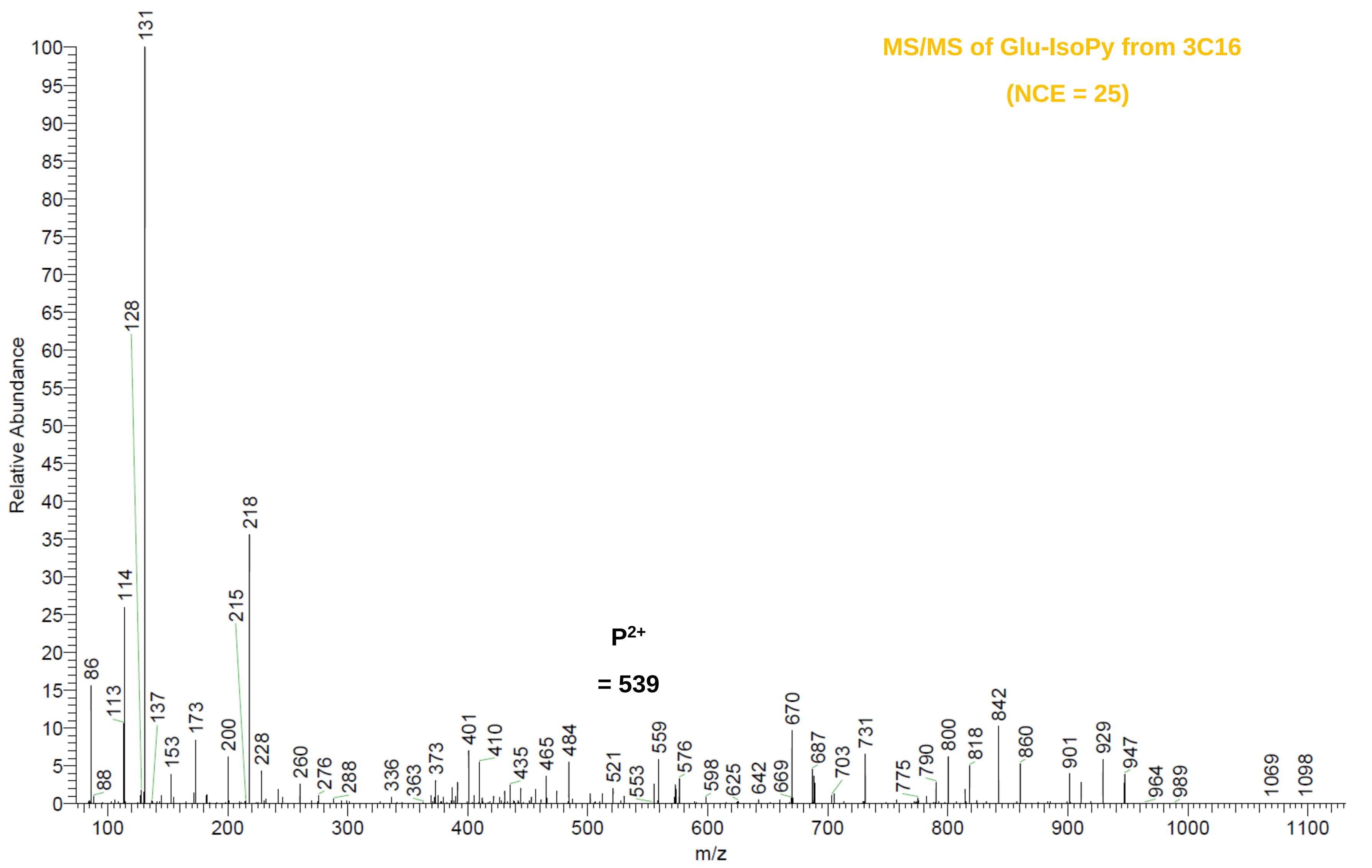
MS/MS of Glu-IsoPy from 3C16
(NCE = 25)
P^{2+}
= 539
Relative Abundance
m/z
86
88
113
114
128
131
137
153
173
200
215
218
228
260
276
288
336
363
373
401
410
435
465
484
521
553
559
576
598
625
642
669
670
687
703
731
775
790
800
818
842
860
901
929
947
964
989
1069
1098

Table S10. Detected fragments in the MS/MS fragmentation of Suc-Py of 3D19 compared to their theoretical value *m/z* value and their ppm deviation.

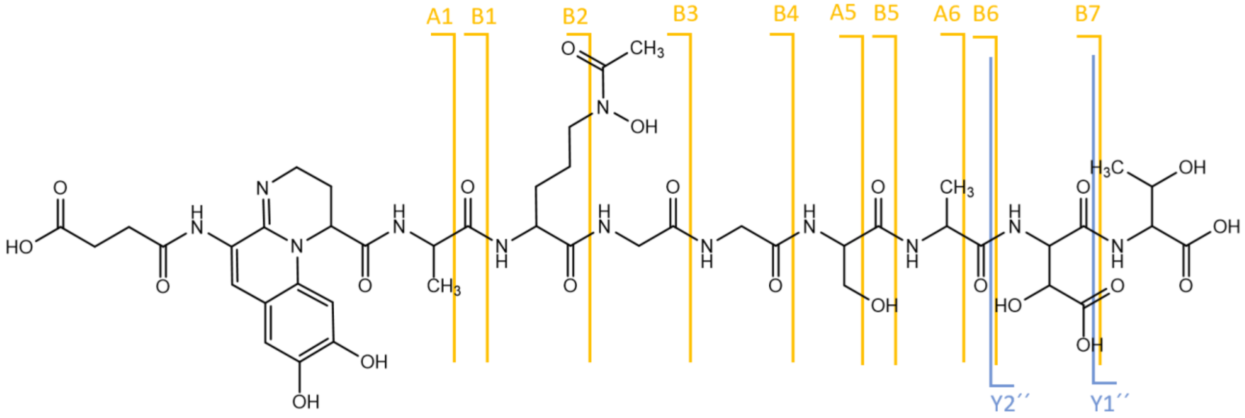

**Name:**	3D19			
**Sequence:**	Ala-AcOHOrn-Gly-Gly-Ser-Ala-OHAsp-Thr			
	**Formula**	**Mass (*m/z*)**	**Found Mass (*m/z*)**	**ppm Deviation**
**[M+H]+**	$C_{45}H_{63}N_{12}O_{22}$	1123.41799	1123.41840	0.37
**[M+2H]2+**	$C_{45}H_{64}N_{12}O_{22}$	562.21291	562.21255	-0.63
**Fragment Type (NCE = 25)**	**Formula**	**Mass (*m/z*)**	**Found Mass (*m/z*)**	**ppm Deviation**
**A_{01}**	$C_{19}H_{21}N_4O_6$	401.14556	401.14540	-0.40
**A_{05}**	$C_{33}H_{44}N_9O_{13}$	774.30531	774.30328	-2.62
**A_{06}**	$C_{36}H_{49}N_{10}O_{14}$	845.34242	845.34110	-1.56
**B_{01}**	$C_{20}H_{21}N_4O_7$	429.14048	429.14058	0.24
**B_{02}**	$C_{27}H_{33}N_6O_{10}$	601.22527	601.22670	2.38
**B_{03}**	$C_{29}H_{36}N_7O_{11}$	658.24673	658.24372	-4.58
**B_{04}**	$C_{31}H_{39}N_8O_{12}$	715.26820	715.26797	-0.31
**B_{05}**	$C_{34}H_{44}N_9O_{14}$	802.30022	802.30008	-0.18
**B_{06}**	$C_{37}H_{49}N_{10}O_{15}$	873.33734	873.33723	-0.12
**B_{07}**	$C_{41}H_{54}N_{11}O_{19}$	1004.35920	1004.35544	-3.74
**Y''_{02}**	$C_8H_{15}N_2O_7$	251.08738	251.08715	-0.91
**Y''_{01}**	$C_4H_{10}N_1O_3$	120.06552	120.06578	2.17

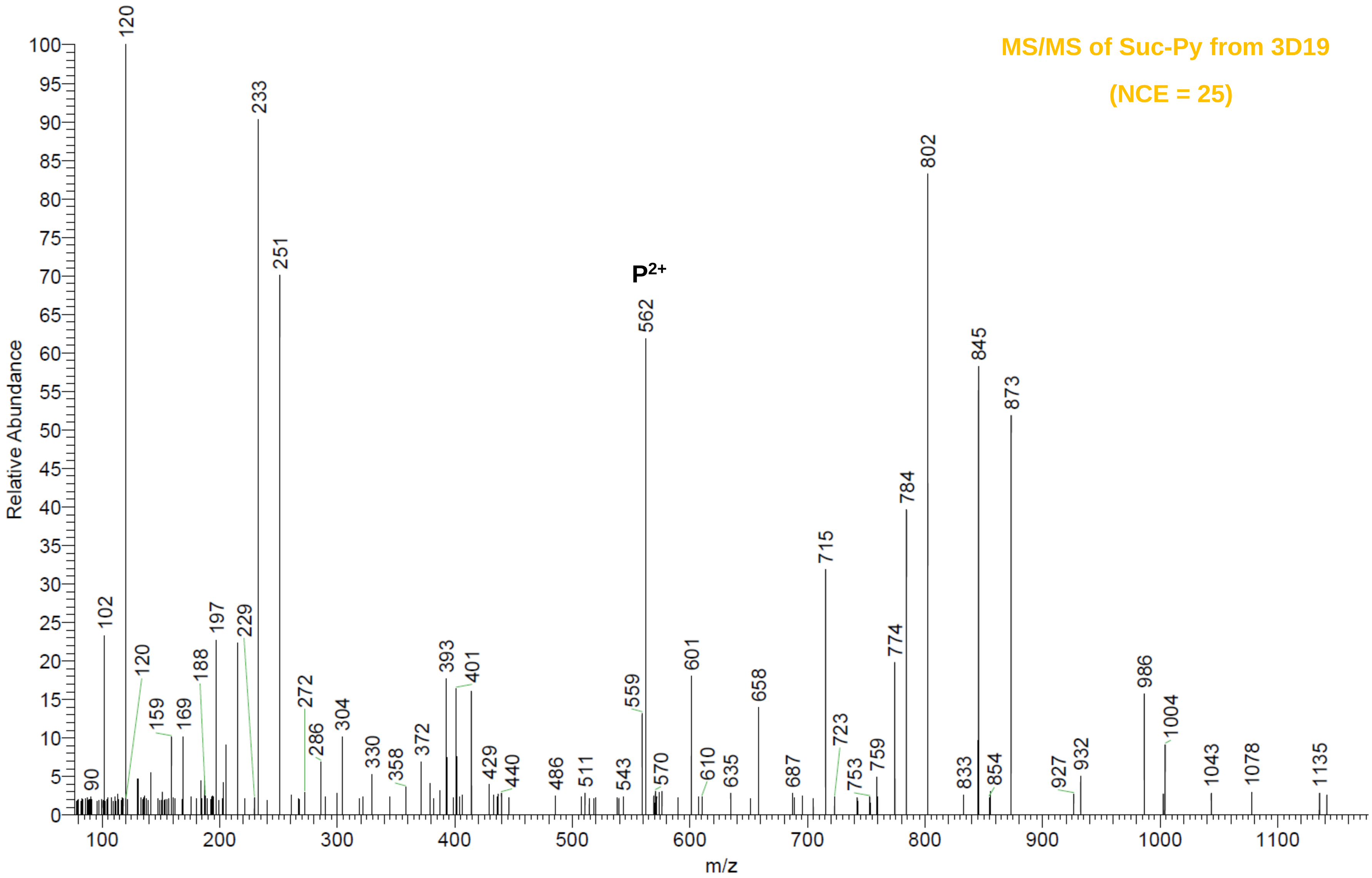
MS/MS of Suc-Py from 3D19
(NCE = 25)
P2+
Relative Abundance
m/z
120
233
802
251
562
845
873
784
715
102
197
229
120
188
159
169
272
286
304
330
358
372
393
401
429
440
486
511
543
559
570
601
610
635
658
687
723
753
759
774
833
854
927
932
986
1004
1043
1078
1135
90

Table S11. Detected fragments in the MS/MS fragmentation of Suc-Py of 3F12 compared to their theoretical value *m/z* value and their ppm deviation.

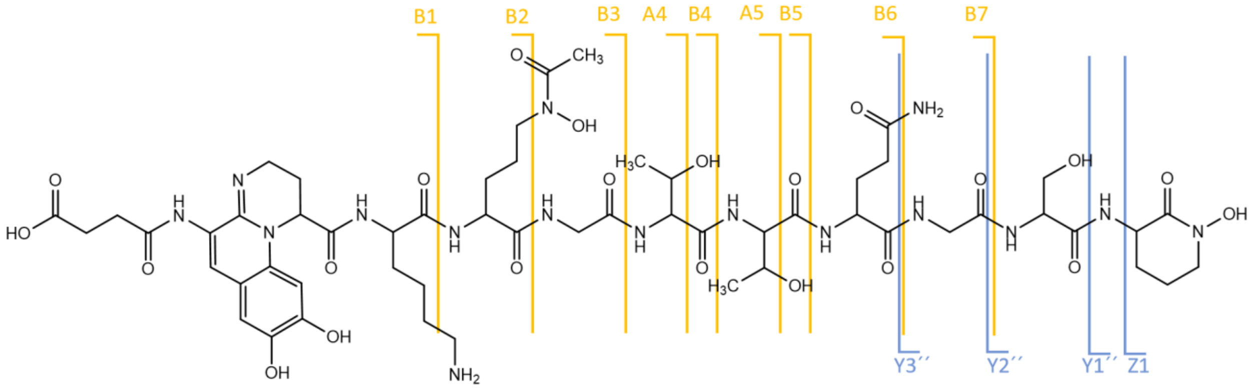

**Name:**	**3F12**			
**Sequence:**	Lys-AcOHOrn-Gly-Thr-Thr-Gln-Gly-Ser-cOHOrn			
	**Formula**	**Mass (*m/z*)**	**Found Mass (*m/z*)**	**ppm Deviation**
**[M+H]⁺**	$C_{55}H_{83}N_{16}O_{22}$	1319.58678	1319.58741	0.47
**[M+2H]²⁺**	$C_{55}H_{84}N_{16}O_{22}$	660.29730	660.29664	-1.00
**Fragment Type (NCE = 30)**	**Formula**	**Mass (*m/z*)**	**Found Mass (*m/z*)**	**ppm Deviation**
**A_{03}**	$C_{31}H_{43}N_{8}O_{10}$	687.30967	687.30655	-4.53
**A_{04}**	$C_{35}H_{50}N_{9}O_{12}$	788.35734	788.35401	-4.23
**A_{05}**	$C_{39}H_{57}N_{10}O_{14}$	889.40502	889.40422	-0.90
**B_{01}**	$C_{23}H_{28}N_{5}O_{7}$	486.19832	486.19770	-1.28
**B_{02}**	$C_{30}H_{40}N_{7}O_{10}$	658.28311	658.28177	-2.05
**B_{03}**	$C_{32}H_{43}N_{8}O_{11}$	715.30458	715.3024	-3.05
**B_{04}**	$C_{36}H_{50}N_{9}O_{13}$	816.35226	816.35041	-2.27
**B_{05}**	$C_{40}H_{57}N_{10}O_{15}$	917.39994	917.39858	-1.48
**B_{06}**	$C_{45}H_{65}N_{12}O_{17}$	1045.45851	1045.45688	-1.56
**B_{07}**	$C_{47}H_{68}N_{13}O_{18}$	1102.47998	1102.47896	-0.92
**Y''_{01}**	$C_{5}H_{11}N_{2}O_{2}$	131.08150	131.08155	0.35
**Y''_{02}**	$C_{8}H_{16}N_{3}O_{4}$	218.11353	218.11344	-0.42
**Y''_{03}**	$C_{10}H_{19}N_{4}O_{5}$	275.13500	275.13510	0.38
**Z_{01}**	$C_{5}H_{8}N_{1}O_{2}$	114.05495	114.05522	2.32

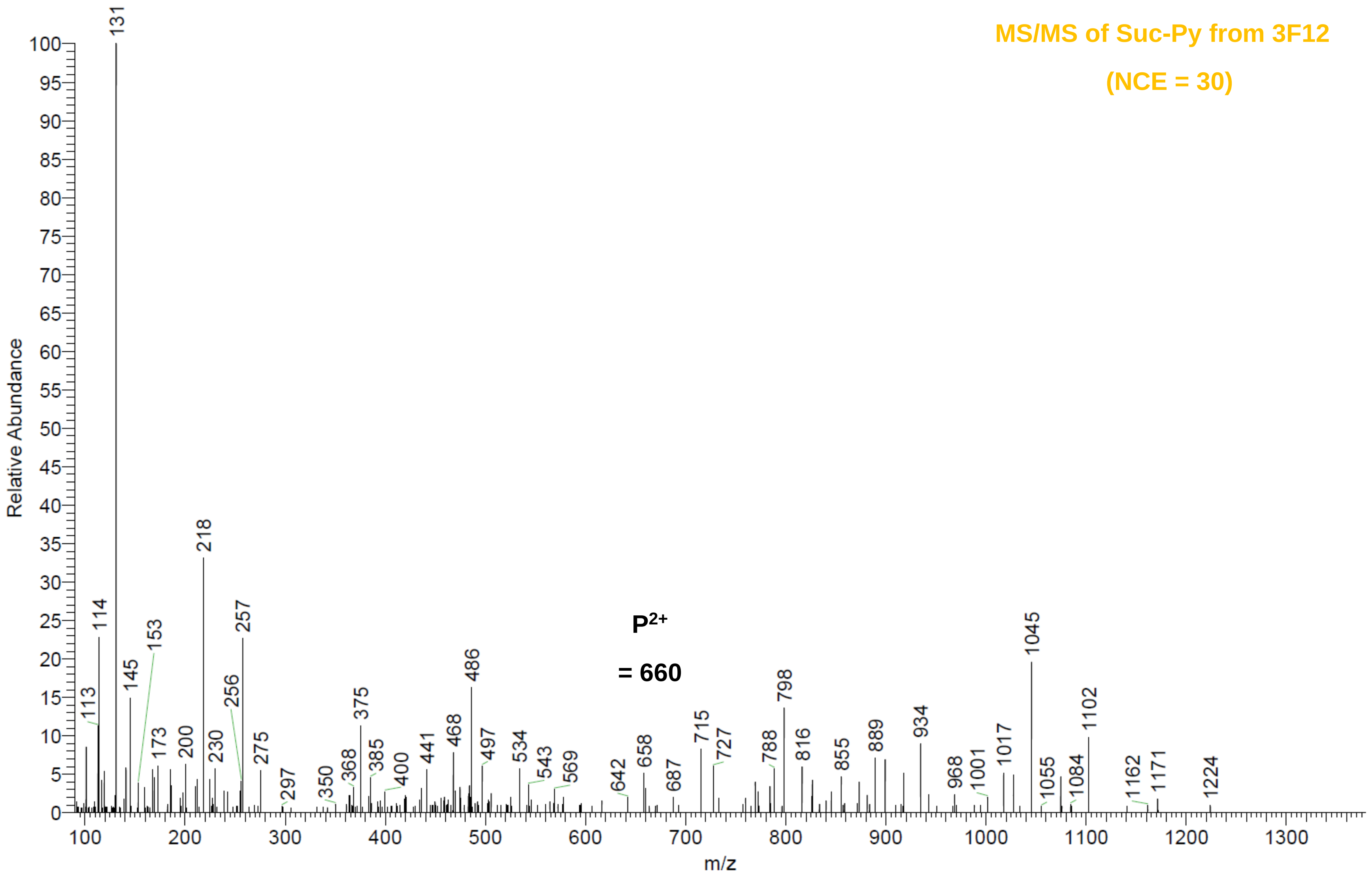
MS/MS of Suc-Py from 3F12
(NCE = 30)
P2+
= 660
Relative Abundance
m/z
131
218
114
257
153
145
113
173
200
230
256
275
297
350
368
375
385
400
441
468
486
497
534
543
569
642
658
687
715
727
788
798
816
855
889
934
968
1001
1017
1045
1055
1084
1102
1162
1171
1224

Table S12. Detected fragments in the MS/MS fragmentation of Suc-Py of 3G07 compared to their theoretical value *m/z* value and their ppm deviation.

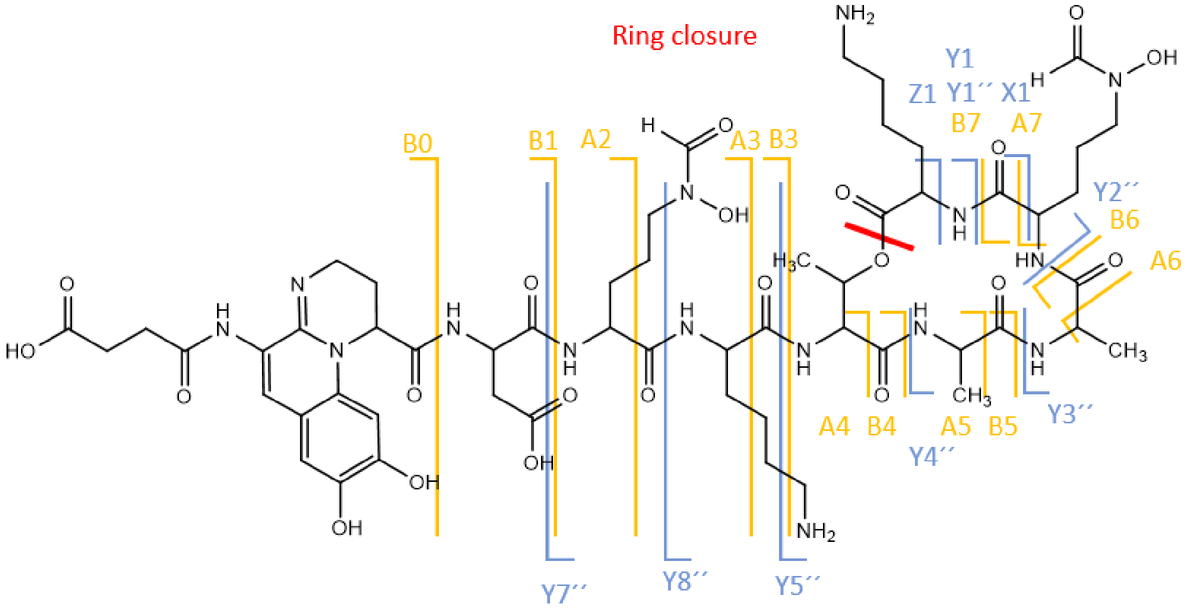

**Name:**	**3G07**			
**Sequence:**	Asp-FoOHOrn-Lys-(Thr-Ala-Ala-FoOHOrn-Lys)			
	**Formula**	**Mass (*m/z*)**	**Found Mass (*m/z*)**	**ppm Deviation**
**[M+H]$^+$**	$C_{55}H_{82}N_{15}O_{21}$	1288.58097	1288.58102	0.04
**[M+2H]$^{2+}$**	$C_{55}H_{83}N_{15}O_{21}$	644.79440	644.79380	-0.93
**Fragment Type (NCE = 30)**	**Formula**	**Mass (*m/z*)**	**Found Mass (*m/z*)**	**ppm Deviation**
**A_{02}**	$C_{26}H_{31}N_{6}O_{11}$	603.20453	603.20410	-0.72
**A_{03}**	$C_{32}H_{43}N_{8}O_{12}$	731.29950	731.29831	-1.62
**A_{04}**	$C_{36}H_{50}N_{9}O_{14}$	832.34717	832.34713	-0.05
**A_{05}**	$C_{39}H_{55}N_{10}O_{15}$	903.38429	903.38324	-1.16
**A_{06}**	$C_{42}H_{60}N_{11}O_{16}$	974.42140	974.42072	-0.70
**A_{07}**	$C_{48}H_{70}N_{13}O_{19}$	1132.49054	1132.49132	0.69
**B_{00}**	$C_{17}H_{16}N_{3}O_{6}$	358.10336	358.10304	-0.90
**B_{01}**	$C_{21}H_{21}N_{4}O_{9}$	473.13030	473.12982	-1.02
**B_{03}**	$C_{33}H_{43}N_{8}O_{13}$	759.29441	759.29389	-0.68
**B_{04}**	$C_{37}H_{50}N_{9}O_{15}$	860.34209	860.34121	-1.02
**B_{05}**	$C_{40}H_{55}N_{10}O_{16}$	931.37920	931.37790	-1.40
**B_{06}**	$C_{43}H_{60}N_{11}O_{17}$	1002.41632	1002.41518	-1.13
**B_{07}**	$C_{49}H_{70}N_{13}O_{20}$	1160.48546	1160.48485	-0.52
**X_{01}**	$C_{7}H_{11}N_{2}O_{2}$	155.08150	155.08140	-0.67
**X_{01} + H_2O**	$C_{7}H_{13}N_{2}O_{3}$	173.09207	173.09199	-0.45
**Y_{01}**	$C_{6}H_{11}N_{2}O_{1}$	127.08659	127.08668	0.71
**Y''_{01}**	$C_{6}H_{13}N_{2}O_{1}$	129.10224	129.10231	0.55
**Y''_{01} + H_2O**	$C_{6}H_{15}N_{2}O_{2}$	147.11280	147.11268	-0.84

**Y''$_{02}$**	$C_{12}H_{23}N_4O_4$	287.17138	287.17107	-1.09
**Y''$_{02}$ + H_2O**	$C_{12}H_{25}N_4O_5$	305.18195	305.18155	-1.30
**Y''$_{03}$ + H_2O**	$C_{15}H_{30}N_5O_6$	376.21906	376.21880	-0.69
**Y''$_{04}$ + H_2O**	$C_{18}H_{35}N_6O_7$	447.25617	447.25584	-0.75
**Y''$_{05}$**	$C_{22}H_{40}N_7O_8$	530.29329	530.29287	-0.79
**Y''$_{06}$**	$C_{28}H_{52}N_9O_9$	658.38825	658.38749	-1.16
**Y''$_{07}$**	$C_{34}H_{62}N_{11}O_{12}$	816.45739	816.45703	-0.44
**Z$_{01}$ + H_2O**	$C_6H_{10}N_1O_1$	112.07569	112.07592	2.05
**Z$_{01}$ + H_2O**	$C_6H_{12}N_1O_2$	130.08626	130.08633	0.58

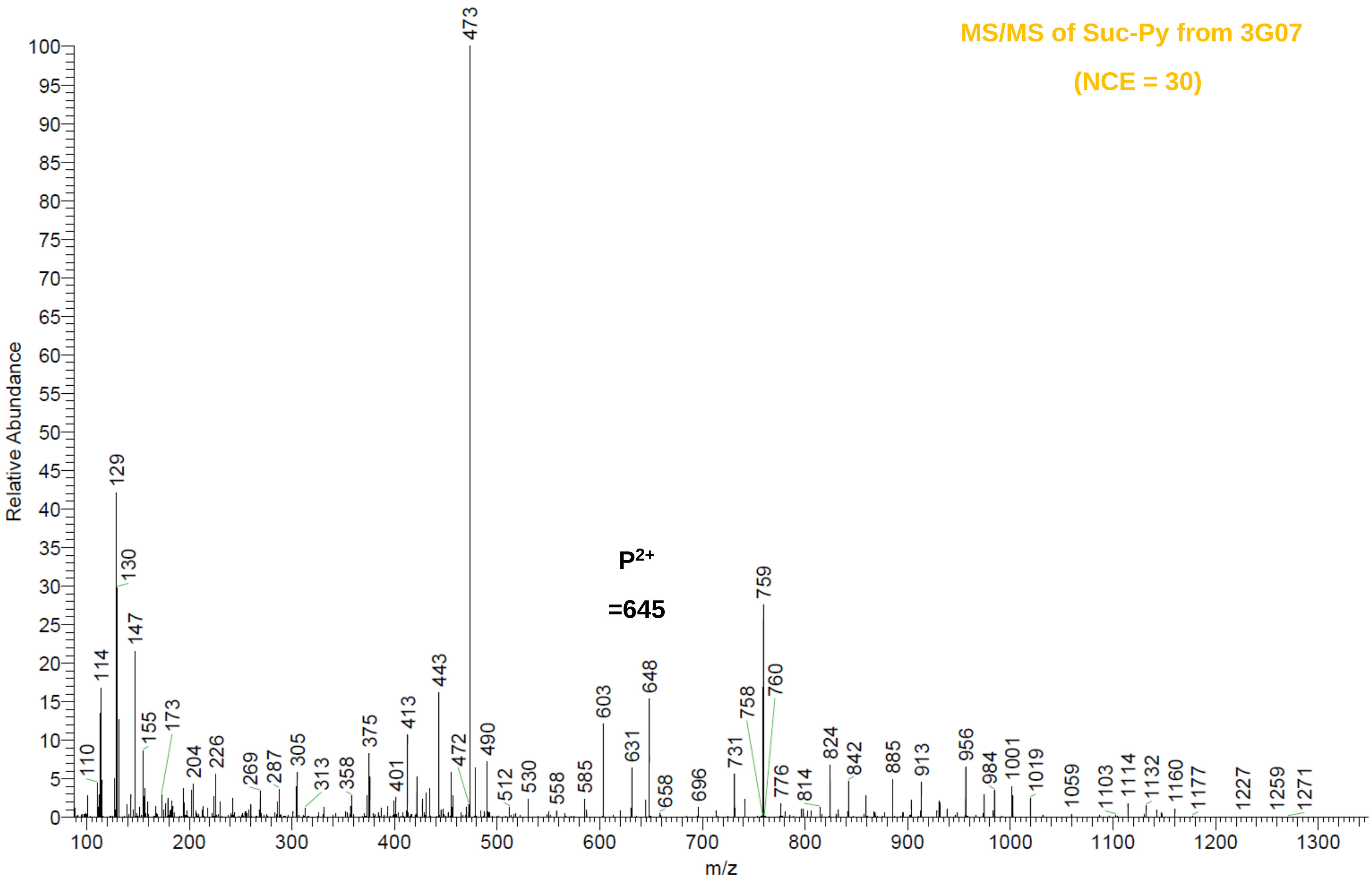
MS/MS of Suc-Py from 3G07
(NCE = 30)
P^{2+}
=645
Relative Abundance
m/z
473
129
130
147
114
110
155
173
204
226
269
287
305
313
358
375
401
413
443
472
490
512
530
558
585
603
631
648
658
696
731
758
759
760
776
814
824
842
885
913
956
984
1001
1019
1059
1103
1114
1132
1160
1177
1227
1259
1271

Table S13. Detected fragments in the MS/MS fragmentation of Suc-Py of S3a05 compared to their theoretical value *m/z* value and their ppm deviation.

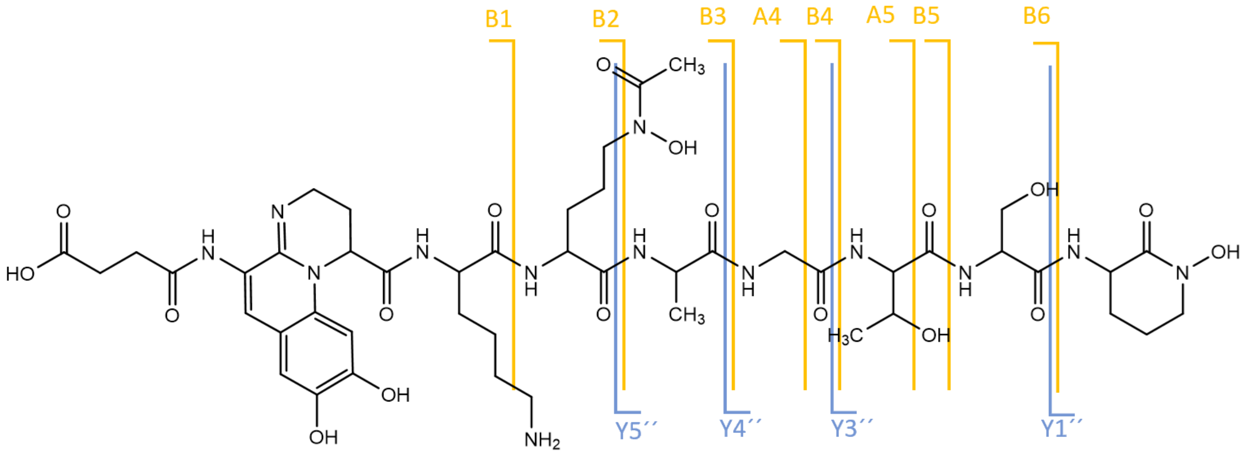

**Name:**	**S3a-05**			
**Sequence:**	Lys-AcOHOrn-Ala-Gly-aThr-Ser-cOHOrn			
	**Formula**	**Mass (*m/z*)**	**Found Mass (*m/z*)**	**ppm Deviation**
**[M+H]⁺**	$C_{47}H_{70}N_{13}O_{18}$	1104.49618	1104.49568	-0.45
**[M+2H]²⁺**	$C_{47}H_{71}N_{13}O_{18}$	552.75200	552.75159	-0.74
**Fragment Type (NCE = 25)**	**Formula**	**Mass (*m/z*)**	**Found Mass (*m/z*)**	**ppm Deviation**
**A_{04}**	$C_{34}H_{48}N_9O_{11}$	758.34678	758.34384	-3.88
**A_{05}**	$C_{38}H_{55}N_{10}O_{13}$	859.39446	859.39089	-4.15
**B_{01}**	$C_{23}H_{28}N_5O_7$	486.19832	486.19708	-2.56
**B_{02}**	$C_{30}H_{40}N_7O_{10}$	658.28312	658.28144	-2.55
**B_{03}**	$C_{33}H_{45}N_8O_{11}$	729.32023	729.31786	-3.25
**B_{04}**	$C_{35}H_{48}N_9O_{12}$	786.34169	786.33893	-3.52
**B_{05}**	$C_{39}H_{55}N_{10}O_{14}$	887.38937	887.38634	-3.42
**B_{06}**	$C_{42}H_{60}N_{11}O_{16}$	974.42140	974.41706	-4.46
**Y''_{01}**	$C_{24}H_{43}N_8O_{11}$	619.30458	619.30226	-3.75
**Y''_{03}**	$C_{14}H_{26}N_5O_7$	376.18267	376.18203	-1.71
**Y''_{04}**	$C_{12}H_{23}N_4O_6$	319.16121	319.16056	-2.04
**Y''_{05}**	$C_8H_{16}N_3O_4$	218.11353	218.11295	-2.67

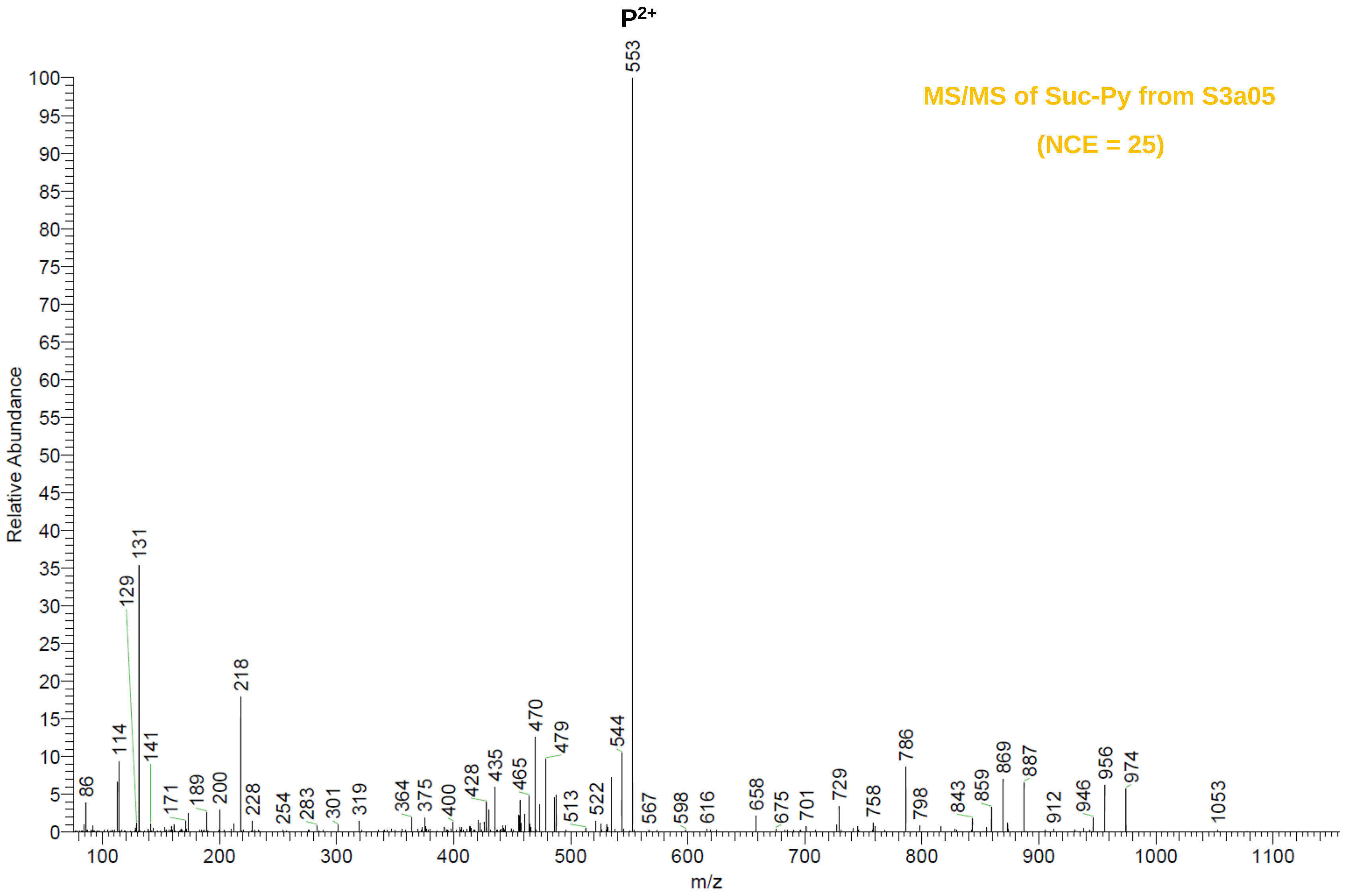
P2+
MS/MS of Suc-Py from S3a05
(NCE = 25)
Relative Abundance
m/z
553
131
129
218
114
141
86
470
479
544
786
869
887
956
974
435
428
465
1053

Table S14. Detected fragments in the MS/MS fragmentation of Suc-Py of S3a20 compared to their theoretical value *m/z* value and their ppm deviation. Additional spectral interpretation of H-FerB and Glu-FerB fragments are attached.

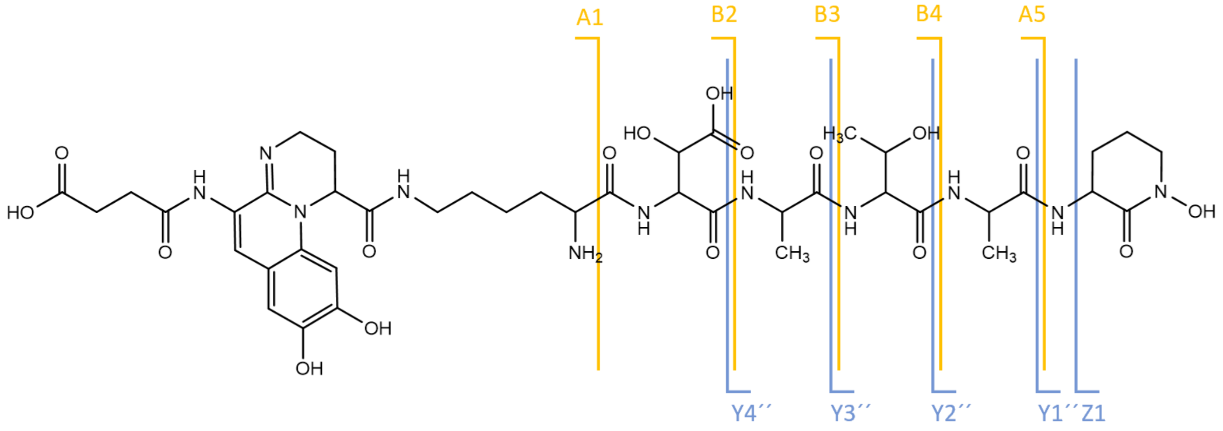

**Name:**	**S3a-20**			
**Sequence:**	ε-Lys-OHAsp-Ala-aThr-Ala-cOHOrn			
	**Formula**	**Mass (*m/z*)**	**Found Mass (*m/z*)**	**ppm Deviation**
**[M+H]⁺**	$C_{42}H_{60}N_{11}O_{17}$	990.41686	990.41468	-2.21
**[M+2H]²⁺**	$C_{42}H_{61}N_{11}O_{17}$	495.71234	495.71140	-1.91
**Fragment Type (NCE = 20)**	**Formula**	**Mass (*m/z*)**	**Found Mass (*m/z*)**	**ppm Deviation**
**A_{01}**	$C_{22}H_{28}N_5O_6$	458.20341	458.20218	-2.68
**B_{02}**	$C_{27}H_{33}N_6O_{11}$	617.22018	617.21892	-2.05
**B_{03}**	$C_{30}H_{38}N_7O_{12}$	688.25730	688.25537	-2.80
**B_{04}**	$C_{34}H_{45}N_8O_{14}$	789.30497	789.30309	-2.39
**B_{05}**	$C_{37}H_{50}N_9O_{15}$	860.34209	860.33927	-3.28
**Y''_{04}**	$C_{15}H_{28}N_5O_6$	374.20341	374.20268	-1.95
**Y''_{03}**	$C_{12}H_{23}N_4O_5$	303.16630	303.16542	-2.89
**Y''_{02}**	$C_8H_{16}N_3O_3$	202.11862	202.11808	-2.66
**Y''_{01}**	$C_5H_{11}N_2O_2$	131.08150	131.08128	-1.71
**Z_{01}**	$C_5H_8N_1O_2$	114.05550	114.05499	-4.50
**Additional information**				
**Ferribactin**	H-FerB			
	**Formula**	**Mass (*m/z*)**	**Found Mass (*m/z*)**	**ppm Deviation**
**[M+H]⁺**	$C_{38}H_{60}N_{11}O_{13}$	878.43721	878.43576	-1.65
**[M+2H]²⁺**	$C_{38}H_{61}N_{11}O_{13}$	439.72252	439.72191	-1.38
**Fragment Type**	**Formula**	**Mass (*m/z*)**	**Found Mass (*m/z*)**	**ppm Deviation**
**A_{01}**	$C_{18}H_{28}N_5O_2$	346.22375	346.22343	-0.93
**A_{03}**	$C_{25}H_{38}N_7O_7$	548.28272	548.28148	-2.27
**B_{02}**	$C_{23}H_{33}N_6O_7$	505.24052	505.24051	-0.03
**B_{03}**	$C_{26}H_{38}N_7O_8$	576.27764	576.27706	-1.00

**B_{04}**	$C_{30}H_{45}N_8O_{10}$	677.32532	677.32404	-1.88
**B_{05}**	C33$H_{50}N_9O_{11}$	748.36243	748.36101	-1.90
**Y''$_{02}$**	$C_{15}H_{28}N_5O_6$	374.20341	374.20289	-1.39
**Y''$_{03}$**	$C_{12}H_{23}N_4O_5$	303.16630	303.16586	-1.44
**Y''$_{04}$**	C_8H_{16}N3O_3	202.11862	202.11841	-1.03
**Y''$_{05}$**	$C_5H_{11}N_2O_2$	131.08150	131.08145	-0.41
**Z_{05}**	$C_5H_8N_1O_2$	114.05495	114.05510	1.27
**Ferribactin**	Glu-FerB			
	**Formula**	**Mass (*m/z*)**	**Found Mass (*m/z*)**	**ppm Deviation**
**$[M+H]^+$**	$C_{43}H_{67}N_{11}O_{16}$	1007.47980	1007.47939	-0.41
**$[M+2H]^{2+}$**	$C_{43}H_{68}N_{11}O_{16}$	504.24381	504.24334	-0.94
**Fragment Type**	**Formula**	**Mass (*m/z*)**	**Found Mass (*m/z*)**	**ppm Deviation**
**A_{01}**	$C_{23}H_{35}N_6O_5$	475.26634	475.26548	-1.82
**A_{03}**	$C_{30}H_{45}N_8O_{10}$	677.32532	677.32471	-0.90
**B_{02}**	$C_{28}H_{40}N_7O_{10}$	634.28312	634.28330	0.29
**B_{03}**	$C_{31}H_{45}N_8O_{11}$	705.32023	705.31969	-0.77
**B_{04}**	$C_{35}H_{52}N_9O_{13}$	806.36791	806.36696	-1.18
**B_{05}**	$C_{38}H_{57}N_{10}O_{14}$	877.40502	877.40421	-0.93
**Y''$_{02}$**	$C_{15}H_{28}N_5O_6$	374.20341	374.20342	0.03
**Y''$_{03}$**	$C_{12}H_{23}N_4O_5$	303.16630	303.16583	-1.54
**Y''$_{04}$**	$C_8H_{16}N_3O_3$	202.11862	202.11842	-0.98
**Y''$_{05}$**	$C_5H_{11}N_2O_2$	131.08150	131.08150	-0.03
**Z_{05}**	$C_5H_8N_1O_2$	114.05495	114.05518	1.97

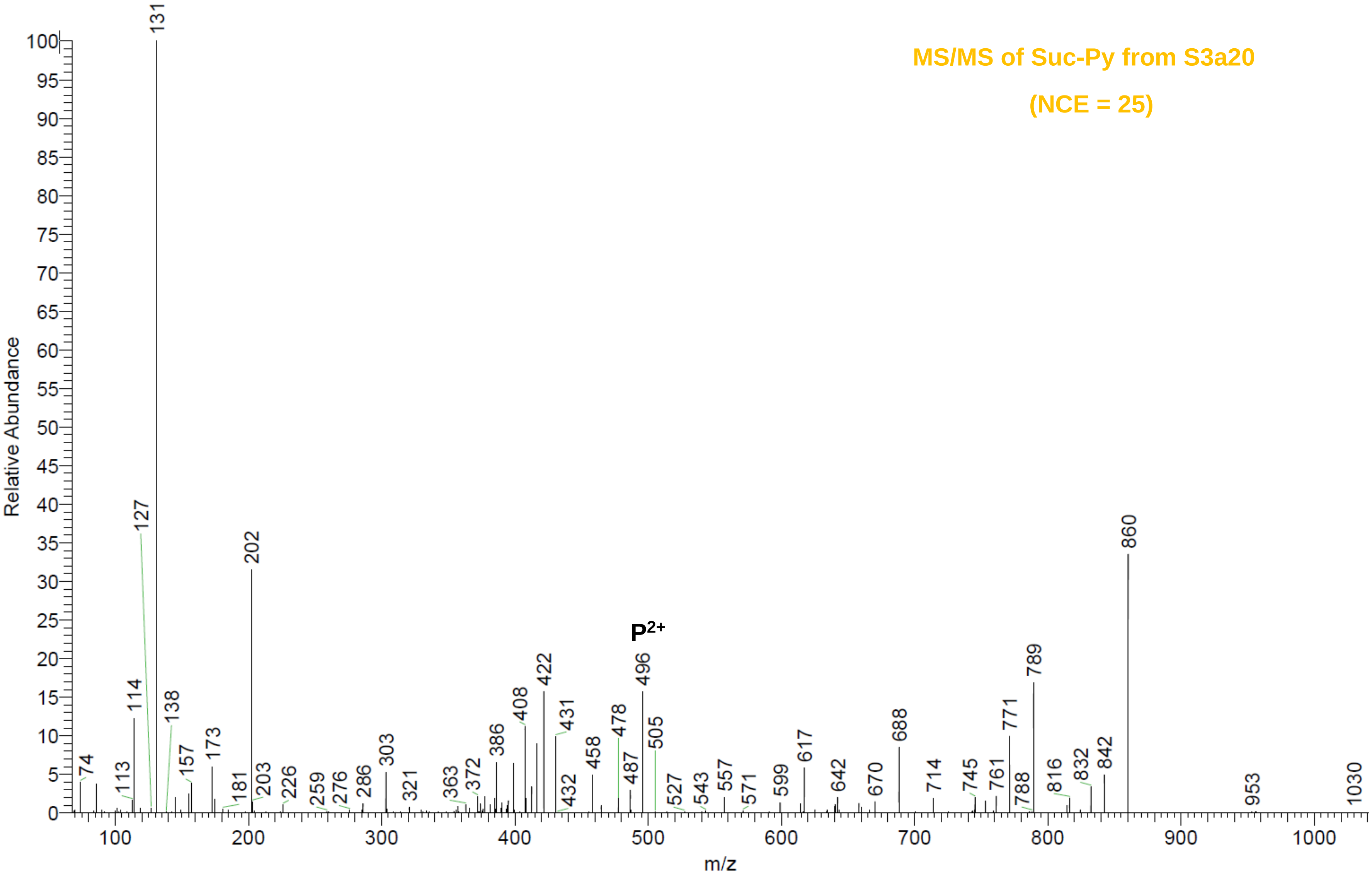
MS/MS of Suc-Py from S3a20
(NCE = 25)
Relative Abundance
m/z
P2+
131
127
202
114
138
157
173
74
113
181
203
226
259
276
286
303
321
363
372
386
408
422
431
432
458
478
487
496
505
527
543
557
571
599
617
642
670
688
714
745
761
771
788
789
816
832
842
860
953
1030

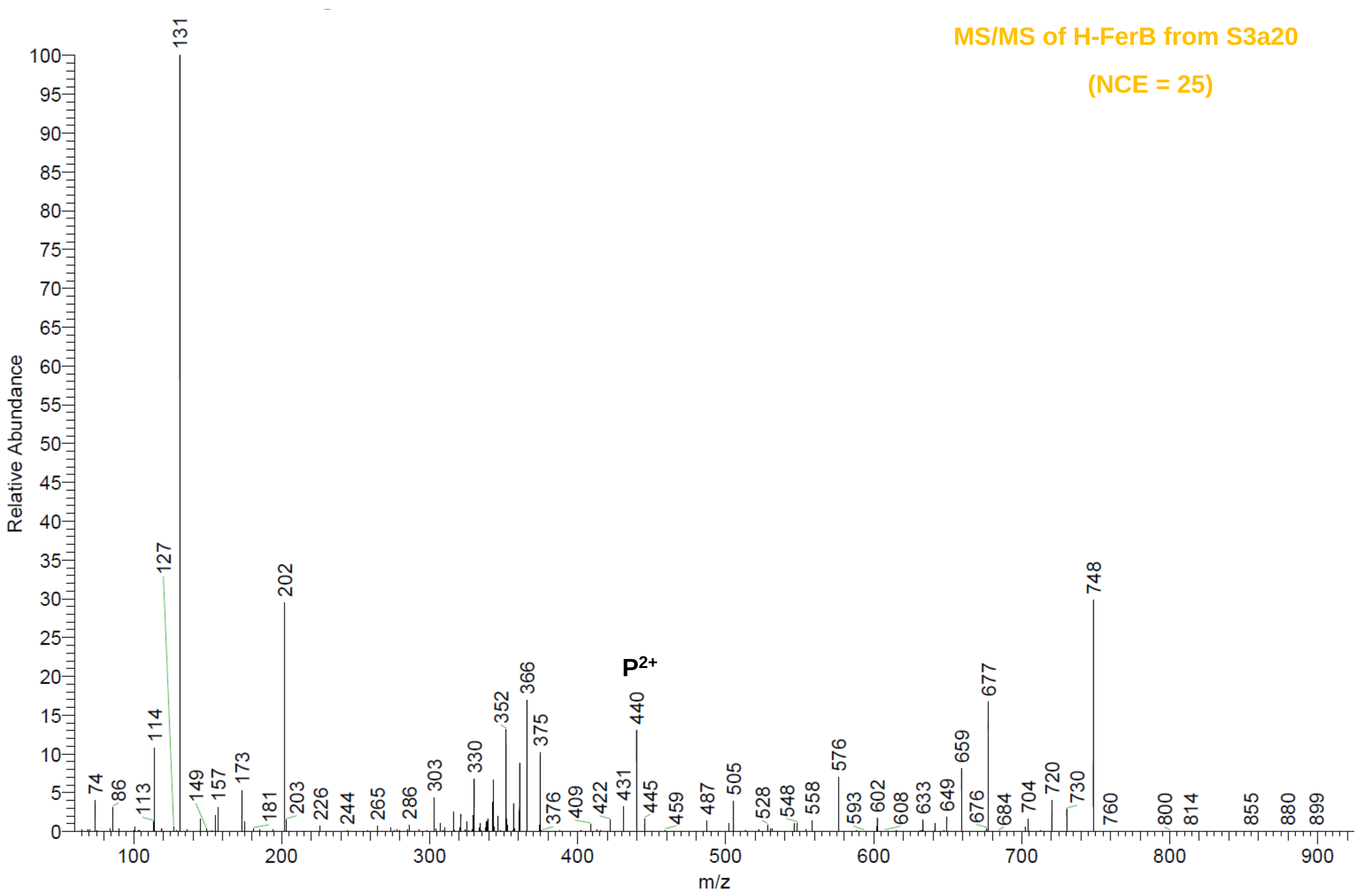

MS/MS of H-FerB from S3a20
(NCE = 25)
Relative Abundance
m/z
P2+
131
127
202
748
677
366
352
440
375
114
576
659
330
303
173
720
505
74
86
113
149
157
181
203
226
244
265
286
376
409
422
431
445
459
487
528
548
558
593
602
608
633
649
676
684
704
730
760
800
814
855
880
899

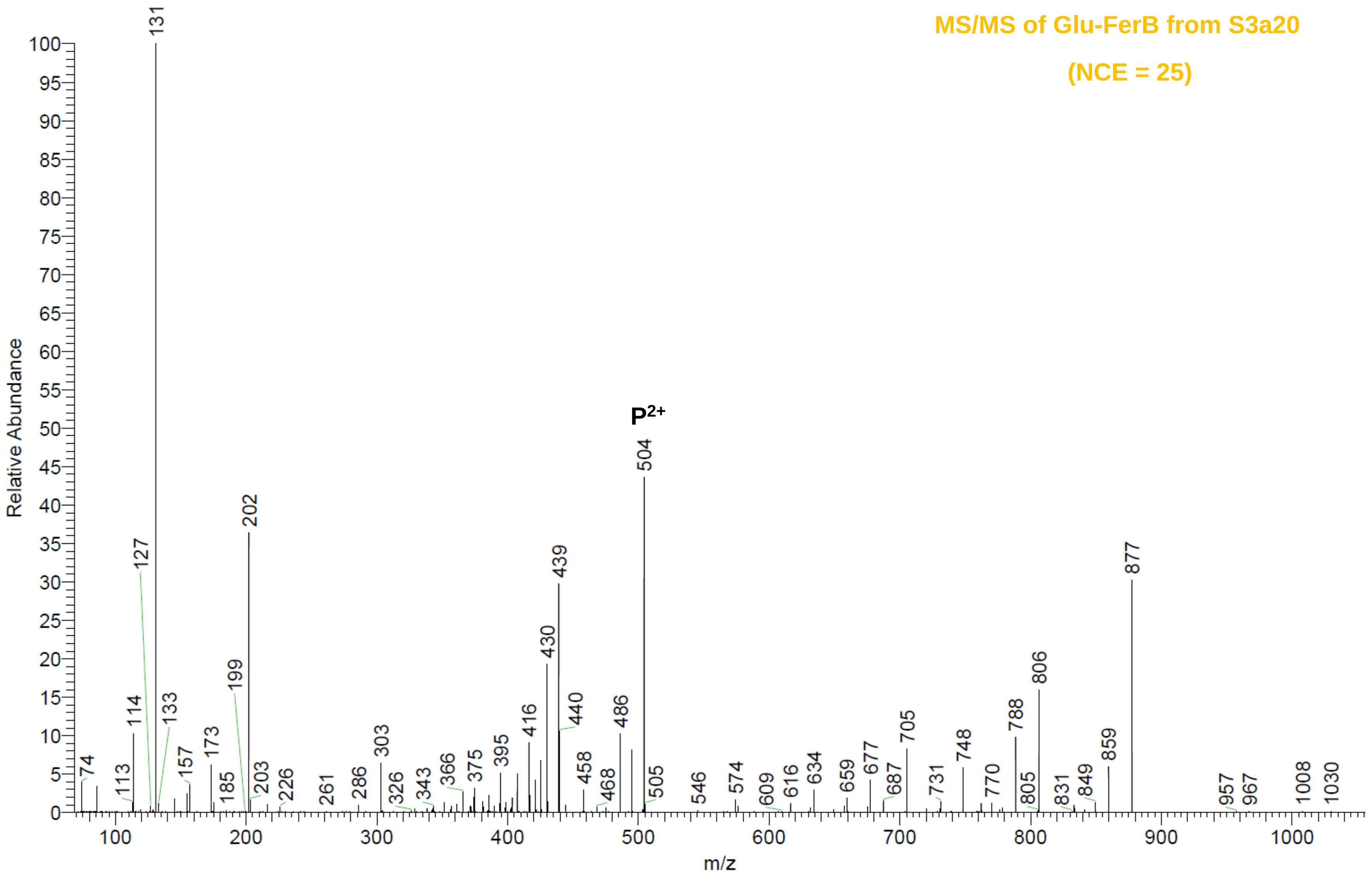
MS/MS of Glu-FerB from S3a20
(NCE = 25)
Relative Abundance
m/z
P2+
131
202
127
504
439
877
430
806
114
133
199
416
440
486
705
788
748
859
173
303
366
375
395
458
634
677
74
113
157
185
203
226
261
286
326
343
468
505
546
574
609
616
659
687
731
770
805
831
849
957
967
1008
1030

Table S15. Detected fragments in the MS/MS fragmentation of Suc-Py of S3b09 compared to their theoretical value *m/z* value and their ppm deviation.

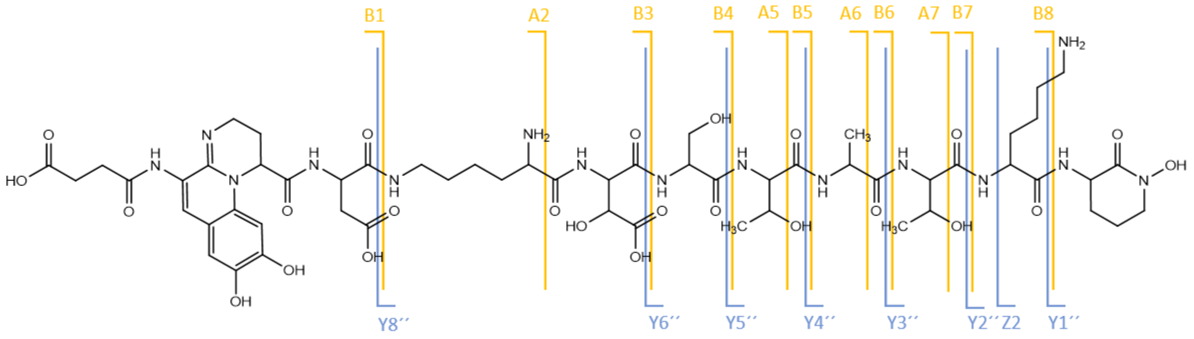

**Name:**	**S3b09**			
**Sequence:**	Asp-ε-Lys-OHAsp-Ser-Thr-Ala-Thr-Lys-cOHOrn			
	**Formula**	**Mass (*m/z*)**	**Found Mass (*m/z*)**	**ppm Deviation**
**[M+H]⁺**	$C_{56}H_{84}N_{15}O_{24}$	1350.58136	1350.57959	-1.31
**[M+2H]²⁺**	$C_{56}H_{85}N_{15}O_{24}$	675.79459	675.79279	-2.67
**Fragment Type (NCE = 25)**	**Formula**	**Mass (*m/z*)**	**Found Mass (*m/z*)**	**ppm Deviation**
**A_{02}**	$C_{26}H_{33}N_6O_9$	573.23035	573.22840	-3.41
**A_{05}**	$C_{37}H_{50}N_9O_{17}$	892.33192	892.32922	-3.02
**A_{06}**	$C_{40}H_{55}N_{10}O_{18}$	963.36903	963.36594	-3.21
**A_{07}**	$C_{44}H_{62}N_{11}O_{20}$	1064.41671	1064.41335	-3.16
**B_{01}**	$C_{21}H_{21}N_4O_9$	473.13030	473.12848	-3.86
**B_{03}**	$C_{31}H_{38}N_7O_{14}$	732.24713	732.24431	-3.85
**B_{04}**	$C_{34}H_{43}N_8O_{16}$	819.27915	819.27658	-3.14
**B_{05}**	$C_{38}H_{50}N_9O_{18}$	920.32683	920.32343	-3.70
**B_{06}**	$C_{41}H_{55}N_{10}O_{19}$	991.36395	991.36126	-2.71
**B_{07}**	$C_{45}H_{62}N_{11}O_{21}$	1092.41162	1092.40869	-2.69
**B_{08}**	$C_{51}H_{74}N_{13}O_{22}$	1220.50659	1220.50242	-3.41
**Y''_{01}**	$C_5H_{11}N_2O_2$	131.08150	131.08118	-2.47
**Y''_{02}**	$C_{11}H_{23}N_4O_3$	259.17647	259.17566	-3.11
**Y''_{03}**	$C_{15}H_{30}N_5O_5$	360.22415	360.22294	-3.35
**Y''_{04}**	$C_{18}H_{35}N_6O_6$	431.26126	431.25966	-3.71
**Y''_{05}**	$C_{22}H_{42}N_7O_8$	532.30894	532.30737	-2.95
**Y''_{06}**	$C_{25}H_{47}N_8O_{10}$	619.34097	619.33848	-4.01
**Y''_{08}**	$C_{35}H_{64}N_{11}O_{15}$	878.45779	878.45505	-3.12
**Z_{02}**	$C_{11}H_{20}N_3O_3$	242.14992	242.14910	-3.38

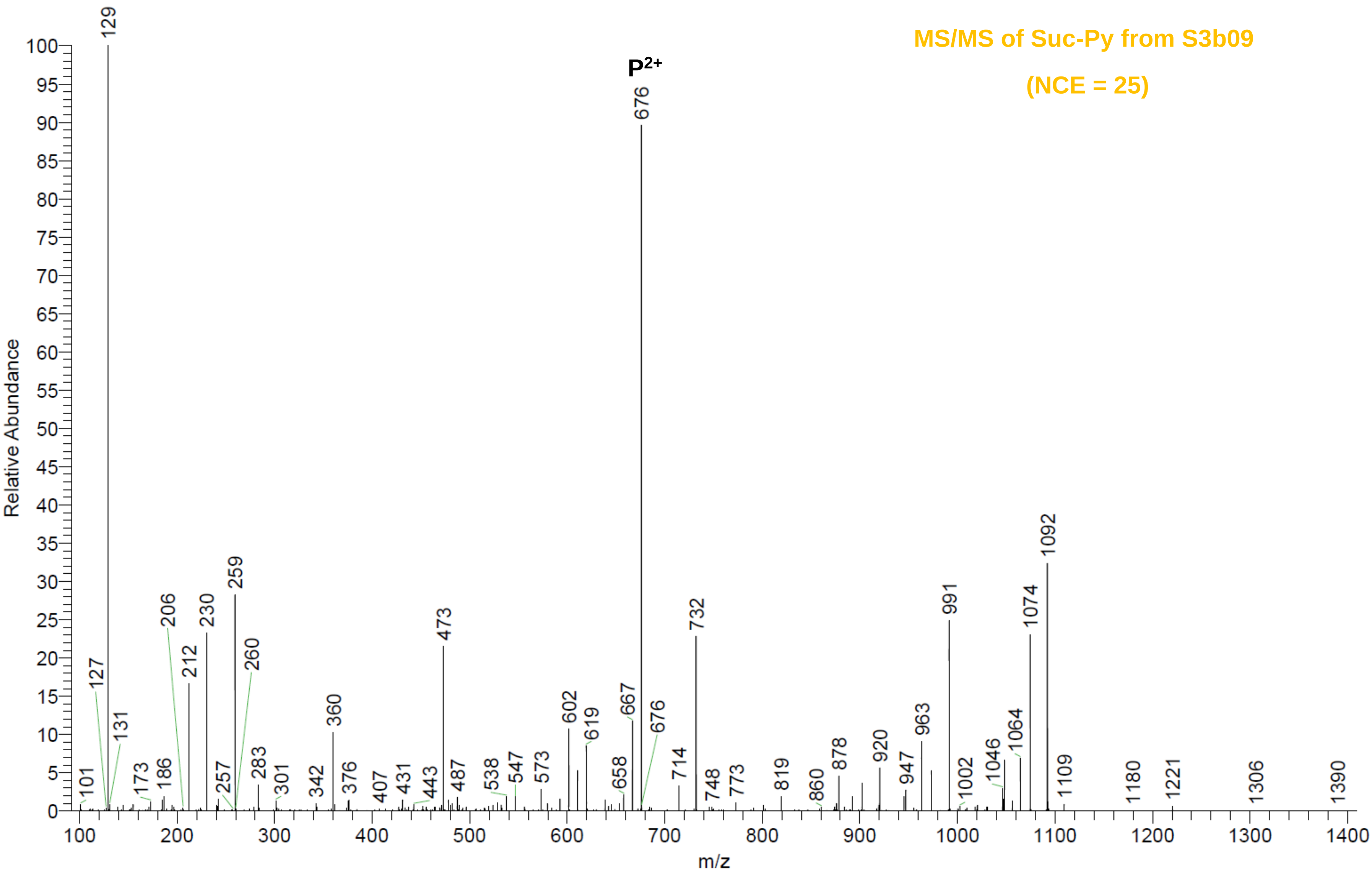
MS/MS of Suc-Py from S3b09
(NCE = 25)
P^{2+}
Relative Abundance
m/z
129
676
1092
259
206
230
212
260
127
131
473
732
991
1074
360
602
619
667
676
101
173
186
257
283
301
342
376
407
431
443
487
538
547
573
658
714
748
773
819
860
878
920
947
963
1002
1046
1064
1109
1180
1221
1306
1390

Table S16. Detected fragments in the MS/MS fragmentation of Suc-Py of S3b16 compared to their theoretical value *m/z* value and their ppm deviation.

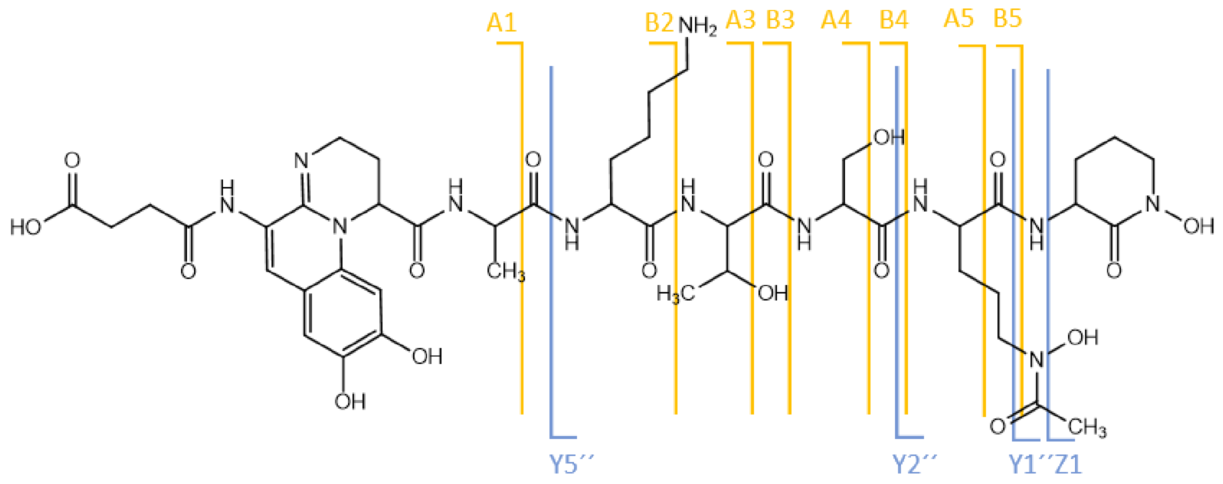

**Name:**	**S3b-16**			
**Sequence:**	Ala-Lys-Thr-Ser-AcOHOrn-cOHOrn			
	**Formula**	**Mass (*m/z*)**	**Found Mass (*m/z*)**	**ppm Deviation**
**[M+H]⁺**	$C_{45}H_{67}N_{12}O_{17}$	1047.47471	1047.47449	-0.21
**$[M+2H]^{2+}$**	$C_{45}H_{68}N_{12}O_{17}$	524.24127	524.24115	-0.23
**Fragment Type (NCE = 30)**	**Formula**	**Mass (*m/z*)**	**Found Mass (*m/z*)**	**ppm Deviation**
**A_{01}**	$C_{19}H_{21}N_4O_6$	401.14556	401.14485	-1.77
**A_{03}**	$C_{29}H_{40}N_7O_9$	630.28820	630.28728	-1.46
**A_{04}**	$C_{32}H_{45}N_8O_{11}$	717.32023	717.31928	-1.33
**A_{05}**	$C_{39}H_{57}N_{10}O_{14}$	889.40502	889.40180	-3.62
**B_{02}**	$C_{26}H_{33}N_6O_8$	557.23544	557.23451	-1.67
**B_{03}**	$C_{30}H_{40}N_7O_{10}$	658.28312	658.28346	0.52
**B_{04}**	$C_{33}H_{45}N_8O_{12}$	745.31515	745.31419	-1.28
**B_{05}**	$C_{40}H_{57}N_{10}O_{15}$	917.39994	917.39947	-0.51
**Y''_{01}**	$C_5H_{11}N_2O_2$	131.08150	131.08146	-0.34
**Y''_{02}**	$C_{12}H_{23}N_4O_5$	303.16630	303.16603	-0.88
**Y''_{05} (25 NCE)**	$C_{25}H_{47}N_8O_{10}$	619.34097	619.34042	-0.88
**Z_{01}**	$C_5H_8N_1O_2$	114.05550	114.05515	-3.10

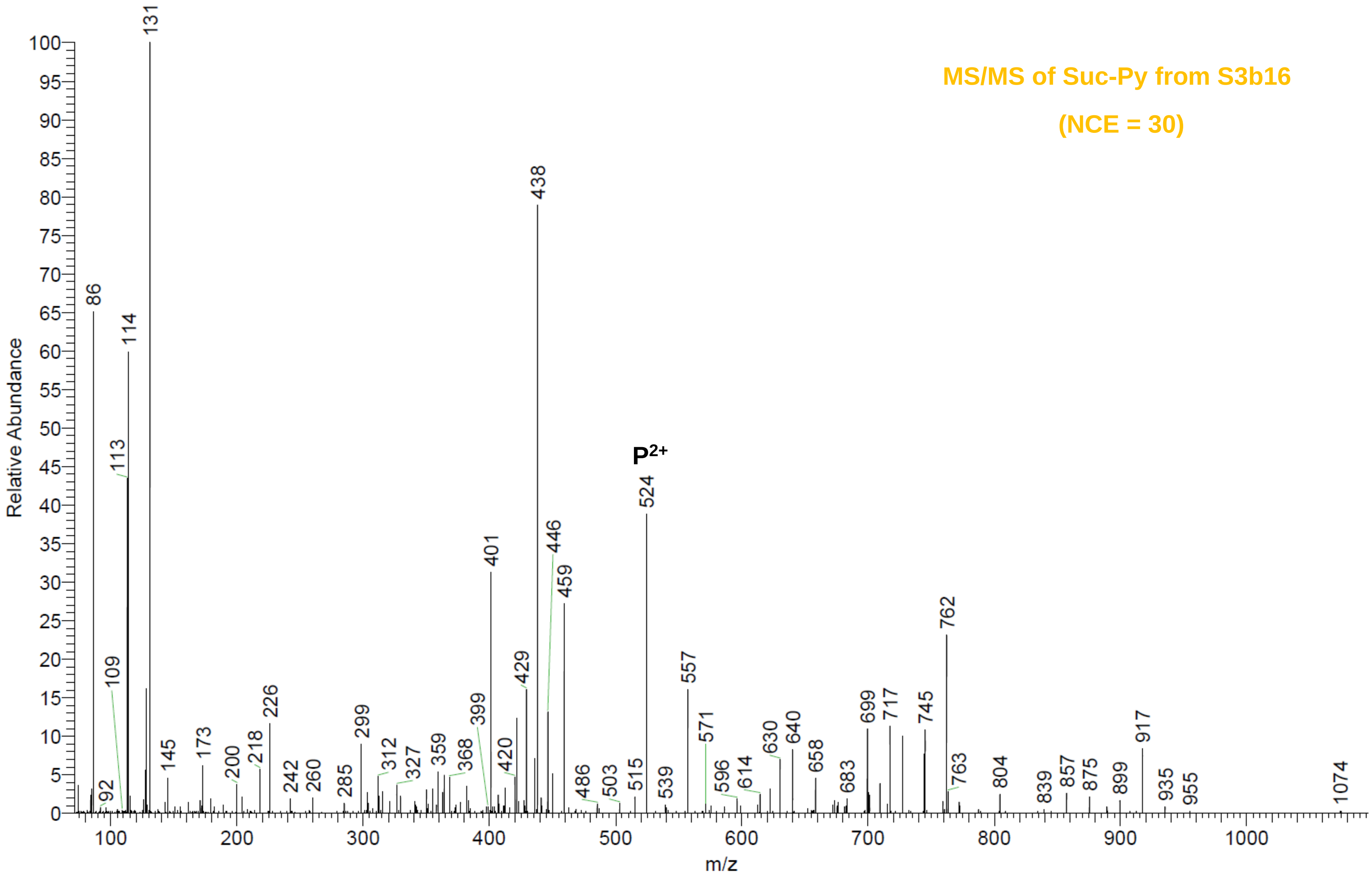
MS/MS of Suc-Py from S3b16
(NCE = 30)
P2+
Relative Abundance
m/z
131
438
86
114
113
524
401
446
459
762
109
429
557
226
173
145
200
218
242
260
92
299
312
327
359
368
399
420
486
503
515
539
571
596
614
630
640
658
683
699
717
745
763
804
839
857
875
899
917
935
955
1074

Table S17. Detected fragments in the MS/MS fragmentation of Suc-Py of S3c13 compared to their theoretical value *m/z* value and their ppm deviation.

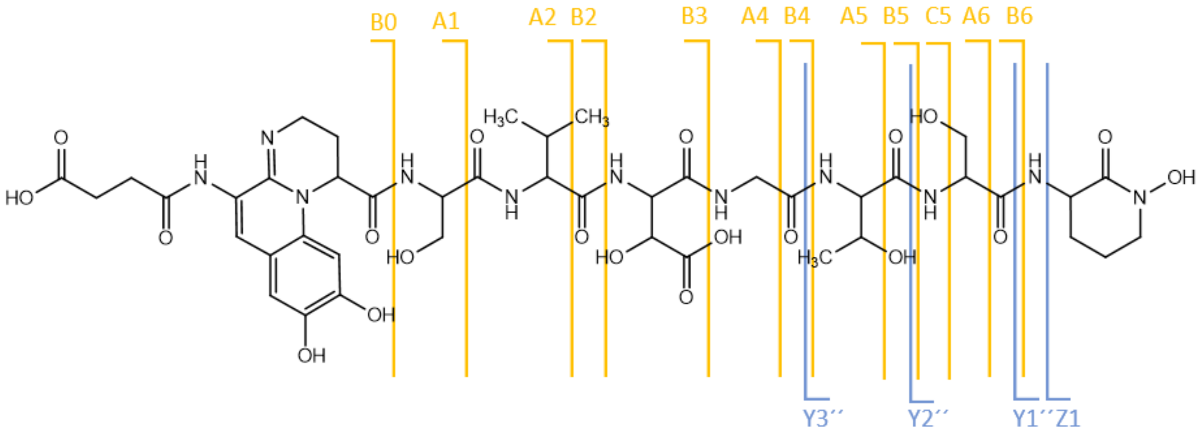

**Name:**	**S3c13**			
**Sequence:**	Ser-Val-OHAsp-Gly-Thr-Ser-cOHOrn			
	**Formula**	**Mass (*m/z*)**	**Found Mass (*m/z*)**	**ppm Deviation**
**[M+H]⁺**	$C_{43}H_{60}N_{11}O_{20}$	1050.40161	1050.40066	-0.90
**[M+2H]²⁺**	$C_{43}H_{61}N_{11}O_{20}$	525.70472	525.70433	-0.74
**Fragment Type (NCE = 25)**	**Formula**	**Mass (*m/z*)**	**Found Mass (*m/z*)**	**ppm Deviation**
**A_{01}**	$C_{19}H_{21}N_4O_7$	417.14048	417.13928	-2.87
**A_{02}**	$C_{24}H_{30}N_5O_8$	516.20889	516.20891	0.04
**A_{04}**	$C_{30}H_{38}N_7O_{13}$	704.25221	704.25092	-1.83
**A_{05}**	$C_{34}H_{45}N_8O_{15}$	805.29989	805.29750	-2.97
**A_{06}**	$C_{37}H_{50}N_9O_{17}$	892.33192	892.32904	-3.23
**B_{00}**	$C_{17}H_{16}N_3O_6$	358.10336	358.10326	-0.28
**B_{02}**	$C_{25}H_{30}N_5O_9$	544.20380	544.20288	-1.70
**B_{03}**	$C_{29}H_{35}N_6O_{13}$	675.22566	675.22370	-2.91
**B_{04}**	$C_{31}H_{38}N_7O_{14}$	732.24713	732.24523	-2.59
**B_{05}**	$C_{35}H_{45}N_8O_{16}$	833.29480	833.29231	-2.99
**B_{06}**	$C_{38}H_{50}N_9O_{18}$	920.32683	920.32477	-2.24
**C_{05}**	$C_{35}H_{46}N_9O_{16}$	848.30570	848.30291	-3.29
**Y''_{01}**	$C_5H_{11}N_2O_2$	131.08150	131.08138	-0.95
**Y''_{02}**	$C_8H_{16}N_3O_4$	218.11353	218.11308	-2.07
**Y''_{03}**	$C_{12}H_{23}N_4O_6$	319.16121	319.16083	-1.19
**Z_{01}**	$C_5H_8N_1O_2$	114.05495	114.05502	0.57

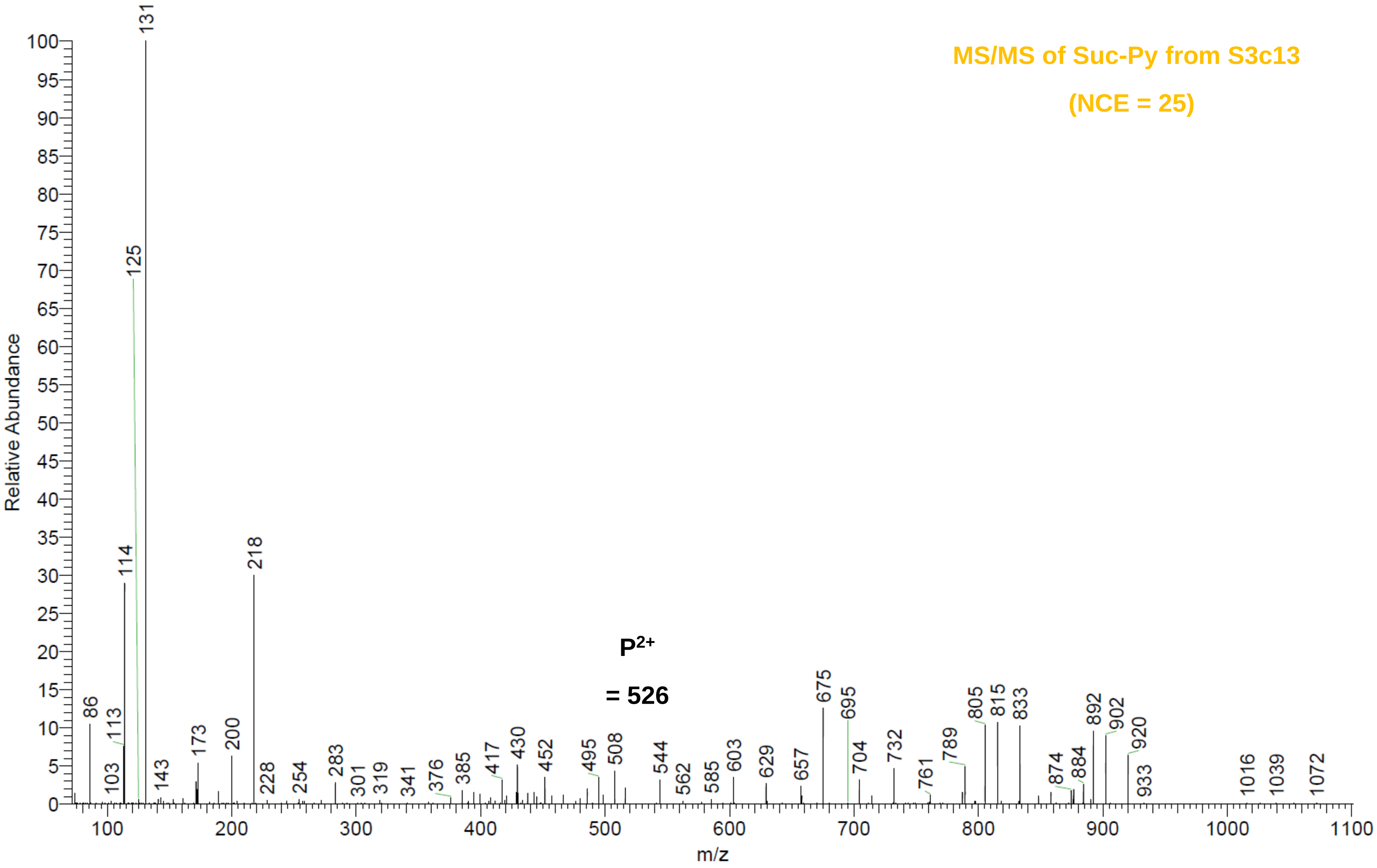
MS/MS of Suc-Py from S3c13
(NCE = 25)
P2+
= 526
Relative Abundance
m/z
86
103
113
114
125
131
143
173
200
218
228
254
283
301
319
341
376
385
417
430
452
495
508
544
562
585
603
629
657
675
695
704
732
761
789
805
815
833
874
884
892
902
920
933
1016
1039
1072

Table S18. Detected fragments in the MS/MS fragmentation of Suc-Py of S3e20 compared to their theoretical value *m/z* value and their ppm deviation.

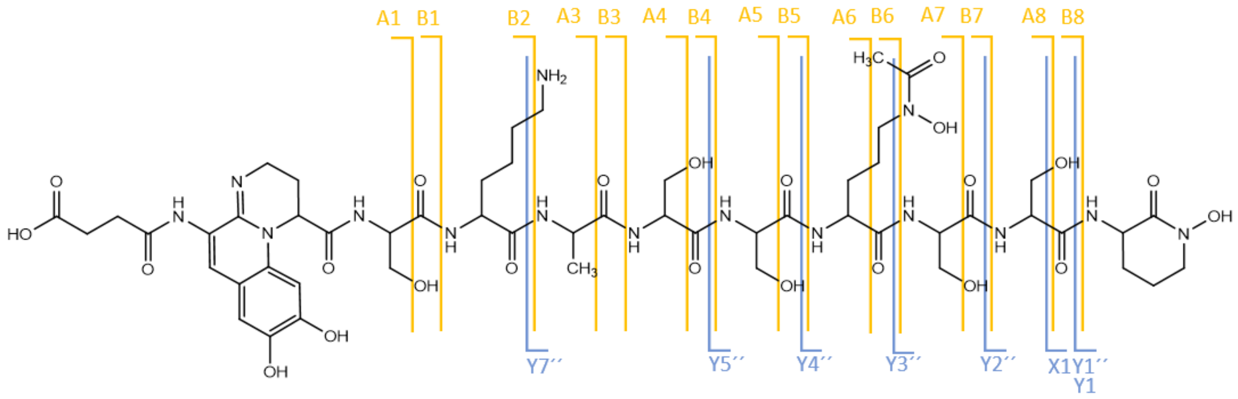

Name:	S3e20			
Sequence:	Ser–Lys–Ala–Ser–Ser–AcOHOrn–Ser–Ser–cOHOrn			
	**Formula**	**Mass (*m/z*)**	**Found Mass (*m/z*)**	**ppm Deviation**
**[M+H]+**	$C_{53}H_{80}N_{15}O_{23}$	1294.55515	1294.55323	-1.48
**[M+2H]2+**	$C_{53}H_{81}N_{15}O_{23}$	647.78149	647.78015	-2.06
**Fragment Type (NCE = 25)**	**Formula**	**Mass (*m/z*)**	**Found Mass (*m/z*)**	**ppm Deviation**
**A_{01}**	$C_{19}H_{21}N_4O_7$	417.14048	417.13957	-2.17
**A_{03}**	$C_{28}H_{38}N_7O_9$	616.27255	616.27022	-3.78
**A_{04}**	$C_{31}H_{43}N_8O_{11}$	703.30458	703.30288	-2.42
**A_{05}**	$C_{34}H_{48}N_9O_{13}$	790.33661	790.33503	-2.00
**A_{06}**	$C_{41}H_{60}N_{11}O_{16}$	962.42140	962.41979	-1.67
**A_{07}**	$C_{44}H_{65}N_{12}O_{18}$	1049.45343	1049.45157	-1.77
**A_{08}**	$C_{47}H_{70}N_{13}O_{20}$	1136.48546	1136.48263	-2.49
**B_{01}**	$C_{20}H_{21}N_4O_8$	445.13539	445.13442	-2.18
**B_{02}**	$C_{26}H_{33}N_6O_9$	573.23035	573.22843	-3.36
**B_{03}**	$C_{29}H_{38}N_7O_{10}$	644.26747	644.26598	-2.31
**B_{04}**	$C_{32}H_{43}N_8O_{12}$	731.29950	731.29768	-2.48
**B_{05}**	$C_{35}H_{48}N_9O_{14}$	818.33152	818.33043	-1.34
**B_{06}**	$C_{42}H_{60}N_{11}O_{17}$	990.41632	990.41480	-1.53
**B_{07}**	$C_{45}H_{65}N_{12}O_{19}$	1077.44834	1077.44659	-1.63
**B_{08}**	$C_{48}H_{70}N_{13}O_{21}$	1164.48037	1164.48036	-0.01
**X_{01}**	$C_6H_9N_2O_3$	157.06077	157.06059	-1.14
**Y_{01}**	$C_5H_9N_2O_2$	129.06585	129.06591	0.43
**Y''_{01}**	$C_5H_{11}N_2O_2$	131.08150	131.08145	-0.41
**Y''_{02}**	$C_8H_{16}N_3O_4$	218.11353	218.11334	-0.88
**Y''_{03}**	$C_{11}H_{21}N_4O_6$	305.14556	305.14503	-1.74
**Y''_{04}**	$C_{18}H_{33}N_6O_9$	477.23035	477.23121	1.80
**Y''_{07}**	$C_{27}H_{48}N_9O_{14}$	722.33152	722.32902	-3.47
**Z_{08}**	$C_5H_8N_1O_2$	114.05495	114.05519	2.06

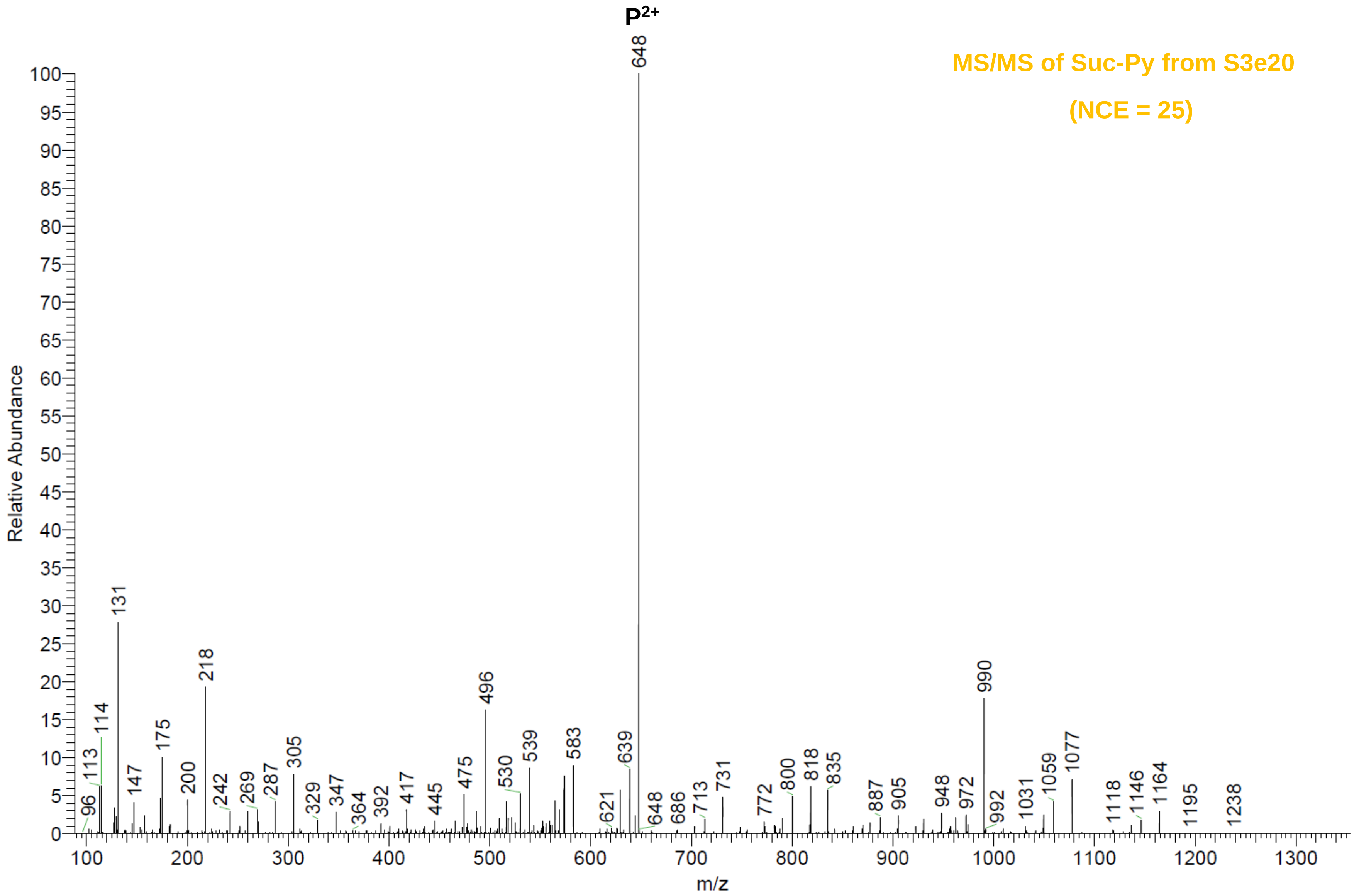
P2+
MS/MS of Suc-Py from S3e20
(NCE = 25)
Relative Abundance
m/z
648
131
218
496
990
114
175
539
583
639
1077
305
113
147
200
242
269
287
329
347
364
392
417
445
475
530
621
648
686
713
731
772
800
818
835
887
905
948
972
992
1031
1059
1118
1146
1164
1195
1238

Table S19. Detected fragments in the MS/MS fragmentation of Suc-Py of S3g01 compared to their theoretical value *m/z* value and their ppm deviation.

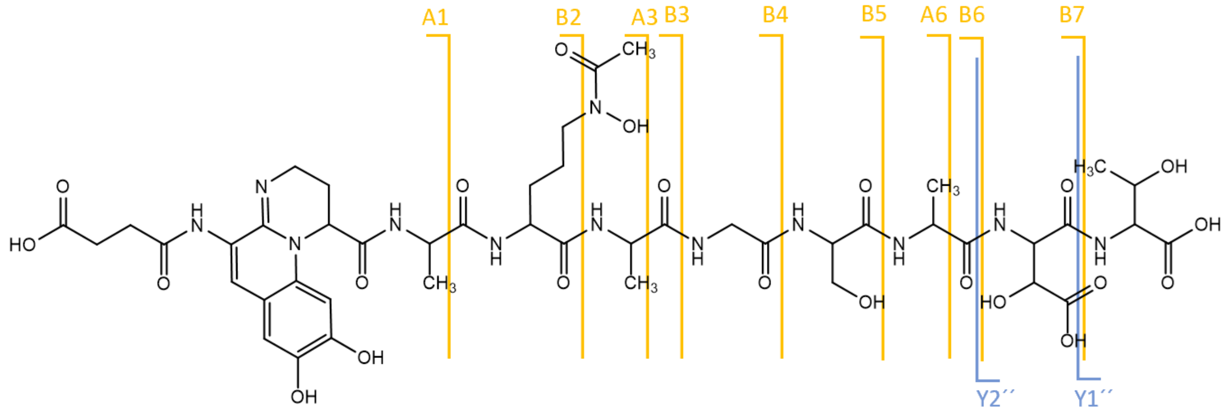

**Name:**	**S3g01**			
**Sequence:**	Ala-AcOHOrn-Ala-Gly-Ser-Ala-OHAsp-Thr			
	**Formula**	**Mass (*m/z*)**	**Found Mass (*m/z*)**	**ppm Deviation**
**[M+H]$^+$**	$C_{46}H_{65}N_{12}O_{22}$	1137.43364	1137.43391	0.24
**[M+2H]$^{2+}$**	$C_{46}H_{66}N_{12}O_{22}$	569.22073	569.22067	-0.11
**Fragment Type (NCE = 25)**	**Formula**	**Mass (*m/z*)**	**Found Mass (*m/z*)**	**ppm Deviation**
**A_{01}**	$C_{19}H_{21}N_4O_6$	401.14556	401.14395	-4.02
**A_{03}**	$C_{29}H_{38}N_7O_{10}$	644.26747	644.26653	-1.45
**B_{02}**	$C_{27}H_{33}N_6O_{10}$	601.22527	601.22608	1.35
**B_{03}**	$C_{30}H_{38}N_7O_{11}$	672.26238	672.26162	-1.13
**B_{04}**	$C_{32}H_{41}N_8O_{12}$	729.28385	729.28398	0.18
**B_{05}**	$C_{35}H_{46}N_9O_{14}$	816.31587	816.31578	-0.111
**B_{06}**	$C_{38}H_{51}N_{10}O_{15}$	887.35299	887.35205	-1.06
**B_{07} (NCE = 20)**	$C_{42}H_{56}N_{11}O_{19}$	1018.3748	1018.37438	-0.46
**Y''_{02}**	$C_8H_{15}N_2O_7$	251.08738	251.08682	-2.22
**Y''_{01}**	$C_4H_{10}N_1O_3$	120.06552	120.06566	1.17

MS/MS of Suc-Py from S3g01

(NCE = 25)

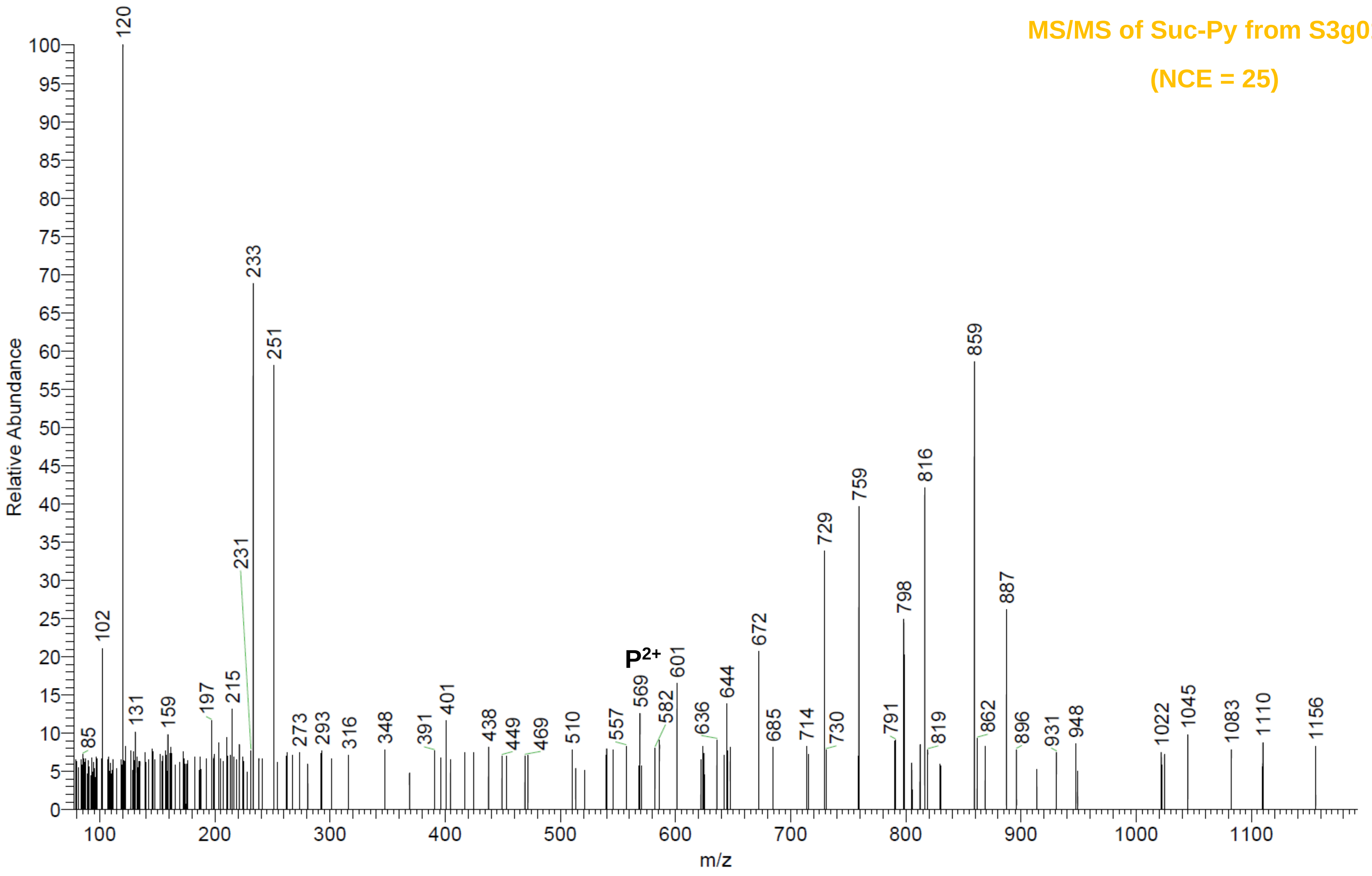

Supplement: Supplementary file 2 — Supplementary file2 (PDF 2708 KB) [file 216_2022_3907_MOESM2_ESM.pdf]
